# Graphs of study contributions and covariate distributions for network meta‐regression

**DOI:** 10.1002/jrsm.1292

**Published:** 2018-02-14

**Authors:** Sarah Donegan, Sofia Dias, Catrin Tudur‐Smith, Valeria Marinho, Nicky J. Welton

**Affiliations:** ^1^ Department of Biostatistics, Waterhouse Building University of Liverpool 1‐5 Brownlow Street Liverpool L69 3GL UK; ^2^ School of Social and Community Medicine University of Bristol Canynge Hall, 39 Whatley Road Bristol BS8 2PS UK; ^3^ Barts and The London School of Medicine and Dentistry Institute of Dentistry 4 Newark Street London E1 2AT UK

**Keywords:** contribution, meta‐regression, network meta‐analysis, extrapolationtreatment by covariate interactionsweight

## Abstract

**Background:**

Meta‐regression results must be interpreted taking into account the range of covariate values of the contributing studies. Results based on interpolation or extrapolation may be unreliable. In network meta‐regression (NMR) models, which include covariates in network meta‐analyses, results are estimated using direct and indirect evidence; therefore, it may be unclear which studies and covariate values contribute to which result. We propose graphs to help understand which trials and covariate values contribute to each NMR result and to highlight extrapolation or interpolation.

**Methods:**

We introduce methods to calculate the contribution that each trial and covariate value makes to each result and compare them with existing methods. We show how to construct graphs including a network covariate distribution diagram, covariate‐contribution plot, heat plot, contribution‐NMR plot, and heat‐NMR plot. We demonstrate the methods using a dataset with treatments for malaria using the covariate average age and a dataset of topical fluoride interventions for preventing dental caries using the covariate randomisation year.

**Results:**

For the malaria dataset, no contributing trials had an average age between 7–25 years and therefore results were interpolated within this range. For the fluoride dataset, there are no contributing trials randomised between 1954–1959 for most comparisons therefore, within this range, results would be extrapolated.

**Conclusions:**

Even in a fully connected network, an NMR result may be estimated from trials with a narrower covariate range than the range of the whole dataset. Calculating contributions and graphically displaying them aids interpretation of NMR result by highlighting extrapolated or interpolated results.

## 1 INTRODUCTION

Network meta‐analyses have become increasingly popular in recent years in terms of application in systematic reviews and methodological developments.^1^, ^2^, ^3^, ^4^, ^5^ Network meta‐regression (NMR) is an extension of network meta‐analysis, which examines whether several treatment effects (eg, log odds ratios) differ according to a covariate (eg, trial setting).^6^ For instance, if 3 treatments exist for a particular condition (treatments *1, 2, 3*), NMR could simultaneously examine whether the treatment effect for *2* vs *1* varies according to a covariate, whether the effect for *3* vs *1* varies according to the same covariate, and whether the effect for *3* vs *2* also varies. NMR results from the NMR model commonly consist of, for each comparison, 1 treatment effect estimated at the covariate value zero (or at the mean covariate value when the NMR model is centred) and 1 regression coefficient for the treatment by covariate interaction.

When inconsistency (ie, variability across treatment comparisons) or heterogeneity (ie, variability across trials that directly compare the same 2 treatments) is detected in a network meta‐analysis, the results of the meta‐analysis may not be valid. In such cases, NMR can be used to explore causes of the heterogeneity and/or inconsistency, and if the variability is reduced or disappears in the NMR, the results from the NMR may be more meaningful than those from the network meta‐analysis and may be used to draw reliable clinical inferences. Yet, when heterogeneity and/or inconsistency is still present in the NMR, the results of the NMR may be unreliable, and it may be more appropriate to reconsider the eligibility criteria or explore other covariates. Moreover, NMR can be valuable when stratified results for different patient groups are required to answer clinical questions. For example, results were stratified by seizure type (ie, patients suffering from partial or generalised seizures) in a NMR of epilepsy drugs.^7^, ^8^ In these circumstances, NMR can estimate treatment effects at different covariate values, facilitating stratified medicine for different patient groups. For categorical covariates (such as, surgical procedure), the treatment effects for each category can be estimated (eg, treatment effects for studies involving amputees and treatment effects for studies of breast surgery). For continuous covariates (eg, trial duration), it is possible to calculate treatment effects at any value of the covariate (eg, at weeks 0, 4, 8, 12, 16, 20,....). Stratified analyses or meta‐regressions are often used in reviews to explore patient‐level covariates (eg, patient age) using trial‐level summaries (eg, average age) or trial‐level covariates (eg, study location).^9^, ^10^

However, when interpreting the results of a meta‐regression, it is important to consider the covariate values of the included trials so that interpolated or extrapolated results are identified. Results that rely on interpolation or extrapolation may not be reliable and could lead to drawing incorrect conclusions that affect clinical practice. In a standard pair‐wise meta‐regression, where trials' results for only 1 treatment comparison are regressed against trials' covariate values, it is relatively simple to identify when results are interpolated or extrapolated.^6^, ^11^, ^12^ For example, if the dose of a drug ranged from 100 to 150 mg/day and 300 to 400 mg/day in the studies, the results would be applicable for doses 100 to 150 mg/day and 300 to 400 mg/day, but we would be less confident about applying the results (interpolated) for doses 150 to 300 mg/day, and even less confident in applying results (extrapolated) for those over 400 mg/day. However, understanding the contribution of different covariate values to results is more complicated for NMR because results are estimated based on a combination of direct and indirect evidence. For example, for the comparison *2* vs *1*, the trials that directly compare *2* vs *1* may have covariate values for dose ranging from 100 to 200 mg/day, but the trials that contribute indirect evidence (eg, *3* vs *1* and *3* vs *2*) may have covariate values ranging from 200 to 300 mg/day; therefore, the results for that comparison may be considered to be reliably estimated for doses 100 to 300 mg/day. For the comparison *3* vs *1*, different trials with different covariate values may contribute to results for that comparison, etc. Furthermore, for large treatment networks, trials that are closely connected to a particular treatment comparison in the network (eg, first‐order indirect evidence) may contribute more to the NMR result for that comparison than trials that are further away in the network (eg, third‐order indirect evidence).^13^ Therefore, to avoid drawing misleading conclusions in NMR, it is important to identify which trials contribute to each NMR estimate, by how much they contribute, and consider their covariate range so that each result can be interpreted with the relevant covariate range in mind. To our knowledge, although NMR methodology has been widely introduced and applied, such issues regarding interpretation have not been discussed in published literature.^6^, ^8^, ^14^, ^15^, ^16^, ^17^, ^18^

In this article, we introduce novel methods to help understand which trials and covariate values contribute to each NMR result (ie, each treatment effect at zero covariate and each regression coefficient) and to highlight extrapolated or interpolated results. We propose new methods to calculate the percentage contribution that each trial makes to each NMR result. The methods can tell us, for example, that a particular result is 10% based on trials with covariate values 0 to 100 mg/day, 70% from trials with values 100 to 200 mg/day, and 20% from trials with values 200 to 300 mg/day. Therefore, we can consider the covariate range relevant to each NMR result. The proposed methods to calculate contributions were inspired by an existing fixed‐effect Frequentist method that involved estimating the pairwise meta‐analytic treatment effects based on direct evidence and calculating the contribution each pairwise estimate makes to each network meta‐analytic estimate in the absence of covariates.^19^, ^20^, ^21^ Caldwell et al also used similar methodology to calculate the precision of a network meta‐analysis treatment effect.^13^ We extend the method to compute the contribution each *trial* makes to each NMR treatment effect estimate and to each regression coefficient estimate for a treatment by covariate interaction.

Recently, methods were proposed for calculating study weights in multi‐parameter meta‐analysis.^22^, ^23^ Neither article presented methods specifically for NMR or discussed contributions of covariate values, extrapolation, or interpolation. Yet, such methods can be applied to NMR without modification. We compare the new methods proposed here in with those presented by Riley et al.^23^

The methods introduced in this paper are applicable for both continuous and categorical covariates and trial‐level aggregate outcome data. In Section 2, we introduce 2 datasets that are used to illustrate the methods. We outline existing NMR models in Section 3. In Section 4, we introduce new methods for calculating the contribution that each trial makes to each NMR result and compare them with those presented by Riley et al.^23^ New methods are described for fixed‐effect and random‐effects models, Bayesian, and Frequentist frameworks, and models that make different assumptions regarding the regression coefficients for the treatment by covariate interactions. In Section 5, we propose novel graphs that will help interpret NMR results with the covariate distribution and contributions in mind. In Section 6, we discuss the proposed methods and findings.

## 2 ILLUSTRATIVE DATASETS

The proposed methods will be demonstrated using 2 example aggregate datasets; a dataset of 3 treatments for severe malaria with a dichotomous outcome where the treatment effects are measured on the log odds ratio scale and another dataset of topical fluoride interventions for preventing dental caries, which is a much larger network, involving a continuous outcome with treatment effects measured by the standardised mean difference (SMD).

The malaria dataset was constructed using 2 Cochrane reviews and trial reports; 1 review compared artesunate (AS) versus quinine (QU) and the second compared artemether (AR) versus QU and AS versus AR.^24^, ^25^ Both reviews included randomised controlled trials including patients with severe malaria. Results were stratified by age in the reviews, and therefore age was considered to be a treatment effect modifier. Event rates for the primary outcome, death, were extracted from the reviews and data were cross‐checked against the trial reports. The covariate, average age of patients, in each trial was extracted from the trial reports. Two studies with missing covariate data were deleted from the dataset. Log odds ratios and their standard errors were calculated for each trial in R using the event rates. Table S1 displays the data.

The fluoride dataset was originally constructed using several Cochrane reviews and has been used previously to demonstrate network meta‐analysis methods in the methodological literature.^17^, ^26^, ^27^, ^28^, ^29^, ^30^, ^31^, ^32^, ^33^ Reviews included randomised or quasi‐randomised controlled trials that used or indicated blind outcome assessment and compared different forms of topical fluoride interventions for preventing dental caries in children or adolescents with a duration of at least 1 year or school year. Six treatments were compared, that is, no treatment (NT), placebo (PL), fluoride in dentifrice (DE), fluoride in rinse (RI), fluoride in gel (GE), and fluoride in varnish (VA). The primary outcome was caries increment in permanent teeth measured by the change from baseline in decayed, missing, and filled tooth surfaces. For each trial, for each treatment group, the number of participants, mean caries increment, and the corresponding standard deviation were obtained. The covariate, randomisation year, of each trial was also obtained. Specifically, the year of randomisation was taken to be the same as the year the study began but for trials where the year a study began was not accessible it was estimated by subtracting the duration of the trial (in years) plus 1 extra year from the publication year. Previously, Salanti et al found an interaction between treatment effect and randomisation year and we explore this interaction further.^17^ As in the previous article, SMDs were used to compare treatments. SMDs, their standard errors and covariances (for multi‐arm trials) were calculated for each trial in R using formulae specified by Cooper et al.^34^ See Table S2 for the data.

## 3 NETWORK META‐REGRESSION MODELS

### 3.1 Model specification

Let *i* denote the trial where *i* = 1, ……, *N* and *N* is the number of independent trials and let *k* be the trial arm where *k* = 1, ……, *A*_*i*_ and *A*_*i*_ is the number of arms in trial *i*. Let *t*_*ik*_ denote the treatment given in trial *i* in arm *k* where *t*_*ik*_ ∈ {1, ……, *T*} and *T* is the number of treatments in the network. Note that treatment *1* is taken to be the reference treatment.

Suppose we have trial‐level outcome data, where *y*_*ik*_ is the observed treatment effect (eg, log odds ratio) for arm *k* vs arm *1* (with *k* ≥ 2) in trial *i* and *v*_*ik*_ is the corresponding variance. We assume a normal likelihood *y*_*ik*_~*N*(*θ*_*ik*,_*v*_*ik*_) where *θ*_*ik*_ is the mean treatment effect in trial *i* (with *k* ≥ 2). Let *c*_*i*_ be a study‐level covariate for trial *i* (such as, a continuous covariate value or an indicator variable for a dichotomous covariate).

There are 3 different assumptions that can be made regarding the basic regression coefficients for the treatment by covariate interactions, that is, they are independent, exchangeable, or common.^6^, ^14^, ^15^, ^35^ The basic regression coefficients are the coefficients for each treatment versus the reference treatment *1* (ie, *β*_12_, *β*_13_, …, *β*_1*T*_, where, for example, *β*_12_ is the regression coefficient for treatment *2* versus treatment *1*). The decision regarding which assumption is most appropriate for a specific dataset can be based on the model fit, data availability, the resulting estimates of the regression coefficients, and clinical judgement.

The NMR model with independent interactions can be written as

$$\theta_{\mathrm{ik}}=\delta_{\mathrm{ik}}+\beta_{t_{i1},t_{\mathrm{ik}}}c_{i}$$

where $\beta_{t_{i1},t_{\mathrm{ik}}}$= $\beta_{1,t_{\mathrm{ik}}}$‐ $\beta_{1,t_{i1}}$, $\beta_{t_{i1},t_{\mathrm{ik}}}$ is the difference in the treatment effect of *t*_*ik*_ vs *t*_*i*1_ per unit increase in the covariate *c*_*i*_, or in other words, the regression coefficient for the treatment by covariate interaction for *t*_*ik*_ vs *t*_*i*1_. In a random‐effects model, *δ*_*ik*_ (with *k* ≥ 2) represents the trial‐specific treatment effect treatment effect in trial *i* for arm *k* vs arm *1* when the covariate is zero (or when the covariate is the mean value if the model is centred at the mean) and is assumed to be a realisation from a normal distribution where $\delta_{\mathrm{ik}}∼Ν\left(d_{\mathrm{ti}1,t_{\mathrm{ik}}},\sigma^{2}\right)$with $d_{t_{i1},t_{\mathrm{ik}}}=d_{1,t_{\mathrm{ik}}}-d_{1,t_{i1}}$ and $d_{t_{i1},t_{\mathrm{ik}}}$ is the mean treatment effect of *t*_*ik*_ vs *t*_*i*1_ when the covariate is zero (or when the covariate is the mean value for centred models). Here, the between trial variance *σ*^2^ is assumed to be the same for each comparison; this assumption is often made in the network meta‐analysis literature and applications to aid estimation.

In a fixed‐effect model, we set *σ*^2^ = 0 to obtain *δ*_*ik*_= $d_{1,t_{\mathrm{ik}}}-d_{1,t_{i1}}$. The NMR model with exchangeable interactions is given by letting $\beta_{1,t_{\mathrm{ik}}}\sim \text{Norm}\left(B,\upsilon^{2}\right)$ and the model with common interactions is formulated by setting $\beta_{1,t_{\mathrm{ik}}}=\beta$. With common interactions, the functional regression coefficients (ie, $\beta_{t_{i1},t_{\mathrm{ik}}}$ where *t*_*i*1_ ≠ 1) are fixed to be zero.^35^

When multi‐arm trials contribute, the correlation between the observed treatment effects (*y*_*ik*_) and the trial‐specific treatment effects (*δ*_*ik*_) from the same study must be taken into account in the modelling; details are described in the supplementary material.

### 3.2 Application to datasets

NMR models were fitted using WinBUGS 1.4.3 and the R2WinBUGS package in R. Fixed‐effect and random‐effects models including independent, exchangeable, and common interactions were applied. The correlation between treatment effects from the same trial was taken into account in the models when multi‐arm trials existed. The covariates were centred at their mean. All parameters were given non‐informative normal prior distributions (ie, *N*(0, 100000)) except the between trial standard deviation that was assumed to follow a non‐informative uniform distribution (ie, *Uni*(0, 10)). Three chains with different initial values were run for 300 000 iterations. The initial 100 000 draws were discarded, and chains were thinned such that every fifth iteration was retained. See supplementary code S1.

Model fit and complexity of fixed‐effect and random‐effects models was assessed using the deviance information criterion (DIC) defined as $\mathrm{DIC}=\bar{D}+p_{D}$ where $p_{D}=\bar{D}-\hat{D}$ and $\bar{D}$ was the posterior mean residual deviance, *p*_*D*_ was the effective number of parameters, and $\hat{D}$ was the deviance evaluated at the posterior mean of the model parameters.^36^ A model with a comparatively smaller DIC was preferable to a model with a larger DIC; when the difference in DIC was very small, the difference was not considered meaningful; and in this case, the simpler fixed‐effect models were preferred to random‐effects models and the model with independent interactions was preferred because it makes the weakest assumption regarding the coefficients.

#### 3.2.1 Malaria dataset

The results from the fixed‐effect NMR with independent interactions are shown in Table S3. Results from this model are presented because the DICs from each applied model were similar (DICs 22.95–26.13). There is evidence of an interaction between log odds ratio and average age for AS vs QU. As the average age ranged from 2.33 to 34.47 years across trials, log odds ratios at mean age, 0, 5, 10, 15, 20, 25, 30, and 35 years are displayed. For AS vs AR, none of the displayed log odds ratios indicate a difference between the drugs. However, each of the log odds ratios suggests a difference between AS and QU, and a difference between AR and QU is observed for log odds ratios estimated for age 15 years or more. The results of the NMR could be used to draw clinical inferences because an interaction has been found. However, to aid interpretation, we must first consider which trials and covariate values contribute to each result to be aware of extrapolation and interpolation.

#### 3.2.2 Fluoride dataset

Table S4 displays the results from the random‐effects NMR model including independent interactions. This model provided a lower DIC (DIC = 546.67) than the fixed‐effects models with independent (DIC = 797.41), exchangeable (DIC = 798.82), and common interactions (DIC = 809.14); results from the random‐effects models were similar (DIC = 546.67–547.04). The posterior median of the between trial variance is 0.03 with 95% credibility interval (0.02, 0.05). The results show that there is an interaction between the SMD and randomisation year for VA vs PL. SMDs at year 1954, 1960, 1970, 1980, 1990, and 1994 are presented because the randomisation year across all the trials ranged from 1954 to 1994. For DE, RI, GE, vs NT and DE vs PL, there is a difference between the 2 interventions being compared for each displayed SMD. For VA vs NT and RI vs PL, a difference is found for SMDs estimated for years 1954, 1960, 1970, 1980, and 1990. Whereas, for PL vs NT, a difference is found between 1970 and 1990, and for GE and VA vs PL, a difference is observed between 1954 and 1980. Salanti et al concluded that “older studies gave more enthusiastic results for the effectiveness of fluoride” and the NMR results in our article agree with this conclusion.^17^ However, to further explore the results, we need to consider which trials and covariate values contribute to each result.

## 4 CALCULATING THE TRIALS' CONTRIBUTIONS

### 4.1 Methods

In the supplementary material, details are provided regarding the new methods for calculating the percentage contribution that each trial makes to each NMR result (ie, each $d_{t_{i1},t_{\mathrm{ik}}}$ and each $\beta_{t_{i1},t_{\mathrm{ik}}}$). For each NMR result, the amount that each trial contributes to the result is estimated; the contributions across all trials sum to 100%. The methods can be used assuming either fixed or random treatment effects, and assuming independent, exchangeable, or common interactions. Methods are described for a Frequentist approach and Bayesian framework including prior information. In a Bayesian framework, the contribution that the prior distributions make to each NMR result can also be calculated, which may be useful when informative prior distributions are used. The methods can be applied to datasets that include multi‐arm trials because they can allow for the correlation between treatment effects from the same trial.

A summary of the existing methods proposed by Riley et al to calculate trial contributions is also given in the supplementary material.^23^ The existing methods have been applied in a Frequentist framework, can assume either fixed or random treatment effects, and accommodate multi‐arm trials. However, at present, the existing methods do not allow for the inclusion of prior information in a Bayesian setting. The current methods can calculate the contribution that each trial makes to each basic treatment effect (ie, $d_{t_{i1},t_{\mathrm{ik}}}$ where *t*_*i*1_ = 1) and to each basic regression coefficient (ie, $\beta_{t_{i1},t_{\mathrm{ik}}}$ where *t*_*i*1_ = 1). Providing the model's results does not depend on the choice of reference treatment, the contribution that each trial makes to each functional treatment effect (ie, $d_{t_{i1},t_{\mathrm{ik}}}$ where *t*_*i*1_ ≠ 1), and each functional coefficient (ie, $\beta_{t_{i1},t_{\mathrm{ik}}}$ where *t*_*i*1_ ≠ 1) can be calculated by re‐applying the methods with different treatments coded as the reference treatment. Therefore, the existing methods cannot be used assuming exchangeable or common interactions because the results of such models can differ depending on the choice of reference treatment.

### 4.2 Application to datasets

We used R and Excel to calculate the studies' contributions using the new methods. The methods by Riley et al were applied using Stata and verified using R.^23^ The covariates were centred at their mean. Computing code is supplied (code S2).

#### 4.2.1 Malaria dataset

Using the new methods, the contribution of each trial to each log odds ratio and each regression coefficient is shown in Table S5. Study contributions vary between 0 and 49.86%. Notice that each contribution column sums to 100%. Generally, a trial will contribute different amounts to each NMR result, for example, van Hensbroek 1996 contributes 25.70% to the log odds ratio of AR vs QU and 14.00% to the coefficient for AS vs AR. Also, different trials contribute different amounts to each NMR result, for example, for the coefficient for AS vs QU, Dondorp 2010 contributes 46.26% whereas Adam 2002 contributes 0.00%. It is clear that the contribution a study makes to a log odds ratio is generally similar but not identical to the contribution it makes to the corresponding regression coefficient, for example, van Hensbroek 1996 contributes 25.70% to the log odds ratio of AR vs QU and 21.00% to the coefficient for AR vs QU.

Table S6 displays the study weights for each NMR result calculated using the existing methods proposed by Riley et al.^23^ Contributions were similar but not identical to those calculated using the new methods with absolute differences between results varying from 0 to 15.29%. The NMR results obtained from Stata were the same as those in Table S3.

#### 4.2.2 Fluoride dataset

The contribution of each trial to each SMD and each regression coefficient is displayed in Table S7 using the new methods. Study contributions vary between 0% and 12.7%; therefore, no single study dominates a particular NMR result. Analogous to the malaria dataset, a trial will contribute different amounts to each NMR result, different trials contribute different amounts to each NMR result, and the contribution a study makes to a log odds ratio differs from the contribution it makes to the corresponding coefficient.

Note that when the NMR model was refitted in Stata to calculate the study weights using the existing Frequentist methods proposed by Riley et al, the NMR results differed marginally (see Table S8).^23^ Table S9 displays the study weights for each NMR result calculated from the existing method. The contributions from the existing method were not exactly the same as those from the new method. The absolute differences between results varied from 0 to 18.64%.

## 5 GRAPHICAL DISPLAYS

The proposed graphs aid interpretation of NMR results by displaying the covariate distributions or study contributions. Graphs include a network covariate distribution diagram, covariate‐contribution plot, heat plot, contribution‐NMR plot, and heat‐NMR plot.

Covariate‐contribution plots, heat plots, contribution‐NMR plots, and heat‐NMR plots were constructed in R. Example code is provided in the supplementary material (code S3‐S6). We chose to use the contributions calculated from the new methods in the graphs but equally, the contributions estimated by the methods of Riley et al could be used.^23^

### 5.1 The network covariate distribution diagram

#### 5.1.1 Graph description

A standard network diagram displays nodes and edges; the nodes represent the treatments and each edge (ie, a connecting line), which join 2 nodes, represents the availability of outcome data from studies that directly compare the 2 treatments. Such diagrams are widely used to visually display the available evidence and can be constructed using various software and display options.^19^, ^37^ The proposed network covariate distribution diagram is an adaptation of the standard network diagram in that the covariate values of trials are also displayed on the diagram. For each edge (ie, treatment comparison with direct evidence), a histogram of the covariate values can be drawn with the edge considered analogous to the horizontal axis of the histogram.

Furthermore, for large networks, the diagrams can become cluttered; therefore, instead, histograms of the covariate values can be drawn alongside the network diagram. Alternatively, a 3‐dimensional version of the diagram can be drawn, with the treatment network drawn on a 2‐dimensional plane and the covariate distributions plotted in a third dimension; Batson et al propose a similar diagram and have produced a computer package to draw such graphs.^38^

The purpose of the network covariate distribution diagram is to visualise the covariate values in addition to the evidence base. For each comparison, it is useful to understand the range of covariate values contributing direct evidence because NMR parameter estimation issues may be identified, for example, it would not be possible to fit a model with independent interactions when only 1 trial contributes to a basic regression coefficient or when all the studies that contribute to a basic coefficient have the same covariate value. Also, interpolation and extrapolation may be detected from the diagram when no trials with covariate values within a particular covariate range exist. However, it is difficult to draw conclusions regarding the overall covariate range relevant for each comparison from this diagram because indirect evidence also contributes to the NMR results.

#### 5.1.2 Application to datasets

### 5.2 Malaria dataset

**Figure 1:**
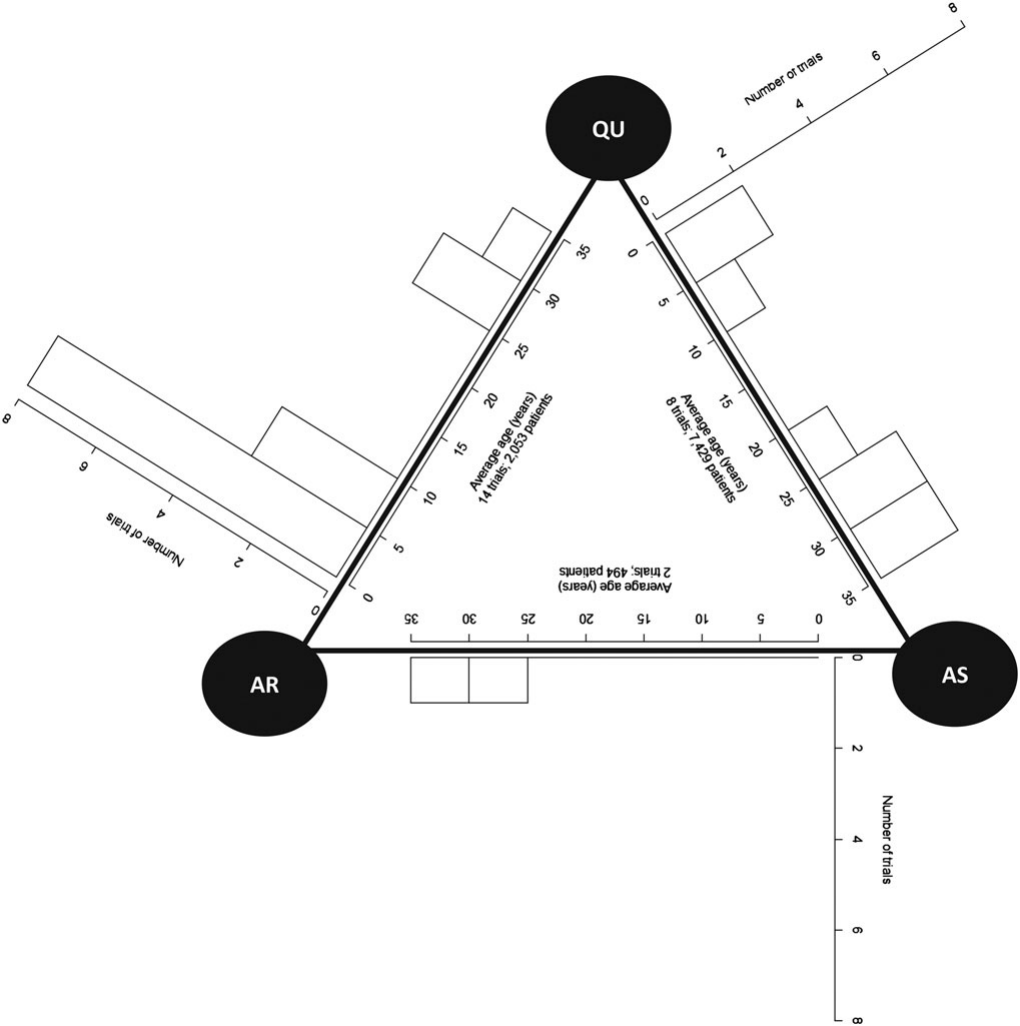
Network covariate distribution diagram for the malaria dataset

Figure 1 shows the network covariate distribution diagram. As this dataset is a 3 treatment network, histograms can be drawn into the diagram. It is apparent that the distribution of the average ages of the patients in trials that contribute direct evidence differs across comparisons. There are no paediatric trials directly comparing AS vs AR. However, this may not be a concern because the network is a 3 treatment loop; therefore, all the trials in the network and their covariate values should contribute to all NMR results, but it is not obvious by how much each trial contributes to each NMR result. Furthermore, we see that no trials have an average age between approximately 10 and 20 years, so results are interpolated within this range; however, if some trials contribute very little to some results, the range for interpolation may be wider than this for some comparisons.

### 5.3 Fluoride dataset

**Figure 2:**
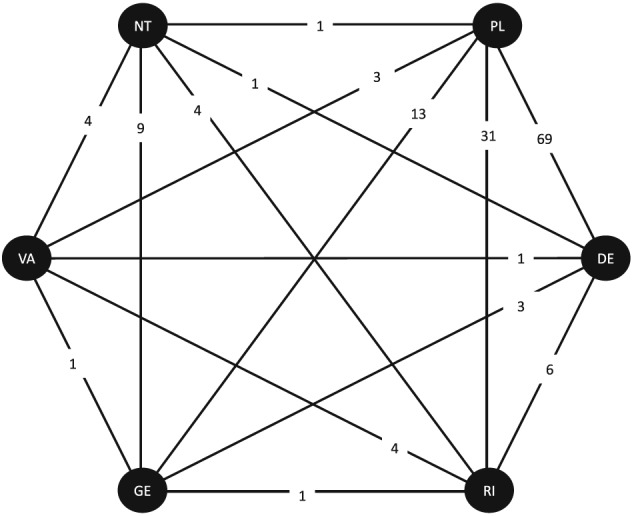
Network diagram for the fluoride dataset.DE: dentifrice; GE: gel; NT: no treatment; PL: placebo; RI: rinse; VA: varnish. Numbers of studies contributing direct evidence are displayed

**Figure 3:**
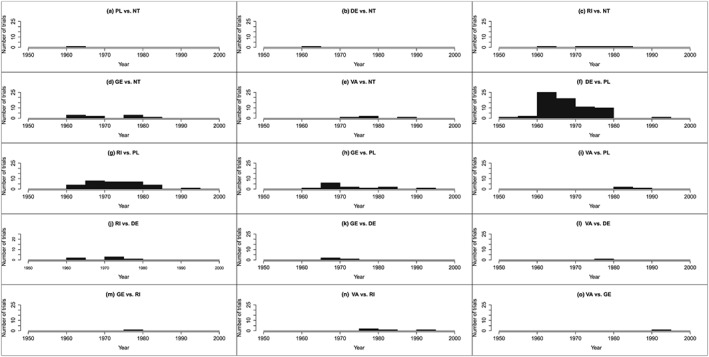
Distributions of randomisation year for the fluoride dataset.DE: dentifrice; GE: gel; NT: no treatment; PL: placebo; RI: rinse; VA: varnish

As the fluoride dataset is a larger network, a network diagram including covariate data is cluttered (diagram not presented), and therefore it is preferable to present covariate information separately to the diagram. Figure 2 shows the network diagram, and Figure 3 presents covariate information. There are certainly differences in the distribution of randomisation year across comparisons with many comparisons having a very narrow range of years (Figure 3). However, the network is fully connected (ie, every intervention is directly compared with every other intervention); therefore, one would expect all trials to make some contribution to each NMR result.

### 5.4 The covariate‐contribution plot

#### 5.4.1 Graph description

A covariate‐contribution plot consists of 1 graph per NMR result, that is 1 graph for each treatment effect at zero covariate and each regression coefficient. For each NMR result, the percentage contribution that each trial makes to the NMR result is plotted on the vertical axis against the covariate value for each study on the horizontal axis; 1 point per study is displayed on the graph. Various display options can be considered in the plot. The study number can be displayed in the graph rather than a standard plotting point symbol so that the contribution a particular study makes to different NMR results can be compared.

Moreover, for large networks, where the number of NMR results is large, 1 graph per comparison can be constructed, rather than 1 graph per NMR result, with the contributions to the treatment effect and the contributions to the coefficient displayed on a single graph but using different colours or plotting symbols.

The aim of the plot is to show which trials and covariate values contribute to each result. A key advantage of the plot is that if extrapolation or interpolation exists, it is clearly visible from this plot. Furthermore, it can be useful to know which studies and covariate values contribute to which results when considering causes of any existing inconsistency.

#### 5.4.2 Application to datasets

### 5.5 Malaria dataset

**Figure 4:**
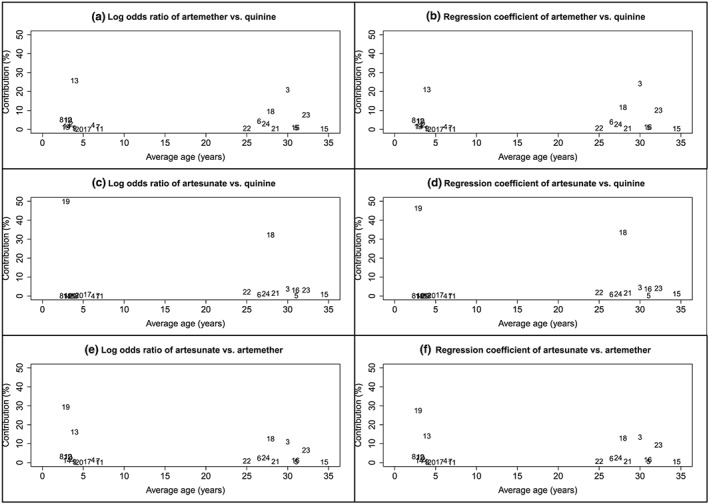
Covariate‐contribution plot showing average age versus percentage contribution of each trial to each log odds ratio and each regression coefficient for the malaria dataset.Numbers represents the study number in Table S1

The contribution of each trial to each log odds ratio and each regression coefficient is shown in the covariate‐contribution plot in Figure 4. For this dataset, we present separate plots showing the contributions to the log odds ratios (Figure 4A,C,E) and regression coefficients (Figure 4B,D,F), and we display the study numbers instead of points. Figure 4 shows that no trials have an average age between 7 and 25 years, and therefore results are interpolated within this range for each NMR result. For each comparison, specific studies do seem to dominate the NMR result. For example, for AS vs QU, studies 18 and 19 contribute 32.22% and 49.86%, respectively, to the log odds ratio and 33.52% and 46.26% to the coefficient; the average ages of patients in these studies are 27.90 and 2.85 years. Therefore, we may be more confident in drawing conclusions for these average ages. Whereas, studies with average ages of 3 to 7 years, 25 to 27 years, and over 28 years contribute little for AS vs QU; therefore, we may be less confident about interpreting the result within this range (Figure 4C,D).

### 5.6 Fluoride dataset

**Figure 5:**
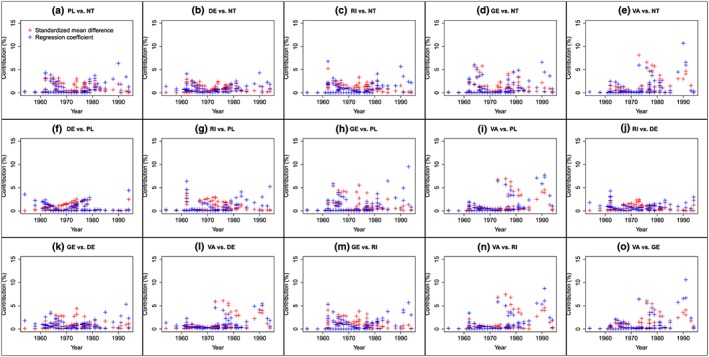
Covariate‐contribution plot showing randomisation year versus percentage contribution of each trial to each SMD and each regression coefficient for the fluoride dataset.DE: dentifrice; GE: gel; NT: no treatment; PL: placebo; RI: rinse; VA: varnish [Colour figure can be viewed at http://wileyonlinelibrary.com]

Figure 5 is the covariate‐contribution plot showing the contribution of each trial to each SMD and each regression coefficient. As many treatments are compared in this dataset, we chose to present 1 graph per comparison and red points to represent the contributions to the SMDs and blue points to represent the contributions to the regression coefficients. The figure clearly shows that, for each comparison, a wide range of covariate values contribute to the NMR results with no obvious areas of interpolation or extrapolation. For most comparisons, the percentage contributions are relatively similar across covariate values such that the contributions do not decrease or increase with increasing randomisation year. Also, no single study dominates a particular comparison, that is, all contributions are less than 15%.

### 5.7 Heat plot

#### 5.7.1 Graph description

For continuous covariates, to construct the heat plot, first, suitable ranges of the covariate are chosen (eg, trial duration 12–24 weeks, 24–36 weeks,,,….). Then, for each NMR result, the contributions can be summed across trials within the same covariate range to give the contribution of the covariate range to the result. For instance, for a particular NMR result, the contributions of trials that have a covariate value between 12 and 24 weeks would be summed and similarly, summed for trials with values between 24 and 36 weeks; from this, we may find that the result is 80% from trials with values 12 to 24 weeks and 20% from trials with values 24 to 36 weeks. For categorical covariates, a similar approach is taken by summing the contributions of trials within each covariate category.

The summed contributions are then displayed on the heat plot. The heat plot displays how much each covariate range contributes to each NMR result using a matrix format. Each cell of the matrix shows the contribution of a covariate range to an NMR result by displaying the numerical contribution and using colour shading (eg, lower contributions represented using blue shades and higher contributions represented using red shades). The heat plot is particularly useful for highlighting covariate ranges where an NMR result is extrapolated or interpolated.

#### 5.7.2 Application to datasets

### 5.8 Malaria dataset

**Figure 6:**
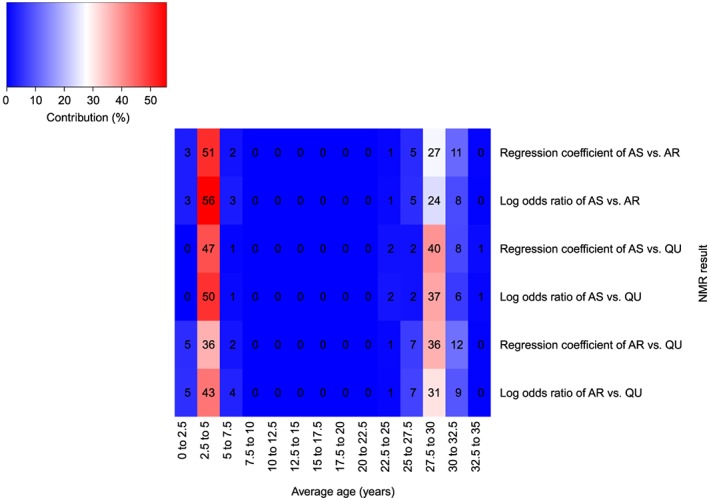
Heat plot showing average age versus the NMR result for the malaria dataset.Block colour and numbers represent the contribution of each covariate range to each log odds ratio and each regression coefficient (%).AR: artemether; AS: artesunate; QU: quinine [Colour figure can be viewed at http://wileyonlinelibrary.com]

Figure 6 shows the heat plot. Trials were grouped according to the average age of patients using intervals of 2.5 years, ranging from zero to 35 years. For AS vs AR and AR vs QU, the contributing trials have average ages within range 0 to 7.5 years and 22.5 to 32.5 years; therefore, results would be interpolated or extrapolated outside of these ranges. Similarly, for AS vs QU, trials within range 2.5 to 7.5 years and 22.5 to 35 years contribute to results. The plot shows that results between 7.5 and 22.5 years are interpolated for all comparisons. Within each age group, the contributions vary across NMR results, for instance, trials with average age between 2.5 and 5 years contribute 36% to the regression coefficient for AR vs QU but contribute 51% to the regression coefficient for AS vs AR.

### 5.9 Fluoride dataset

**Figure 7:**
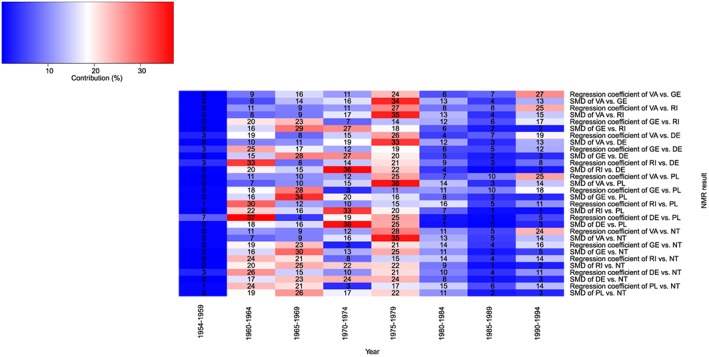
Heat plot showing randomisation year versus the NMR result for the fluoride dataset. Block colour and numbers represent the contribution of each covariate range to each SMD and each regression coefficient (%)DE: dentifrice; GE: gel; NT: no treatment; PL: placebo; RI: rinse; VA: varnish [Colour figure can be viewed at http://wileyonlinelibrary.com]

The heat plot is shown in Figure 7. Trials were grouped with respect to randomisation year using intervals of 5 years. For most NMR results, the majority of the contributing trials have randomisation years between 1960 and 1979. There are no contributing trials randomised between 1954 and 1959 for most NMR results, and there are no contributing trials randomised between 1985 and 1989 for 1 NMR result; therefore, these results would be extrapolated if interpreted within these ranges. The observed contributions vary across NMR results within each randomisation year range, as well as varying across randomisation year ranges for each NMR result.

### 5.10 The contribution‐NMR plot

#### 5.10.1 Graph description

The aim of the contribution‐NMR plot is to display the results of the NMR as well as the study data points in a similar way to a bubble plot that is often used to display standard pair‐wise meta‐regression. This enables the NMR results to be interpreted while also considering the covariate distributions.

The contribution‐NMR plot consists of 1 graph per treatment comparison. Each graph has 2 sections.

The bottom section of the graph is a plot of the treatment effect on the vertical axis versus the covariate value on the horizontal axis. The NMR regression line (and its 95% confidence or credibility interval) for that comparison is drawn to show the NMR treatment effects estimated at various covariate values. Points plotted for each study that contributes direct evidence at the observed trial's treatment effect and covariate value.

The top section of the graph displays a plot of covariate value on the horizontal axis. Points are plotted for each study contributing indirect evidence to that comparison at the observed covariate value. Notice that there are no observed treatment effects for trials contributing indirect evidence; therefore, the points could not plotted in the bottom section but can be plotted in the top section because there is no treatment effect scale on the vertical axis.

To display the contributions on the plot, the size of the contribution that a study makes to a NMR result can be represented by the size of a point for that study. For a particular comparison, the size of the contribution that a study makes to the treatment effect at zero covariate may differ from the contribution it makes to the corresponding regression coefficient; therefore, 2 points of different sizes must be plotted in the same place because they represent the same study. We recommend using a circle as the plotting symbol so that the 2 points for each study can both be seen simultaneously even when they are over‐layered on the plot; a red circle whose size represents the contribution of a study to the treatment effect and a blue circle whose size represents the contribution of the same study to the regression coefficient. When the circles are large and do not fit inside the plotting region, the contribution values can be rescaled by dividing all contributions by an appropriate scalar value so that circles fit in the region and are visually pleasingly.

However, it is worth noting that to simplify the plot, information regarding contributions can be ignored such that 1 point per study is plotted where all plotting points have the same standard size.

The contribution‐NMR plot shows which trials and covariate values contribute most to each NMR result, whether results are evidence based or have been interpolated or extrapolated and whether treatment by covariate interactions exist. Furthermore, differences between the covariate distribution of trials contributing direct evidence and the covariate distribution of trials contributing indirect evidence for a particular comparison are obvious from the plot, and as such, the plot facilitates exploration of causes of inconsistency.

#### 5.10.2 Application to datasets

### 5.11 Malaria dataset

**Figure 8:**
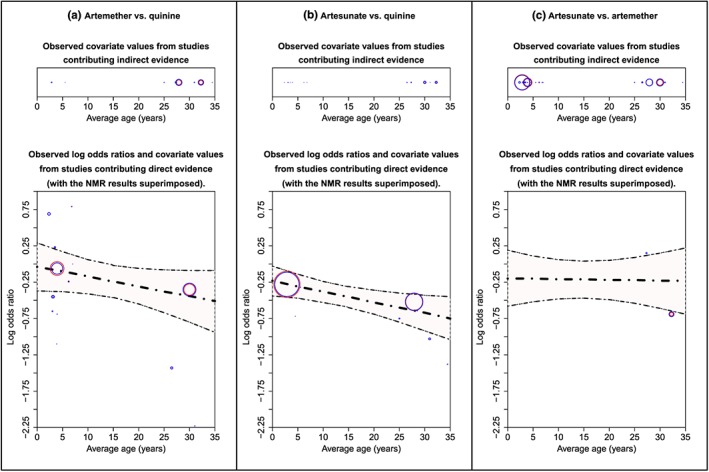
Contribution‐NMR plot for the malaria dataset. The bold dot‐dash line is the log odds ratio, and the 2 dashed lines are the upper and lower 95% credibility intervals estimated by the model. Points (ie, circles) represent the trials that contribute to the model estimates; points for trials that contribute direct evidence are displayed in the bottom section, and points from trials that contribute indirect evidence are displayed in the top section. The size of the red circle represents the size of the contribution that a trial makes to the log odds ratio (estimated at mean value), and the size of the blue circle represents the size of the contribution that a trial makes to the regression coefficient. Larger circles represent larger contributions [Colour figure can be viewed at http://wileyonlinelibrary.com]

Figure 8 displays the contribution‐NMR plot. Contributions were scaled by a fifth. Results are interpolated between around average age 7 to 25 years. For AR vs QU, results are mostly based on 2 direct evidence trials and 2 indirect evidence trials approximately within ranges 0 to 5 years and 28 to 35 years. For AS vs QU, 2 direct evidence trials mostly contribute to the results around range 0 to 5 years and 25 to 30 years. Results for AS vs AR are mostly based on indirect evidence trials approximately within range 0 to 7 years and 28 to 35 years. Figure S1 shows the same plot but without presenting the study contributions.

### 5.12 Fluoride dataset

**Figure 9:**
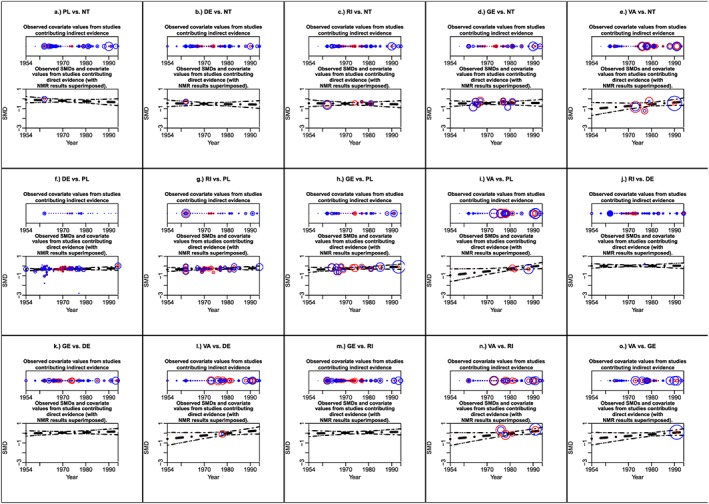
Contribution‐NMR plot for the fluoride dataset. The bold dot‐dash line is the SMD, and the 2 dashed lines are the upper and lower 95% credibility intervals estimated by the model. Points (ie, circles) represent the trials that contribute to the model estimates; points for trials that contribute direct evidence are displayed in the bottom section, and points from trials that contribute indirect evidence are displayed in the top section. The size of the red circle represents the size of the contribution that a trial makes to the SMD (estimated at mean value), and the size of the blue circle represents the size of the contribution that a trial makes to the regression coefficient. Larger circles represent larger contributions. DE: dentifrice; GE: gel; NT: no treatment; PL: placebo; RI: rinse; SMD: standardised mean difference; VA: varnish [Colour figure can be viewed at http://wileyonlinelibrary.com]

The contribution‐NMR plot is shown in Figure 9. Contributions were scaled by a half. For all comparisons except 7 (ie, PL vs NT, DE vs NT, DE vs PL, RI vs PL, RI vs DE, GE vs DE, and VA vs DE), there are no contributing trials randomised before 1960; therefore, results are extrapolated for low randomisation years. The plot clearly shows that for many comparisons (ie, PL vs NT, DE vs NT, RI vs DE, GE vs DE, VA vs DE, GE vs RI, VA vs GE), NMR results are based on no or very limited direct evidence. A simpler version on the plot that does not display the study contributions is shown in Figure S2.

### 5.13 Heat‐NMR plot

#### 5.13.1 Graph description

The contribution‐NMR plot was devised to simultaneously display the results of the NMR and the contributions of various covariate ranges to the NMR results so that the NMR results can be interpreted with the covariate distribution in mind.

The heat‐NMR plot has 1 graph per treatment comparison. Like the contribution‐NMR plot, for each treatment comparison, a graph of treatment effect on the vertical axis versus covariate value on the horizontal axis is constructed with the NMR regression line and its 95% confidence or credibility interval displayed.

However, in the heat‐NMR plot, the area between the upper and lower confidence or credibility bounds is coloured. The summed contributions calculated for the heat plot are used to colour the area so that the covariate ranges with higher contributions are coloured red shades and the ranges with lower contributions are coloured blue shades. As the contribution to the treatment effect may differ from the contribution to the regression coefficient, 2 colours must be displayed for the same covariate range; consequently, the area between the upper interval and the NMR regression line displays the colour shades for the treatment effect, and the area between the lower interval and the NMR regression line displays the colour shades for the coefficient.

The heat‐NMR plot display whether treatment by covariate interactions exist, areas of interpolation or extrapolation, and which covariate ranges contribute most to each NMR estimate.

#### 5.13.2 Application to datasets

### 5.14 Malaria dataset

**Figure 10:**
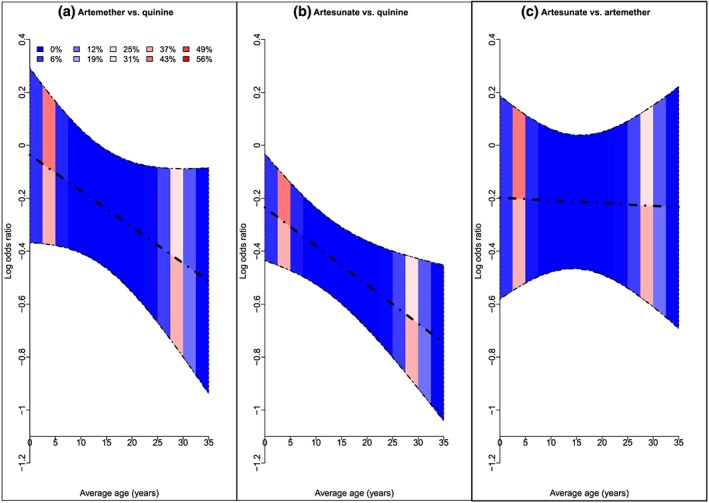
Heat‐NMR plot showing average age versus log odds ratio for the malaria dataset. The bold dot‐dash line is the log odds ratio, and the 2 dashed lines are the upper and lower 95% confidence intervals estimated by the model. Block colours represent the contribution of each covariate range to each log odds ratio and each regression coefficient (%) [Colour figure can be viewed at http://wileyonlinelibrary.com]

The heat‐NMR plot is shown in Figure 10. For each comparison, most of the contributing trials are within range 2.5 to 5 years and 25 to 32.5 years so we can be most confident in interpreting the NMR results for patients within these average age ranges.

### 5.15 Fluoride dataset

**Figure 11:**
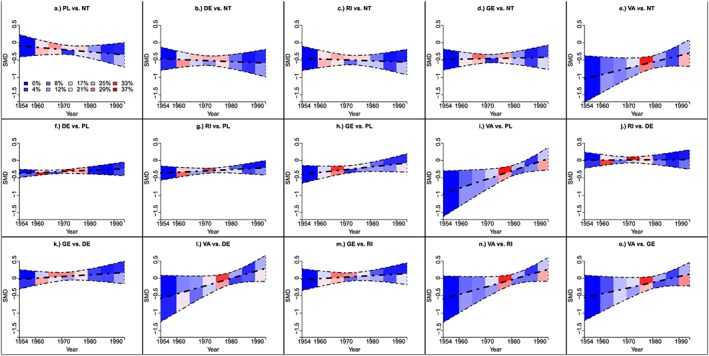
Heat‐NMR plot showing randomisation year versus SMD for the fluoride dataset.The bold dot‐dash line is the SMD, and the 2 dashed lines are the upper and lower 95% confidence intervals estimated by the model. Block colours represent the contribution of each covariate range to each SMD and each regression coefficient (%)DE: dentifrice; GE: gel; NT: no treatment; PL: placebo; RI: rinse; SMD: standardised mean difference; VA: varnish [Colour figure can be viewed at http://wileyonlinelibrary.com]

Figure 11 shows the heat NMR‐plot. For the majority of comparisons, we can be most confident in the NMR results between 1960 to 1980 because most of the contributing trials were randomised within this time period.

## 6 DISCUSSION

The proposed methods can help one to understand which trials and covariate values contribute to each NMR result and highlight extrapolation or interpolation. For both example datasets, we found that the contribution that each trial made to each NMR result varied. For the malaria dataset, for every NMR result, no contributing trials had an average age between 7 and 25 years; thus, results were interpolated. For the fluoride dataset, there were no contributing trials randomised between 1954 and 1959 for most NMR results and no contributing trials randomised between 1985 and 1989 for some NMR results; therefore, within these ranges, these results would be extrapolated. However, we anticipate that in other datasets, the extrapolated or interpolated range may differ more strongly across comparisons, for example, extrapolation within covariate range 7 to 25 years for treatment *3* vs treatment *2* but extrapolation within covariate range 25 to 40 years for treatment *3* vs treatment *1*.

It may be argued that if one truly believes that the model is appropriate and its underlying assumptions are valid, then extrapolated and interpolated results should be of no concern. Yet, we believe that such occasions are likely to be rare because often in an aggregate data NMR, it is difficult to detect whether the consistency assumptions are feasible because of data limitations. Furthermore, if inconsistency is present for a particular treatment comparison in the NMR, it is likely to be caused by differences in the distribution of a covariate from trials that contribute direct evidence and those that contribute indirect evidence. The proposed methods, in particular the contribution‐NMR plot, can help to visualise such differences and therefore understand causes of the inconsistency.

In this article, we have provided new methods to calculate contributions based on models with independent, exchangeable, or common interactions. We applied models including independent interactions, but in some scenarios, this model cannot be fitted, and modellers may apply models with exchangeable or common interactions instead. For instance, when all the trials that contribute to the estimation of one of the coefficients have the same covariate value, which can be problem especially when categorical covariates are considered. In these cases, the exchangeable or common model could be fitted providing that studies that contribute to the results for other comparisons have different categories. For example, when exploring an interaction between treatment effect and study location (ie, continent), studies that contribute to results for comparison *2* vs *1* may all be carried out on the same continent provided that studies that contribute to comparison *3* vs *1* were located on different continents. This means that NMR results can be obtained for a comparison even though there is no direct or indirect evidence for the regression coefficient for that comparison, and hence results on that comparison are not evidence based.

If data are limited, an alternative to using models with exchangeable or common interactions would be to use informative prior distributions in a Bayesian framework. Methods have been presented in this article that can be used with such prior distributions allowing the contribution that the prior distribution makes to each NMR result to be calculated as well as the study contributions. In these situations, informative prior distributions would ideally be evidence based, perhaps elicited from other similar meta‐analyses or expert opinion.

As with all meta‐regression methods, there may be missing covariate data or covariate data may be reported using different statistical summarises across trials meaning that it is not possible to combine all trials. In such situations, ideally the data should be sought from the original trial investigators. If contact with investigators is not fruitful, it may be possible to impute covariate data, the relevant studies could be deleted from the dataset, and/or sensitivity analyses could be carried out to explore the impact of the missing or imputed data on the results.

In this article, we introduced methods to calculate the contribution that each study makes to each NMR result and compare the methods with the existing methods described in Riley et al for calculating study weights in multi‐parameter meta‐analysis than can also be applied to NMR.^23^ When both methods were applied to real data, we found differences in the estimated contributions. Differences may exist for the fluoride dataset, because, when multi‐arm trials exist, the methods proposed by Riley et al estimate the contribution that the study makes to each NMR result, whereas the new methods estimate the contribution that each data point (ie, each observed treatment effect for that study) makes to each NMR result.^23^ Furthermore, the results of the NMR estimated using the Frequentist methods of Riley et al differed from those obtained using Bayesian methods.^23^ Also, it is worth emphasising that the previously proposed methods on which our methods are based have been criticised because the estimated contributions are not invariant to transformations of the data, such that if the data are rescaled, the contributions matrix changes^19^, ^20^, ^21^; whereas the methods proposed by Riley et al produce the same contributions even when the data are transformed.^22^, ^23^ Further work may adapt the methods of Riley et al to accommodate NMR models with exchangeable or common interactions and perhaps prior information in a Bayesian setting, so that the methods can be applied in all situations.^23^

As an alternative method to calculating study contributions, we could have used more similar methods to those proposed elsewhere by fitting a standard pairwise meta‐regression to each comparison in the treatment network to estimate a treatment effect and regression coefficient for each comparison, and then calculating the contribution that each of the pair‐wise treatment effects and coefficients makes to each NMR result.^19^, ^20^, ^21^ Therefore, unlike the methods proposed in this article, the alternative method would not provide the contribution of each trial to each NMR result. The alternative approach could not be used when only 1 trial contributed direct evidence to 1 or more comparisons because a regression coefficient could not be estimated for that comparison, whereas the methods proposed in this article may be used. Also, the conclusions drawn regarding extrapolation and interpolation would be less intuitive; for example, the alternative method would allow one to state that a particular NMR result was based on pairwise estimates that were estimated using trials within range X‐Y; yet, the proposed methods can tell one how much each covariate value contributed to an NMR result.

In this paper, we calculated contributions by modelling trial‐level aggregate data (ie, treatment effects and variances). Individual patient data models can be advantageous over aggregate data models when studying patient‐level covariates because they avoid ecological biases.^39^, ^40^ Yet, it is common to explore patient‐level covariates (eg, patient age) using study‐level covariate summaries (eg, average age of patients) in meta‐regression such as in the malaria dataset. In these instances, the treatment effect is estimated from all patients with potentially widely variable covariate values, yet the full covariate distribution for each study is ignored in the meta‐regression. The methods presented in this article do not show how the “ignored” covariate values contribute to the NMR results. Therefore, the methods presented in this article are limited when patient‐level covariates are of interest, in the same way that existing meta‐regression methods are also limited in such circumstances. Extension of the new methods to individual patient data and other types of aggregate data, such as event rates, and are not straightforward because iteratively weighted least squares estimation with transformed observations is required.

Standard pairwise meta‐regression is a special case of NMR, and so the methods presented here can also be applied to aid analysts' interpretation of pair‐wise meta‐regression by providing a better understanding of the covariate distribution of trials. In principle the proposed methods can be applied for any number of treatments without adaptation; but of course, the number of plots increases with the number of treatments. The methods also apply when multiple covariates are included in the NMR simultaneously. With multiple covariates, the network covariate distribution diagram would display multiple histograms on each edge; therefore, a 3D figure may be favourable (or the histograms can be presented in 2D separate to the network diagram); the covariate‐contribution plot would include graphs showing, for each comparison, the contributions to the treatment effect and the contributions to each regression coefficient; 1 heat plot for each covariate would be constructed; and the contribution‐NMR plot and heat‐NMR plot would include 1 graph for each covariate for each comparison. The methods in this article can also be applied to categorical covariates, in NMR or pairwise meta‐regression and the interpretation is natural; graphs were also constructed using a dataset with a categorical covariate (graphs not presented).

In conclusion, it is important to consider the contribution of trials and covariate values to model estimates in NMR. Graphically displaying the contributions helps to better understand the data, model, and results, and prevent results from being misinterpreted by review users and reviewers.

### CONTRIBUTIONS OF AUTHORS

SDo, SDi, and NW discussed issues around extrapolation in NMR. SDo wrote the manuscript and carried out the analysis. SDi, CTS, and NW provided statistical guidance and commented on the manuscript. VM provided clinical advice on the use and interpretation of the fluoride dataset.

## FUNDING

This research was supported by the Medical Research Council (grant number MR/K021435/1) as part of a career development award in biostatistics awarded to SDo.

## CONFLICTS OF INTEREST

None declared.

## Supporting information

Table S1: Malaria dataset.
Table S2: Fluoride dataset.
Table S3: Results from the fixed‐effect model including independent treatment by covariate interactions for the malaria dataset.
Table S4: Results from the random‐effects model including independent treatment by covariate interactions estimated using Winbugs (Bayesian approach) for the fluoride dataset.
Table S5: Percentage contribution of each trial to each log odds ratio and coefficient using the new methods for the malaria dataset.
Table S6: Study weight of each trial to each log odds ratio and coefficient using the existing methods proposed by Riley et al for the malaria dataset
Table S7: Percentage contribution of each trial to each SMD and coefficient using the new methods for the fluoride dataset.
Table S8: Results from the random‐effects model including independent treatment by covariate interactions estimated using Stata (frequentist approach) for the fluoride dataset.
Table S9: Percentage contribution of each trial to each SMD and coefficient using the existing methods proposed by Riley et al for the fluoride dataset.
Figure S1: NMR plot for the malaria dataset.
Figure S2: NMR plot for the fluoride dataset.
