# Supplementary material for: Graphs of study contributions and covariate distributions for network meta‐regression
Source: Res Synth Methods. 2018 Feb 14;9(2):243–60. doi: 10.1002/jrsm.1292 (PMC6001528; doi:10.1002/jrsm.1292)
Supplement: Supplementary file 1 — Table S1: Malaria dataset. Table S2: Fluoride dataset. Table S3: Results from the fixed‐effect model including independent treatment by covariate interactions for the malaria dataset. Table S4: Results from the random‐effects model including independent treatment by covariate interactions estimated using Winbugs (Bayesian approach) for the fluoride dataset. Table S5: Percentage contribution of each trial to each log odds ratio and coefficient using the new methods for the malaria dataset. Table S6: Study weight of each trial to each log odds ratio and coefficient using the existing methods proposed by Riley et al for the malaria dataset Table S7: Percentage contribution of each trial to each SMD and coefficient using the new methods for the fluoride dataset. Table S8: Results from the random‐effects model including independent treatment by covariate interactions estimated using Stata (frequentist approach) for the fluoride dataset. Table S9: Percentage contribution of each trial to each SMD and coefficient using the existing methods proposed by Riley et al for the fluoride dataset. Figure S1: NMR plot for the malaria dataset. Figure S2: NMR plot for the fluoride dataset. [file JRSM-9-243-s001.zip › Supplementary_material(updated).pdf]

Supplementary material for 'Graphs of study contributions and covariate distributions for network meta-regression.'

Supplementary methods including network meta-regression model specification and derivation of study contributions.

NMR model specification in matrix form

To set notation, suppose there are T treatments in a network (treatments *1, 2, 3,… T*) with treatment *1* as the reference treatment, and a single covariate. For ease of exposition we present example vectors and matrices supposing a three treatment network, however, note that the method is general for any number of treatments and covariates. Initially, we assume that no multi-arm trials are included in the dataset but later we explain how the methods can accommodate them.

Let *i* denote the trial where $i = 1, \dots \dots, N$ and *N* is the number of independent trials. Suppose y_i is the treatment effect estimated in trial *i* (e.g. log odds ratio) and v_i is the variance. Let c_i be a study-level covariate for trial *i* (such as, a continuous covariate value or an indicator variable for a dichotomous covariate).

Fixed-effect NMR model

The fixed-effect NMR model can be written as

$$\boldsymbol{y} \sim N(\boldsymbol{X\mu}, \boldsymbol{V}) \qquad \textbf{(1)}$$

where $\boldsymbol{y}$ is an *N*-length vector of the observed treatment effects, y_i, such that $\boldsymbol{y} = (y_1, y_2, \dots y_N)'$ that follows a multivariate normal distribution with mean $\boldsymbol{X\mu}$ and variance $\boldsymbol{V}$.

Matrix $\boldsymbol{V}$ is a known *N x N* diagonal matrix of variances v_i when each trial estimates only one treatment effect. Here, $\boldsymbol{\mu}$ is a *2(T-1)* vector that represents the basic parameters that are estimated by the model, that is $\boldsymbol{\mu} = (d_{12}, d_{13}, \ldots., d_{1T}, \ \beta_{12}, \beta_{13}, \ldots, \beta_{1T})'$. As an example, for a three treatment network, $\boldsymbol{\mu} = (d_{12}, d_{13}, \beta_{12}, \beta_{13})'$. The basic parameters in a NMR model consist of a treatment effect for each treatment versus the reference treatment *1* when the covariate value is zero (or when the covariate value is the mean when the model is centred) (i.e. $d_{12}, d_{13}, \ldots., d_{1T}$), where for example, d_{12} is the treatment effect of treatment *2* versus treatment *1* at zero covariate, and a regression coefficient for the treatment by covariate interaction for each treatment versus the reference treatment *1* (i.e. $\beta_{12}, \beta_{13}, \ldots, \beta_{1T}$). Matrix $\boldsymbol{X}$ is the *N x 2(T-1)* design matrix that maps the observed treatment effects onto the basic parameters. For example, for a three treatment network, if the first study compared *2* vs. *1*, the second study compared *3* vs. *1*, the third study compared *3* vs. *2* and the last study compared *3* vs. *1*, the design matrix $\boldsymbol{X}$, would be

$$\boldsymbol{X} = \begin{pmatrix} 1 & 0 & c_1 & 0 \\ 0 & 1 & 0 & c_2 \\ -1 & 1 & -c_3 & c_3 \\ \vdots & \vdots & \vdots & \vdots \\ 0 & 1 & 0 & c_N \end{pmatrix}.$$

The design matrix uses the consistency equations that underlie NMR models.[1,2] For instance, the consistency equations are $d_{23} = d_{13} - d_{12}$ for the treatment effects and $\beta_{23} = \beta_{13} - \beta_{12}$ for the regression coefficients for a three treatment network.

The parameters of interest, that is, the treatment effects and regression coefficients for all comparisons, are represented by a *T(T-1)* length vector $\boldsymbol{\theta}$. For example, for a three treatment

network, $\boldsymbol{\theta} = (d_{12}, d_{13}, d_{23}, \beta_{12}, \beta_{13}, \beta_{23})'$. The parameters of interest $\boldsymbol{\theta}$ are linear combinations of the basic parameters $\boldsymbol{\mu}$ such that

$$\boldsymbol{\theta} = \boldsymbol{Z}\,\boldsymbol{\mu} \qquad \textbf{(2)}$$

Where $\boldsymbol{Z}$ is a *T(T-1) x 2(T-1)* matrix that represents the consistency equations, for example, for a three treatment network,

$$\boldsymbol{Z} = \begin{pmatrix} 1 & 0 & 0 & 0 \\ 0 & 1 & 0 & 0 \\ -1 & 1 & 0 & 0 \\ 0 & 0 & 1 & 0 \\ 0 & 0 & 0 & 1 \\ 0 & 0 & -1 & 1 \end{pmatrix}.$$

Random-effects NMR model

The random-effects NMR model can be written as

$$\boldsymbol{y} \sim N(\boldsymbol{\delta}, \boldsymbol{V}) \qquad \textbf{(3)}$$
$$\boldsymbol{\delta} \sim N(\boldsymbol{X\mu}, \boldsymbol{\Lambda_\tau})$$

Here $\boldsymbol{\delta}$ is an N-length vector of trial-specific treatment effects, the *N x N* matrix $\boldsymbol{\Lambda_\tau}$ is a diagonal matrix with the appropriate between trial variances τ^2 along the diagonals. Often in network meta-analysis literature and applications, the between trial variances are assumed to be the same for each comparison to aid estimation.

Multi-arm trials

The models described can also be applied to datasets including multi-arm trials with some minor adaptations. For multi-arm studies there would be more than one y_i per study (e.g. for a three-arm trial there would be two treatment effects y_1 and y_2) and $\boldsymbol{V}$ must include relevant covariances, for example, if the first trial is a three-arm trial,

$$\boldsymbol{V} = \begin{pmatrix} v_1 & cov_{12} & 0 & \cdots & 0 \\ cov_{12} & v_2 & 0 & \cdots & 0 \\ 0 & 0 & v_3 & \cdots & 0 \\ \vdots & \vdots & \vdots & \vdots & \vdots \\ 0 & 0 & 0 & 0 & v_n \end{pmatrix}.$$

Similarly, for random-effects models the trial-specific treatment effects δ_i from the same study are correlated and $\boldsymbol{\Lambda_\tau}$ must include relevant covariance's, for example, if the first trial is a three-arm trial,

$$\boldsymbol{\Lambda_\tau} = \begin{pmatrix} \tau^2 & \tau^2/2 & 0 & \cdots & 0 \\ \tau^2/2 & \tau^2 & 0 & \cdots & 0 \\ 0 & 0 & \tau^2 & \cdots & 0 \\ \vdots & \vdots & \vdots & \vdots & \vdots \\ 0 & 0 & 0 & 0 & \tau^2 \end{pmatrix}.$$

Calculating the trials' contributions using the new methods

We now explain how the trial's contributions can be calculated using a Frequentist framework and then Bayesian methodology. Furthermore, the use of different modelling assumptions that can be made for the regression coefficients are also explained.

Calculating contributions using a Frequentist approach

Fixed-effect model

The basic parameters, $\boldsymbol{\mu}$, can be estimated using the weighted least squares estimate[3,4]

$$\widehat{\boldsymbol{\mu}} = (\mathbf{X}'\boldsymbol{V}^{-1}\boldsymbol{X})^{-1}\boldsymbol{X}'\boldsymbol{V}^{-1}\boldsymbol{y} \quad \textbf{(4)}$$

Substituting equation (4) into equation (2) gives

$$\widehat{\boldsymbol{\theta}} = \boldsymbol{Z}\,(\mathbf{X}'\boldsymbol{V}^{-1}\boldsymbol{X})^{-1}\boldsymbol{X}'\boldsymbol{V}^{-1}\boldsymbol{y}$$

The matrix $\boldsymbol{Z}(\mathbf{X}'\boldsymbol{V}^{-1}\boldsymbol{X})^{-1}\boldsymbol{X}'\boldsymbol{V}^{-1}$ is a *T(T-1) x N* matrix that maps the observed treatment effects $\boldsymbol{y}$ onto the parameters of interest $\widehat{\boldsymbol{\theta}}$. Consequently, the matrix describes the contribution of each trial's result on each NMR result (i.e. the parameters of interest). To calculate the percentage contribution that each trial makes to each NMR result, absolute values of the matrix entries are found, then the absolute values of entries in each row are summed, then the percentage contribution for each matrix entry is calculated as the absolute value of the entry divided by the summed total for its row multiplied by 100% (Krahn et al., 2013). This allows one to know which trials (and therefore covariate values) contribute to each NMR estimate and by how much. In other words, for each NMR result (e.g. treatment effect for *2* vs *1*), the amount that each trial contributes to the result is estimated; the contributions across all trials sum to 100%.

Random-effects model

To calculate contributions, one must assume the matrix $\boldsymbol{\Lambda}_{\boldsymbol{\tau}}$ is known and fixed (when in fact it is unknown). The between trial-variances, and therefore, $\boldsymbol{\Lambda}_{\boldsymbol{\tau}}$ can be estimated by fitting the NMR model and $\boldsymbol{\Lambda}_{\boldsymbol{\tau}}$ is then assumed to be known in the calculation of the contributions such that uncertainty in $\boldsymbol{\Lambda}_{\boldsymbol{\tau}}$ is ignored.

Assuming the variance matrices are known allows the hierarchical model to be rewritten as a single normal regression model[5] such that

$$\boldsymbol{y}_* \sim N(\boldsymbol{X}_*\boldsymbol{\mu}_*, \boldsymbol{V}_*)$$

where $\boldsymbol{y}_* = (\boldsymbol{y}, \boldsymbol{0})'$ of length *2N*, $\boldsymbol{\mu}_* = (\boldsymbol{\delta}, \boldsymbol{\mu})'$ of length *N+2(T-1),*

$$\boldsymbol{X}_* = \begin{pmatrix} \boldsymbol{I_N} & \boldsymbol{0} \\ \boldsymbol{I_N} & -\mathbf{X} \end{pmatrix},$$

and

$$\boldsymbol{V}_* = \begin{pmatrix} \boldsymbol{V} & \boldsymbol{0} \\ \boldsymbol{0} & \boldsymbol{\Lambda_\tau} \end{pmatrix}.$$

Then the estimate $\widehat{\boldsymbol{\mu}_*}$can be obtained using weighted least squares regression, such that

$$\widehat{\boldsymbol{\mu}_*} = (\boldsymbol{X}_*'\boldsymbol{V}_*^{-1}\boldsymbol{X}_*)^{-1}\boldsymbol{X}_*'\boldsymbol{V}_*^{-1}\boldsymbol{y}_* \quad \textbf{(5)}$$

In this case, let $\boldsymbol{A}$ denote the matrix $(\boldsymbol{X}_*'\boldsymbol{V}_*^{-1}\boldsymbol{X}_*)^{-1}\boldsymbol{X}_*'\boldsymbol{V}_*^{-1}$**,** which is a *N+T(T-1) x 2N* matrix. Since $\widehat{\boldsymbol{\mu}_*} = \boldsymbol{A}\boldsymbol{y}_*$, we can write

$$\begin{pmatrix} \widehat{\boldsymbol{\delta}} \\ \widehat{\boldsymbol{\mu}} \end{pmatrix} = \boldsymbol{A} \begin{pmatrix} \boldsymbol{y} \\ \boldsymbol{0} \end{pmatrix}$$

From this we can see that $\widehat{\boldsymbol{\mu}}$ is estimated by a section of the matrix $\boldsymbol{A}$ (that is the last *2(T-1)* rows of the first *N* columns) multiplied by $\boldsymbol{y}$. By equation (2) multiplying $\boldsymbol{Z}$ by the section of matrix $\boldsymbol{A}$ provides the required matrix that maps the observed treatment effects $\boldsymbol{y}$ onto the parameters of interest $\widehat{\boldsymbol{\theta}}$ and the contributions can be calculated as previously described.

Calculating contributions using a Bayesian approach

Fixed-effect model

We assume a Normal prior distribution for the unknown basic parameters in the fixed-effect NMR model (equation 1), that is,

$$\boldsymbol{\mu}|\boldsymbol{\mu_0} \sim N(\boldsymbol{\mu_0}, \boldsymbol{\Lambda_0}) \qquad \textbf{(6)}$$

For instance, for a non-informative prior distribution, $\boldsymbol{\mu_0} = (0, 0, \ldots, 0)'$ is a *2(T-1)* length vector and $\boldsymbol{\Lambda_0}$ is a *2(T-1) x 2(T-1)* diagonal matrix with large variances along the diagonals.

Gelman *et al*[6] (page 392) explains that in this situation a multivariate Normal prior distribution can be treated as additional data points such that

$$\boldsymbol{y}_* \sim N(\boldsymbol{X}_*\boldsymbol{\mu},\ \boldsymbol{V}_*)$$

where $\boldsymbol{y}_* = (\boldsymbol{y}, \boldsymbol{\mu_0})'$, $\boldsymbol{X}_* = (\boldsymbol{X}, \boldsymbol{I_{2(T-1)}})'$ where $\boldsymbol{I_{2(T-1)}}$ is a *2(T-1) x 2(T-1)* identity matrix, and $\boldsymbol{V}_* = \begin{pmatrix} \boldsymbol{V} & \boldsymbol{0} \\ \boldsymbol{0} & \boldsymbol{\Lambda_0} \end{pmatrix}$**.** Then the estimate $\hat{\boldsymbol{\mu}}$ can be obtained using the weighted least squares estimate (equation 5).

Here, the matrix $\boldsymbol{Z}(\boldsymbol{X}_*'\boldsymbol{V}_*^{-1}\boldsymbol{X}_*)^{-1}\boldsymbol{X}_*'\boldsymbol{V}_*^{-1}$ is a *T(T-1) x N+2(T-1)* matrix that maps the $\boldsymbol{y}_*$ onto the parameters of interest $\hat{\boldsymbol{\theta}}$. This matrix enables one to estimate the contribution of prior distributions as well as the contribution that each study makes to each NMR result. However, when non-informative prior distributions are used the contribution of the priors will be negligible.

To ignore the contributions from the prior distributions, an appropriate section of the matrix is taken. Let A denote the matrix $(\boldsymbol{X}_*'\boldsymbol{V}_*^{-1}\boldsymbol{X}_*)^{-1}\boldsymbol{X}_*'\boldsymbol{V}_*^{-1}$**,** which is a 2*(T-1) x N*+ 2*(T-1)* matrix in this case. Since $\widehat{\boldsymbol{\mu}} = \boldsymbol{A}\boldsymbol{y}_*$, we can write

$$\widehat{\boldsymbol{\mu}} = \boldsymbol{A}\begin{pmatrix}\boldsymbol{y}\\ \boldsymbol{\mu_0}\end{pmatrix}$$

When $\boldsymbol{\mu_0} = (0, 0, \ldots ,0)'$, $\widehat{\boldsymbol{\mu}}$ is estimated by a section of the matrix $\boldsymbol{A}$ **(**that is the first *N* columns) multiplied by $\boldsymbol{y}$. By equation (2) multiplying $\boldsymbol{Z}$ by the section of matrix $\boldsymbol{A}$ provides the appropriate matrix that maps the observed treatment effects $\boldsymbol{y}$ onto the parameters of interest $\widehat{\boldsymbol{\theta}}$. This matrix enables one to estimate the contribution that each study makes to each NMR result, ignoring contributions of the prior distributions.

In any case, if non-informative prior distributions are used with infinite variances then the estimation is the same as for frequentist methods.[6]

Random-effects model

For the random-effects NMR model (equation 3), we assume a Normal prior distribution for the unknown basic parameters (equation 6).

We can find an approximate Bayesian posterior solution by assuming that the matrix $\boldsymbol{\Lambda_\tau}$ is known and fixed[6] (page 408), by writing the hierarchical model as

$$\boldsymbol{y}_* \sim N(\boldsymbol{X}_*\boldsymbol{\mu}_*,\ \boldsymbol{V}_*)$$

where $\boldsymbol{y}_* = (\boldsymbol{y}, \boldsymbol{0}, \boldsymbol{\mu_0})'$ of length *2N+2(T-1),* $\boldsymbol{\mu}_* = (\boldsymbol{\delta}, \boldsymbol{\mu})'$ of length *N+2(T-1),*

$$\boldsymbol{X}_* = \begin{pmatrix} \boldsymbol{I}_N & \boldsymbol{0} \\ \boldsymbol{I}_N & -\mathbf{X} \\ \boldsymbol{0} & \boldsymbol{I}_{2(T-1)} \end{pmatrix}$$

and

$$\boldsymbol{V}_* = \begin{pmatrix} \boldsymbol{V} & \boldsymbol{0} & \boldsymbol{0} \\ \boldsymbol{0} & \boldsymbol{\Lambda}_\tau & \boldsymbol{0} \\ \boldsymbol{0} & \boldsymbol{0} & \boldsymbol{\Lambda}_0 \end{pmatrix}.$$

The estimate $\widehat{\boldsymbol{\mu}}$ can be obtained using the weighted least squares estimate (equation 5).

In this case, let $\boldsymbol{A}$ denote the matrix $(\boldsymbol{X}_*'\boldsymbol{V}_*^{-1}\boldsymbol{X}_*)^{-1}\boldsymbol{X}_*'\boldsymbol{V}_*^{-1}$**,** which is a *N+2(T-1) x 2N+2(T-1)* matrix. Since $\widehat{\boldsymbol{\mu}_*} = \boldsymbol{A}\boldsymbol{y}_*$, we can write

$$\begin{pmatrix} \widehat{\boldsymbol{\delta}} \\ \widehat{\boldsymbol{\mu}} \end{pmatrix} = \boldsymbol{A}\begin{pmatrix} \boldsymbol{y} \\ \boldsymbol{0} \\ \boldsymbol{\mu}_0 \end{pmatrix}$$

When $\boldsymbol{\mu}_0 = (0, 0, \ldots, 0)'$, $\widehat{\boldsymbol{\mu}}$ is estimated by a section of the matrix $\boldsymbol{A}$ **(**that is the last *2(T-1)* rows of the first *N* columns) multiplied by $\boldsymbol{y}$. By equation (2) multiplying $\boldsymbol{Z}$ by the section of matrix $\boldsymbol{A}$ provides the required matrix that maps the observed treatment effects $\boldsymbol{y}$ onto the parameters of interest $\widehat{\boldsymbol{\theta}}$ and the contributions can be calculated as previously described. Contributions made by prior distributions are ignored when taking the specified section of the matrix but may be calculated using a different matrix section. Similarly, when using non-informative prior distributions are used with infinite variances, the estimation is the same as for frequentist methods.[6]

Assumptions on the regression coefficients for the treatment by covariate interactions

So far, the presented methods and models have assumed the regression coefficients for the basic regression coefficients are independent. However, the methods can equally be applied while assuming common or exchangeable basic regression coefficients.

When the basic regression coefficients (i.e. $\beta_{12}, \beta_{13}, \dots, \beta_{1T}$) are assumed to be common, we can apply the same methods as described previously but we now set $\beta = \beta_{12} = \beta_{13} = \cdots = \beta_{1T}$, for example, for a three treatment network, $\boldsymbol{\mu} = (d_{12}, d_{13}, \beta)'$, $\boldsymbol{\theta} = (d_{12}, d_{13}, d_{23}, \beta)'$ and

$$\boldsymbol{Z} = \begin{pmatrix} 1 & 0 & 0 \\ 0 & 1 & 0 \\ -1 & 1 & 0 \\ 0 & 0 & 1 \end{pmatrix}.$$

If the basic regression coefficients (i.e. $\beta_{12}, \beta_{13}, \dots, \beta_{1T}$) are assumed to be exchangeable, the corresponding NMR model is a hierarchical model and can be written as a single linear regression so that weighted least squares estimation can be used. For example, the random-effects Bayesian model would be

$$\boldsymbol{y}|\boldsymbol{\delta} \sim N(\boldsymbol{\delta}, \boldsymbol{V})$$

$$\boldsymbol{\delta}|\boldsymbol{\mu} \sim N(\boldsymbol{X\mu}, \boldsymbol{\Lambda_\tau})$$

$$\boldsymbol{\mu}|B \sim N(\boldsymbol{m}B, \boldsymbol{\Lambda_B})$$

where $\boldsymbol{m}$ is a *2(T-1)* length vector, for example for a three treatment network $\boldsymbol{m} = (\mu_{01}, \mu_{02}, 1, 1)'$ where μ_{01} and μ_{02} are the means of the prior distributions for d_{12} and d_{13}. Matrix $\boldsymbol{\Lambda_B}$ is a *2(T-1) x 2(T-1)* matrix, for example,

$$\boldsymbol{\Lambda_B}=\begin{pmatrix} \upsilon_{01} & 0 & 0 & 0 \\ 0 & \upsilon_{02} & 0 & 0 \\ 0 & 0 & \tau_\beta^2 & 0 \\ 0 & 0 & 0 & \tau_\beta^2 \end{pmatrix}$$

where υ_{01} and υ_{02} are the variances of the prior distributions for d_{12} and d_{13}. We also assume a Normal prior distribution for the unknown parameter B such that $B|B_0 \sim N(B_0, \omega_0)$.

Assuming the variances matrices are known, the hierarchical model can be written as a linear regression model as follows

$$\boldsymbol{y}_* \sim N(\boldsymbol{X}_*\boldsymbol{\mu}_*,\ \boldsymbol{V}_*)$$

where $\boldsymbol{y}_* = (\boldsymbol{y}, \boldsymbol{0}, \boldsymbol{0}, B_0)'$ of length *2N+1+2(T-1)*, $\boldsymbol{\mu}_* = (\boldsymbol{\delta}, \boldsymbol{\mu}, B)'$ of length *N*+2*(T-1)+1*,

$$\boldsymbol{X}_* = \begin{pmatrix} \boldsymbol{I_N} & \boldsymbol{0} & \boldsymbol{0} \\ \boldsymbol{I_N} & -\boldsymbol{X} & \boldsymbol{0} \\ \boldsymbol{0} & \boldsymbol{I_{2(T-1)}} & -\boldsymbol{m} \\ \boldsymbol{0} & 0 & \boldsymbol{I_1} \end{pmatrix}$$

and

$$\boldsymbol{V}_* = \begin{pmatrix} \boldsymbol{V} & \boldsymbol{0} & \boldsymbol{0} & 0 \\ \boldsymbol{0} & \boldsymbol{\Lambda_\tau} & \boldsymbol{0} & 0 \\ \boldsymbol{0} & \boldsymbol{0} & \boldsymbol{\Lambda_B} & 0 \\ 0 & 0 & 0 & \omega_0 \end{pmatrix}.$$

Estimation of $\boldsymbol{\mu}$ and calculation of the contributions is as described previously.

Calculating the trials' contributions using the methods by Riley *et al*[7]

Here we provide a summary of the methods described by Riley *et al.*[7] All parameters are as described for the new methods. Riley *et al* shows that for a fixed-effect NMR model, an estimate of $\boldsymbol{\mu}$ and its variance are given by

$$\hat{\boldsymbol{\mu}} = (\mathbf{X}'\boldsymbol{V}^{-1}\boldsymbol{X})^{-1}\boldsymbol{X}'\boldsymbol{V}^{-1}\boldsymbol{y}$$

and

$$var(\hat{\boldsymbol{\mu}}) = (\mathbf{X}'\boldsymbol{V}^{-1}\boldsymbol{X})^{-1}$$

respectively. Likewise, for a random-effects NMR model, assuming $\boldsymbol{\Lambda_\tau}$ is known and fixed, an estimate of $\boldsymbol{\mu}$ is given by

$$\hat{\boldsymbol{\mu}} = (\mathbf{X}'(\boldsymbol{\Lambda_\tau} + \boldsymbol{V})^{-1}\boldsymbol{X})^{-1}\boldsymbol{X}'(\boldsymbol{\Lambda_\tau} + \boldsymbol{V})^{-1}\boldsymbol{y}$$

and its variance is

$$var(\hat{\boldsymbol{\mu}}) = (\mathbf{X}'(\boldsymbol{\Lambda_\tau} + \boldsymbol{V})^{-1}\boldsymbol{X})^{-1}.$$

To calculate the study weights, firstly the NMR is fitted to obtain estimates of $var(\hat{\boldsymbol{\mu}})$. The inverse of $var(\hat{\boldsymbol{\mu}})$ gives Fishers total information matrix denoted as $\boldsymbol{I}_{total}(\hat{\boldsymbol{\mu}})$.

Fisher's total information matrix can be decomposed into a sum of independent matrices for each study, that is $\boldsymbol{I}_i(\hat{\boldsymbol{\mu}})$. The second step is to calculate the information matrix for each study *i*; that is $\boldsymbol{I}_i(\hat{\boldsymbol{\mu}}) = \mathbf{X}'\boldsymbol{V}_{\nabla i}^{-1}\boldsymbol{X}$ for a fixed-effect model where $\boldsymbol{V}_{\nabla i}^{-1}$is the same as $\boldsymbol{V}^{-1}$ except all diagonal elements not corresponding with study i are replaced with large numbers (e.g.

100000000000) and all off-diagonal elements not corresponding with study i are replaced with zeros. For a random-effects model, $\boldsymbol{V}_{\nabla i}^{-1}$ is replaced by $(\boldsymbol{\Lambda}_{\boldsymbol{\tau}} + \mathbf{V})_{\nabla i}^{-1}$ in the formula for $\boldsymbol{I}_i(\widehat{\boldsymbol{\mu}})$.

Thirdly, the weight matrix, $\boldsymbol{W}_i(\widehat{\boldsymbol{\mu}})$, is obtained for each study i using the formula $\boldsymbol{W}_i(\widehat{\boldsymbol{\mu}}) = var(\widehat{\boldsymbol{\mu}}) x\ \boldsymbol{I}_i(\widehat{\boldsymbol{\mu}})\ x\ var(\widehat{\boldsymbol{\mu}})$ where x represents matrix multiplication.

Lastly, the percentage study weight for the parameter corresponding with row r of $\widehat{\boldsymbol{\mu}}$ is given by

$$100\%\ x\ \frac{\boldsymbol{W}_i(\widehat{\boldsymbol{\mu}})_{r,r}}{var(\widehat{\boldsymbol{\mu}})_{r,r}}$$

Where *r,r* refers to the element in row *r* and column *r* of the matrix.

References

1. Donegan S, Williamson P, D'Alessandro U, Tudur Smith C. Assessing the consistency assumption by exploring treatment by covariate interactions in mixed treatment comparison meta-analysis: individual patient-level covariates versus aggregate trial-level covariates. *Stat Med.* 2012;31(29):3840-3857.
2. Lu G, Ades A. Assessing evidence inconsistency in mixed treatment comparisons. *J Am Stat Assoc* 2006;101(474):447-459.
3. Salanti G, Del Giovane C, Chaimani A, Caldwell DM, Higgins JPT. Evaluating the Quality of Evidence from a Network Meta-Analysis. *PLoS ONE.* 2014;9(7):e99682.
4. Whitehead A. *Meta-Analysis of Controlled Clinical Trials.* 2002; Chichester: Wiley.
5. Goldstein H. Multilevel mixed linear model analysis using iterative generalized least squares. *Biometrika.* 1986;73(1):43-56.
6. Gelman A, Carlin JB, Stern HS, Rubin DB. *Bayesian Data Analysis.* 2009. CHAPMAN & HALL/CRC.
7. Riley RD, Ensor J, Jackson D, Burke DL. Deriving percentage study weights in multi-parameter meta-analysis models: with application to meta-regression, network meta-analysis and one-stage individual participant data models. *Stat Methods Med Res.* 2017:0962280216688033. doi: 10.1177/0962280216688033.

	Trial	Event rates (number of patients with events/total number of patients)						Log odds ratio	Standard error	Average age (years)
		Quinine		Artemether		Artesunate				
1	**Adam 2002**	1	21	0	20	-	-	-1.10	1.66	3.84
2	**Aguwa 2010**	6	46	7	44	-	-	0.23	0.60	3.50
3	**Hien 1996**	47	276	36	284	-	-	-0.35	0.24	30.00
4	**Huda 2003**	6	23	5	23	-	-	-0.24	0.69	6.20
5	**Karbwang 1992**	5	12	1	14	-	-	-2.23	1.19	31.05
6	**Karbwang 1995**	19	50	6	47	-	-	-1.43	0.53	26.50
7	**Minta 2005**	2	34	4	33	-	-	0.79	0.90	6.80
8	**Murphy 1996**	8	71	18	89	-	-	0.69	0.46	2.33
9	**Ojuawo 1998**	2	19	1	18	-	-	-0.69	1.27	3.93
10	**Olumese 1999**	14	49	11	54	-	-	-0.45	0.46	3.15
11	**Osonuga 2009**	0	16	0	16	-	-	0.00	2.03	7.00
12	**Taylor 1998**	13	81	9	83	-	-	-0.45	0.47	3.08
13	**van Hensbroek 1996**	62	288	59	288	-	-	-0.06	0.20	3.92
14	**Walker 1993**	6	29	3	25	-	-	-0.65	0.77	3.00
15	**Anh 1989**	7	22	-	-	2	19	-1.38	0.88	34.47
16	**Anh 1995**	18	91	-	-	8	99	-1.03	0.45	30.95
17	**Cao 1997**	5	35	-	-	4	37	-0.32	0.72	5.50
18	**Dondorp 2005**	164	731	-	-	107	730	-0.52	0.14	27.90
19	**Dondorp 2010**	297	2713	-	-	230	2712	-0.28	0.09	2.85
20	**Eltahir 2010**	2	33	-	-	1	33	-0.72	1.25	4.50
21	**Hien 1992**	8	30	-	-	5	31	-0.64	0.64	28.50
22	**Newton 2003**	12	54	-	-	7	59	-0.75	0.52	25.00
23	**Phu 2010**	-	-	24	184	13	186	-0.69	0.36	32.25
24	**Vinh 1997**	-	-	5	45	10	79	0.15	0.58	27.33

Table S1: Malaria dataset.

Drugs were coded as quinine=1, artemether=2, and artesunate=3.

	Trial	Mean (standard deviation), total number of patients						SMD	SE	Year
		No treatment	Placebo	Dentifrice	Rinse	Gel	Varnish			
1	**Torell 1965 (2Fagents)**	10.16 (6.59), 333	10.02 (6.75), 162	-	-	-	-	-0.02	0.10	1962
1	**Torell 1965 (2Fagents)**	10.16 (6.59), 333	-	8.09 (6.86), 335	-	-	-	-0.31	0.08	1962
1	**Torell 1965 (2Fagents)**	10.16 (6.59), 333	-	-	6.54 (5.17), 332	-	-	-0.61	0.08	1962
2	**Craig 1981**	2.59 (2.70), 48	-	-	1.77 (2.73), 49	-	-	-0.30	0.20	1977
3	**Moreira 1981**	5.60 (3.60), 91	-	-	4.20 (3.33), 73	-	-	-0.40	0.16	1974
4	**Ruiken 1987(Cluster)**	6.56 (5.34), 78	-	-	4.41 (4.29), 129	-	-	-0.46	0.15	1981
5	**Englander 1971**	2.20 (2.97), 220	-	-	-	1.57 (2.94), 337	-	-0.21	0.09	1967
6	**Ingraham 1970(2trays)**	3.13 (2.70), 63	-	-	-	1.84 (2.24), 56	-	-0.52	0.19	1965
7	**Mestrinho 1983**	4.16 (4.15), 87	-	-	-	3.02 (3.48), 87	-	-0.30	0.15	1981
8	**Englander 1967(2Fagents)**	4.39 (4.47), 195	-	-	-	1.00 (3.84), 305	-	-0.83	0.10	1964
9	**Abadia 1978 (2techniques)**	5.46 (4.82), 90	-	-	-	4.69 (4.44), 164	-	-0.17	0.13	1977
10	**Bryan 1970**	7.26 (4.76), 105	-	-	-	4.56 (4.50), 103	-	-0.58	0.14	1966
11	**Cobb 1980**	8.15 (7.68), 78	-	-	-	5.28 (7.07), 115	-	-0.39	0.15	1977
12	**Horowitz 1971**	8.61 (7.95), 170	-	-	-	6.51 (6.75), 182	-	-0.29	0.11	1965
13	**Bijella 1981**	9.25 (6.45), 160	-	-	-	4.50 (4.34), 160	-	-0.86	0.12	1979
14	**Modeer 1984**	2.00 (2.78), 107	-	-	-	-	1.40 (2.28), 87	-0.23	0.14	1979
15	**Bravo 1997 (cluster)**	2.58 (2.63), 116	-	-	-	-	1.48 (2.20), 98	-0.45	0.14	1990
16	**Holm 1984**	3.15 (1.31), 53	-	-	-	-	1.43 (1.49), 42	-1.23	0.23	1977

	Trial	Mean (standard deviation), total number of patients						SMD	SE	Year
		No treatment	Placebo	Dentifrice	Rinse	Gel	Varnish			
17	**Koch 1975**	4.00 (3.75), 61	-	-	-	-	0.90 (3.80), 60	-0.82	0.19	1973
18	**Ashley 1977**	-	5.61 (4.64), 243	4.44 (4.02), 246	-	-	-	-0.27	0.09	1973
19	**Blinkhorn 1983**	-	6.25 (5.55), 184	4.60 (4.33), 184	-	-	-	-0.33	0.10	1972
20	**Ringelberg 1979 (2Fconc/2agents)**	-	6.25 (7.36), 186	5.13 (5.85), 370	-	-	-	-0.18	0.09	1973
21	**Koch 1967**	-	21.06 (9.56), 202	11.65 (8.18), 124	-	-	-	-1.04	0.12	1962
22	**Marthaler 1970 (Age 2)**	-	3.95 (2.70), 20	2.57 (2.09), 23	-	-	-	-0.58	0.31	1966
23	**Marthaler 1970 (1)**	-	4.40 (3.14), 57	3.44 (2.52), 43	-	-	-	-0.33	0.20	1966
24	**Mainwaring 1978**	-	8.27 (6.62), 316	6.95 (5.96), 791	-	-	-	-0.21	0.07	1974
25	**Kleber 1996**	-	1.59 (2.13), 79	1.66 (2.81), 77	-	-	-	0.03	0.16	1994
26	**Dolles 1980**	-	2.30 (3.00), 23	1.92 (2.71), 24	-	-	-	-0.13	0.29	1974
27	**Muhler 1955**	-	2.42 (3.09), 225	1.55 (2.42), 219	-	-	-	-0.31	0.10	1954
28	**Peterson 1979 (2abras)**	-	3.18 (3.86), 245	2.87 (3.66), 467	-	-	-	-0.08	0.08	1971
29	**Glass 1983 (2abras)**	-	3.21 (4.04), 286	2.39 (3.00), 567	-	-	-	-0.24	0.07	1976
30	**Forsman 1974 (1-city V (2Fconc))**	-	3.25 (3.63), 145	2.92 (3.42), 414	-	-	-	-0.09	0.10	1970
31	**Segal 1967**	-	3.33 (3.68), 310	2.69 (3.27), 338	-	-	-	-0.18	0.08	1964
32	**Kinkel 1972**	-	3.41 (3.72), 345	2.14 (2.88), 354	-	-	-	-0.38	0.08	1969
33	**Reed 1973 (3Fconc)**	-	4.00 (4.18), 397	3.53 (4.21), 1128	-	-	-	-0.11	0.06	1970
34	**Cahen 1982 (2Fagents/2abras)**	-	4.05 (3.46), 708	3.54 (3.18), 1300	-	-	-	-0.16	0.05	1977
35	**Muhler 1970**	-	4.05 (3.99), 235	2.87 (3.47), 201	-	-	-	-0.31	0.10	1967

	Trial	Mean (standard deviation), total number of patients						SMD	SE	Year
		No treatment	Placebo	Dentifrice	Rinse	Gel	Varnish			
36	**Thomas 1966 (2abras)**	-	4.08 (4.11), 155	2.84 (2.92), 309	-	-	-	-0.37	0.10	1961
37	**Reed 1975**	-	4.32 (4.21), 176	3.02 (3.31), 168	-	-	-	-0.34	0.11	1968
38	**Zacherl 1973**	-	5.04 (6.39), 224	3.55 (5.77), 220	-	-	-	-0.24	0.10	1970
39	**James 1967**	-	5.20 (5.42), 397	4.26 (5.19), 406	-	-	-	-0.18	0.07	1962
40	**Lind 1974**	-	5.43 (5.28), 575	3.71 (4.38), 592	-	-	-	-0.36	0.06	1970
41	**Mergele 1968**	-	5.57 (4.30), 190	4.83 (3.28), 197	-	-	-	-0.19	0.10	1964
42	**Held 1968 (site C)**	-	5.60 (4.89), 18	6.10 (5.13), 14	-	-	-	0.10	0.36	1961
43	**Weisenstein 1972**	-	5.60 (4.34), 196	4.98 (4.00), 206	-	-	-	-0.15	0.10	1969
44	**Slack 1967**	-	5.62 (5.62), 340	5.59 (5.34), 356	-	-	-	-0.01	0.08	1963
45	**Forsman 1974 (2-city L (2Fconc))**	-	5.67 (4.93), 132	5.24 (4.72), 262	-	-	-	-0.09	0.11	1970
46	**Horowitz 1966* (=comparison A only)**	-	5.87 (4.75), 309	4.88 (3.81), 329	-	-	-	-0.23	0.08	1961
47	**Slack 1967A**	-	5.95 (5.68), 381	5.64 (4.83), 376	-	-	-	-0.06	0.07	1962
48	**Zacherl 1981(2Fagents/2abras)**	-	6.02 (7.71), 254	4.10 (5.63), 1500	-	-	-	-0.32	0.07	1977
49	**Zacherl 1970* (1)**	-	6.36 (4.69), 261	3.79 (3.49), 251	-	-	-	-0.62	0.09	1963
50	**Koch 1967d**	-	6.37 (5.09), 128	5.67 (4.17), 127	-	-	-	-0.15	0.13	1962
51	**Rule 1984**	-	6.39 (4.69), 416	4.56 (4.72), 460	-	-	-	-0.39	0.07	1977
52	**Murray 1980 (2abrasives)**	-	6.43 (6.02), 356	4.48 (5.25), 750	-	-	-	-0.35	0.06	1974
53	**Gish 1966* (2examiners)**	-	6.44 (5.18), 163	4.75 (4.24), 165	-	-	-	-0.36	0.11	1963

	Trial	Mean (standard deviation), total number of patients						SMD	SE	Year
		No treatment	Placebo	Dentifrice	Rinse	Gel	Varnish			
54	**Zacherl 1972A (4Fagents)**	-	6.62 (6.25), 210	5.08 (5.21), 684	-	-	-	-0.28	0.08	1969
55	**Brudevold 1966* (2Fagents/2abras)**	-	7.03 (6.11), 323	5.56 (6.11), 955	-	-	-	-0.24	0.06	1961
56	**Hargreaves 1973(Age 1)**	-	7.16 (5.60), 140	5.39 (4.79), 163	-	-	-	-0.34	0.12	1968
57	**Hanachowicz 1984**	-	7.23 (5.59), 472	5.30 (4.45), 473	-	-	-	-0.38	0.07	1979
58	**Abrams 1980 (2abras)**	-	7.33 (5.67), 380	6.42 (5.27), 761	-	-	-	-0.17	0.06	1976
59	**Glass 1978**	-	7.36 (7.68), 168	5.31 (5.95), 178	-	-	-	-0.30	0.11	1974
60	**Andlaw 1975**	-	7.68 (5.24), 376	6.07 (4.97), 364	-	-	-	-0.32	0.07	1970
61	**Howat 1978**	-	7.69 (6.39), 242	5.71 (5.49), 253	-	-	-	-0.33	0.09	1974
62	**Marthaler 1965(1)**	-	7.71 (4.70), 124	5.31 (3.75), 145	-	-	-	-0.57	0.12	1958
63	**Hodge 1980 (2Fagents/2abrasives)**	-	7.83 (5.49), 202	6.43 (5.15), 597	-	-	-	-0.27	0.08	1976
64	**Hargreaves 1973(Age 2)**	-	7.97 (5.94), 138	5.71 (4.94), 146	-	-	-	-0.41	0.12	1968
65	**Held 1968 (site B)**	-	8.00 (5.95), 17	5.50 (4.84), 19	-	-	-	-0.46	0.34	1961
66	**Jackson 1967**	-	8.20 (5.45), 433	7.23 (4.70), 438	-	-	-	-0.19	0.07	1962
67	**Fogels 1979 (2abras)**	-	8.34 (6.09), 449	6.83 (5.46), 890	-	-	-	-0.27	0.06	1972
68	**Marthaler 1974**	-	8.39 (5.77), 59	5.62 (5.46), 50	-	-	-	-0.49	0.20	1966
69	**Zacherl 1972**	-	8.39 (6.58), 216	6.51 (5.21), 231	-	-	-	-0.32	0.10	1969
70	**Peterson 1967* (2Fagents/2abras)**	-	9.19 (6.12), 320	7.63 (5.59), 634	-	-	-	-0.27	0.07	1964
71	**Naylor 1967**	-	9.22 (6.20), 479	7.94 (5.09), 494	-	-	-	-0.23	0.06	1961

	Trial	Mean (standard deviation), total number of patients						SMD	SE	Year
		No treatment	Placebo	Dentifrice	Rinse	Gel	Varnish			
72	**Naylor 1979**	-	10.42 (6.47), 306	8.09 (6.09), 319	-	-	-	-0.37	0.08	1973
73	**Koch 1967c**	-	10.66 (7.40), 38	6.56 (4.30), 32	-	-	-	-0.66	0.25	1963
74	**Torell 1965a (age 1)**	-	10.81 (6.20), 137	10.11 (5.11), 148	-	-	-	-0.12	0.12	1962
75	**Mainwaring 1983 (2Fagents)**	-	11.00 (8.23), 224	8.88 (6.44), 458	-	-	-	-0.30	0.08	1978
76	**Di Maggio 1980**	-	11.55 (3.53), 20	4.45 (0.98), 22	-	-	-	-2.80	0.43	1977
77	**James 1977**	-	11.80 (7.37), 379	8.20 (6.03), 403	-	-	-	-0.54	0.07	1970
78	**Torell 1965b (age 2)**	-	12.12 (7.24), 180	10.25 (6.03), 188	-	-	-	-0.28	0.10	1962
79	**Fanning 1968**	-	12.23 (6.37), 422	9.67 (6.37), 422	-	-	-	-0.40	0.07	1964
80	**Slack 1971(2Fagents/2abras)**	-	12.83 (8.31), 289	10.67 (7.68), 821	-	-	-	-0.28	0.07	1965
81	**Held 1968 (site A)**	-	13.20 (7.84), 31	2.60 (3.21), 32	-	-	-	-1.78	0.30	1962
82	**Hargreaves 1973(Age 3)**	-	13.83 (8.04), 151	10.68 (6.98), 146	-	-	-	-0.42	0.12	1968
83	**Zacherl 1970* (2)**	-	15.04 (8.19), 268	8.50 (6.61), 260	-	-	-	-0.88	0.09	1963
84	**Marthaler 1965(2)**	-	15.25 (8.55), 32	11.33 (7.59), 42	-	-	-	-0.49	0.24	1958
85	**Buhe 1984 (2Fconc)**	-	16.60 (9.23), 427	13.01 (7.92), 859	-	-	-	-0.43	0.06	1976
18	**Ashley 1977**	-	5.61 (4.64), 243	-	4.81 (4.21), 245	-	-	-0.18	0.09	1973
19	**Blinkhorn 1983**	-	6.25 (5.55), 184	-	4.72 (4.19), 245	-	-	-0.32	0.10	1972
20	**Ringelberg 1979 (2Fconc/2agents)**	-	6.25 (7.36), 186	-	4.82 (5.46), 341	-	-	-0.23	0.09	1973
21	**Koch 1967**	-	21.06 (9.56),	-	16.13 (6.82),	-	-	-0.56	0.13	1962

	Trial	Mean (standard deviation), total number of patients						SMD	SE	Year
		No treatment	Placebo	Dentifrice	Rinse	Gel	Varnish			
			202		85					
86	**DePaola 1980**	-	5.42 (5.12), 142	-	4.47 (4.54), 129	-	-	-0.20	0.12	1977
87	**Heidmann 1992**	-	0.74 (1.61), 545	-	0.70 (1.56), 538	-	-	-0.03	0.06	1983
88	**Horowitz 1971(grade1)**	-	1.29 (1.88), 123	-	1.08 (1.96), 133	-	-	-0.11	0.13	1967
89	**Laswell 1975 (2Fconc/2freq)**	-	1.62 (2.47), 97	-	1.05 (1.95), 226	-	-	-0.27	0.12	1971
90	**Driscoll 1982 (2Fconc/2freq)**	-	2.24 (2.96), 151	-	1.40 (2.28), 373	-	-	-0.34	0.10	1977
91	**Packer 1975 (2Fconc/2freq)**	-	2.65 (4.15), 97	-	1.72 (3.93), 188	-	-	-0.23	0.13	1971
92	**Poulsen 1984**	-	2.82 (3.35), 174	-	2.48 (3.13), 191	-	-	-0.11	0.10	1979
93	**Horowitz 1971a(grade 5)**	-	2.92 (4.20), 110	-	1.65 (2.77), 98	-	-	-0.35	0.14	1967
94	**Radike 1973**	-	3.02 (3.09), 378	-	2.02 (2.46), 348	-	-	-0.36	0.07	1970
95	**Petersson 1998**	-	3.21 (4.74), 70	-	2.75 (4.46), 69	-	-	-0.10	0.17	1994
96	**Duany 1981(3Fconc)**	-	3.24 (4.26), 225	-	2.82 (4.39), 711	-	-	-0.10	0.08	1977
97	**Ringelberg 1982 (2Fconc/2freq)**	-	3.34 (4.42), 249	-	2.60 (5.46), 989	-	-	-0.14	0.07	1979
98	**Spets-Happonen 1991**	-	3.40 (5.60), 51	-	2.50 (3.20), 44	-	-	-0.19	0.21	1985
99	**Heifetz 1982 (2Fconc/2freq)**	-	4.08 (5.46), 204	-	2.65 (3.83), 394	-	-	-0.32	0.09	1976
100	**Koch 1967b**	-	4.93 (5.15), 137	-	4.82 (4.16), 114	-	-	-0.02	0.13	1962
101	**Molina 1987**	-	5.12 (4.06), 150	-	3.57 (3.41), 145	-	-	-0.41	0.12	1983
102	**McConchie 1977(2Fconc)**	-	5.74 (4.96), 247	-	4.72 (4.45), 496	-	-	-0.22	0.08	1970

	Trial	Mean (standard deviation), total number of patients						SMD	SE	Year
		No treatment	Placebo	Dentifrice	Rinse	Gel	Varnish			
103	**Koch 1967a**	-	6.16 (5.21), 134	-	4.60 (4.00), 117	-	-	-0.33	0.13	1962
104	**Moreira 1972 (3Freq)**	-	6.20 (5.17), 50	-	5.16 (4.68), 150	-	-	-0.22	0.16	1968
105	**Finn 1975(2Fconc)**	-	7.47 (5.73), 161	-	6.22 (5.18), 292	-	-	-0.23	0.10	1972
106	**van Wyk 1986 (2Fconc)**	-	7.50 (5.74), 192	-	5.26 (4.73), 377	-	-	-0.44	0.09	1981
107	**Heifetz 1973(2Fagents)**	-	7.53 (6.45), 154	-	5.10 (5.24), 259	-	-	-0.42	0.10	1969
108	**DePaola 1977(2Fagents)**	-	7.55 (5.77), 158	-	4.41 (4.29), 317	-	-	-0.65	0.10	1974
109	**Bastos 1989(2Fagents)**	-	9.79 (6.65), 140	-	7.02 (5.54), 280	-	-	-0.47	0.10	1977
110	**Rugg-Gunn 1973**	-	10.22 (7.24), 212	-	6.57 (4.79), 222	-	-	-0.60	0.10	1969
111	**Gallagher 1974**	-	11.72 (7.34), 288	-	10.06 (6.75), 306	-	-	-0.24	0.08	1970
86	**DePaola 1980**	-	5.42 (5.12), 142	-	-	5.09 (4.41), 128	-	-0.07	0.12	1977
24	**Mainwaring 1978**	-	8.27 (6.62), 316	-	-	7.10 (5.62), 315	-	-0.19	0.08	1974
22	**Marthaler 1970 (Age 2)**	-	3.95 (2.70), 20	-	-	3.33 (3.20), 21	-	-0.21	0.31	1966
23	**Marthaler 1970 (1)**	-	4.40 (3.14), 57	-	-	2.63 (2.52), 63	-	-0.63	0.19	1966
112	**Gisselsson 1999 (2Fconc/2Fagents)**	-	0.50 (1.03), 98	-	-	0.44 (0.84), 182	-	-0.07	0.13	1993
113	**Shern 1976(2Fagents/2intervals)**	-	1.09 (2.70), 173	-	-	0.79 (2.15), 389	-	-0.13	0.09	1973
114	**Szwjeda 1972**	-	2.18 (2.35), 153	-	-	2.10 (2.55), 163	-	-0.03	0.11	1968
115	**Olivier 1992**	-	3.24 (3.13), 207	-	-	2.94 (3.09), 224	-	-0.10	0.10	1985
116	**Cons 1970**	-	3.82 (5.11), 311	-	-	3.14 (3.83), 278	-	-0.15	0.08	1964

	Trial	Mean (standard deviation), total number of patients						SMD	SE	Year
		No treatment	Placebo	Dentifrice	Rinse	Gel	Varnish			
117	**Trubman 1973**	-	4.21 (4.12), 166	-	-	2.74 (3.13), 145	-	-0.40	0.11	1969
118	**Heifetz 1970**	-	4.26 (4.50), 148	-	-	3.94 (4.44), 161	-	-0.07	0.11	1966
119	**Hagan 1985(2Fconc)**	-	4.40 (3.86), 103	-	-	3.19 (3.86), 213	-	-0.31	0.12	1981
120	**Horowitz 1974**	-	11.48 (6.71), 117	-	-	7.68 (5.06), 116	-	-0.64	0.13	1967
121	**Tewari 1990**	-	2.16 (4.03), 307	-	-	-	0.55 (4.59), 311	-0.37	0.08	1982
122	**Borutta 1991 (2Fconc/2freq)**	-	2.90 (3.60), 90	-	-	-	2.07 (2.34), 270	-0.31	0.12	1988
123	**Clark 1985 (2Fconc)**	-	3.11 (3.54), 234	-	-	-	2.50 (3.14), 442	-0.19	0.08	1981
124	**Axelsson 1987**	-	-	1.90 (2.70), 43	2.50 (3.14), 50	-		0.20	0.21	1977
125	**Petersson 1985**	-	-	2.10 (0.82), 98		-	2.00 (2.16), 85	-0.06	0.15	1978
126	**Kirkegaard 1986**	-	-	-	1.94 (2.01), 156	-	1.61 (2.02), 163	-0.16	0.11	1978
127	**Koch 1979**	-	-	-	2.00 (2.16), 98	-	2.80 (2.70), 99	0.33	0.14	1976
128	**Brunn 1985**	-	-	-	3.50 (3.37), 126	-	3.30 (3.35), 125	-0.06	0.13	1981
129	**Seppa 1987**	-	-	-	7.64 (6.42), 123	-	10.37 (7.56), 62	0.40	0.16	1991
130	**Seppa 1995**	-	-	-	-	3.10 (3.70), 125	3.60 (4.60), 129	0.12	0.13	1991

Table S2: Fluoride dataset.

SE: standard error; SMD: standardised mean difference.

Interventions were coded as no treatment=1, placebo=2, dentifrice=3, rinse=4, gel=5 and varnish=6.

	Median (95% credibility interval)		
	**Artemether versus quinine**	**Artesunate versus quinine**	**Artesunate versus artemether**
**Log odds ratio (centred at mean 14.73)**	-0.24 (-0.46, -0.01)	-0.45 (-0.59, -0.31)	-0.21 (-0.47, 0.04)
**Regression coefficient for in the treatment by age interaction**	-0.014 (-0.031, 0.004)	-0.015 (-0.026, -0.003)	-0.001 (-0.020, 0.018)
**Log odds ratio for age 0 years.**	-0.04 (-0.37, 0.29)	-0.23 (-0.44, -0.03)	-0.20 (-0.58, 0.19)
**Log odds ratio for age 5 years.**	-0.10 (-0.38, 0.17)	-0.31 (-0.47, -0.14)	-0.20 (-0.52, 0.12)
**Log odds ratio for age 10 years.**	-0.17 (-0.41, 0.06)	-0.38 (-0.53, -0.24)	-0.21 (-0.48, 0.06)
**Log odds ratio for age 15 years.**	-0.24 (-0.46, -0.02)	-0.45 (-0.60, -0.31)	-0.21 (-0.47, 0.04)
**Log odds ratio for age 20 years.**	-0.31 (-0.55, -0.06)	-0.53 (-0.69, -0.36)	-0.22 (-0.49, 0.05)
**Log odds ratio for age 25 years.**	-0.38 (-0.67, -0.08)	-0.60 (-0.80, -0.40)	-0.22 (-0.54, 0.09)
**Log odds ratio for age 30 years.**	-0.45 (-0.80, -0.09)	-0.67 (-0.92, -0.43)	-0.23 (-0.61, 0.15)
**Log odds ratio for age 35 years.**	-0.51 (-0.94, -0.09)	-0.75 (-1.04, -0.45)	-0.23 (-0.69, 0.22)

Table S3: Results from the fixed-effect model including independent treatment by covariate interactions for the malaria dataset.

	Median (95% credibility interval)							
	**SMD (centred at mean 1972)**	**Regression coefficient for in the treatment by year interaction**	**SMD for 1954**	**SMD for 1960**	**SMD for 1970**	**SMD for 1980**	**SMD for 1990**	**SMD for 1994**
PL vs. NT	-0.20 (-0.33, -0.07)	-0.007 (-0.022, 0.009)	-0.09 (-0.41, 0.23)	-0.13 (-0.37, 0.11)	-0.19 (-0.33, -0.06)	-0.26 (-0.43, -0.09)	-0.32 (-0.62, -0.03)	-0.35 (-0.70, 0.00)
DE vs. NT	-0.52 (-0.65, -0.38)	-0.003 (-0.020, 0.014)	-0.46 (-0.80, -0.13)	-0.48 (-0.73, -0.23)	-0.51 (-0.65, -0.37)	-0.54 (-0.73, -0.35)	-0.57 (-0.91, -0.24)	-0.59 (-0.98, -0.19)
RI vs. NT	-0.50 (-0.63, -0.36)	-0.003 (-0.019, 0.014)	-0.45 (-0.80, -0.10)	-0.47 (-0.73, -0.20)	-0.49 (-0.64, -0.35)	-0.52 (-0.69, -0.35)	-0.55 (-0.85, -0.24)	-0.56 (-0.92, -0.19)
GE vs. NT	-0.46 (-0.57, -0.34)	0.002 (-0.014, 0.017)	-0.48 (-0.79, -0.18)	-0.47 (-0.69, -0.25)	-0.46 (-0.58, -0.33)	-0.44 (-0.61, -0.27)	-0.42 (-0.72, -0.13)	-0.42 (-0.77, -0.07)
VA vs. NT	-0.71 (-0.99, -0.44)	0.019 (-0.006, 0.043)	-1.04 (-1.72, -0.37)	-0.93 (-1.46, -0.41)	-0.74 (-1.05, -0.44)	-0.56 (-0.74, -0.38)	-0.37 (-0.67, -0.07)	-0.30 (-0.68, 0.09)
DE vs. PL	-0.31 (-0.37, -0.26)	0.003 (-0.004, 0.011)	-0.37 (-0.49, -0.25)	-0.35 (-0.43, -0.27)	-0.32 (-0.37, -0.27)	-0.28 (-0.38, -0.19)	-0.25 (-0.42, -0.08)	-0.24 (-0.43, -0.04)
RI vs. PL	-0.29 (-0.36, -0.22)	0.004 (-0.006, 0.014)	-0.36 (-0.56, -0.16)	-0.34 (-0.49, -0.19)	-0.30 (-0.38, -0.22)	-0.26 (-0.35, -0.17)	-0.22 (-0.40, -0.05)	-0.21 (-0.42, 0.00)
GE vs. PL	-0.25 (-0.36, -0.15)	0.008 (-0.004, 0.020)	-0.39 (-0.65, -0.14)	-0.35 (-0.54, -0.16)	-0.26 (-0.38, -0.15)	-0.18 (-0.31, -0.06)	-0.10 (-0.32, 0.12)	-0.07 (-0.33, 0.20)
VA vs. PL	-0.51 (-0.78, -0.24)	0.025 (0.002, 0.049)	-0.95 (-1.61, -0.30)	-0.80 (-1.33, -0.28)	-0.55 (-0.86, -0.25)	-0.30 (-0.45, -0.15)	-0.05 (-0.29, 0.20)	0.05 (-0.27, 0.38)
RI vs. DE	0.02 (-0.07, 0.10)	0.001 (-0.012, 0.013)	0.01 (-0.22, 0.24)	0.01 (-0.15, 0.18)	0.02 (-0.07, 0.11)	0.02 (-0.11, 0.15)	0.03 (-0.21, 0.26)	0.03 (-0.25, 0.31)
GE vs. DE	0.06 (-0.05, 0.18)	0.005 (-0.009, 0.019)	-0.02 (-0.30, 0.25)	0.01 (-0.20, 0.21)	0.05 (-0.07, 0.17)	0.10 (-0.06, 0.26)	0.15 (-0.12, 0.42)	0.17 (-0.16, 0.50)
VA vs. DE	-0.20 (-0.47, 0.07)	0.022 (-0.003, 0.046)	-0.58 (-1.25, 0.09)	-0.45 (-0.98, 0.08)	-0.23 (-0.54, 0.07)	-0.02 (-0.18, 0.15)	0.20 (-0.09, 0.49)	0.29 (-0.09, 0.66)
GE vs. RI	0.04 (-0.08, 0.16)	0.004 (-0.010, 0.019)	-0.03 (-0.35, 0.28)	-0.01 (-0.24, 0.22)	0.04 (-0.09, 0.16)	0.08 (-0.07, 0.22)	0.12 (-0.14, 0.38)	0.14 (-0.18, 0.45)
VA vs. RI	-0.22 (-0.49, 0.05)	0.021 (-0.003, 0.045)	-0.59 (-1.26, 0.07)	-0.46 (-0.99, 0.06)	-0.25 (-0.56, 0.05)	-0.04 (-0.19, 0.11)	0.18 (-0.08, 0.43)	0.26 (-0.08, 0.60)
VA vs. GE	-0.26 (-0.54, 0.02)	0.017 (-0.006, 0.040)	-0.56 (-1.21, 0.09)	-0.45 (-0.98, 0.06)	-0.28 (-0.60, 0.03)	-0.12 (-0.29, 0.06)	0.05 (-0.21, 0.32)	0.12 (-0.22, 0.46)

Table S4: Results from the random-effects model including independent treatment by covariate interactions estimated using Winbugs (Bayesian approach) for the fluoride dataset.

DE: dentifrice; GE: gel; NT: no treatment; PL: placebo; RI: rinse; SMD: standardised mean difference; VA: varnish.

	Trial	Treatments	Average age (years)	Contribution to log odds ratio (%)			Contribution to regression coefficient for treatment by age interaction (%)		
				AR vs QU	AS vs QU	AS vs AR	AR vs QU	AS vs QU	AS vs AR
1	Adam 2002	AR vs QU	3.85	0.39	0.00	0.24	0.32	0.00	0.21
2	Aguwa 2010	AR vs QU	3.50	2.97	0.02	1.87	2.53	0.03	1.68
3	Hien 1996	AR vs QU	30.00	20.87	3.71	10.95	24.13	4.48	13.42
4	Huda 2003	AR vs QU	6.20	2.26	0.03	1.40	1.42	0.04	0.97
5	Karbwang 1992	AR vs QU	31.05	0.85	0.16	0.44	1.04	0.19	0.58
6	Karbwang 1995	AR vs QU	26.50	4.29	0.67	2.30	3.92	0.81	2.13
7	Minta 2005	AR vs QU	6.80	1.34	0.03	0.82	0.77	0.03	0.53
8	Murphy 1996	AR vs QU	2.33	5.08	0.08	3.22	4.83	0.10	3.18
9	Ojuawo 1998	AR vs QU	3.93	0.67	0.00	0.42	0.54	0.00	0.36
10	Olumese 1999	AR vs QU	3.15	5.01	0.04	3.16	4.42	0.06	2.92
11	Osonuga 2009	AR vs QU	7.00	0.26	0.01	0.16	0.15	0.01	0.10
12	Taylor 1998	AR vs QU	3.08	4.96	0.05	3.13	4.40	0.06	2.91
13	van Hensbroek 1996	AR vs QU	3.92	25.70	0.07	16.14	21.00	0.12	14.00
14	Walker 1993	AR vs QU	3.00	1.82	0.02	1.15	1.63	0.02	1.08
15	Anh 1989	AS vs QU	34.47	0.30	0.85	0.30	0.36	1.17	0.48
16	Anh 1995	AS vs QU	30.95	0.98	3.06	1.14	1.20	3.68	1.46
17	Cao 1997	AS vs QU	5.50	0.02	0.87	0.48	0.02	0.56	0.36
18	Dondorp 2005	AS vs QU	27.90	9.46	32.22	12.56	11.60	33.52	12.87
19	Dondorp 2010	AS vs QU	2.85	1.14	49.86	29.32	1.55	46.26	27.46
20	Eltahir 2010	AS vs QU	4.50	0.00	0.28	0.16	0.00	0.21	0.13
21	Hien 1992	AS vs QU	28.50	0.45	1.49	0.58	0.55	1.60	0.62
22	Newton 2003	AS vs QU	25.00	0.58	2.17	0.88	0.71	1.90	0.69
23	Phu 2010	AS vs AR	32.25	7.65	3.11	6.58	10.13	4.05	9.29
24	Vinh 1997	AS vs AR	27.33	2.98	1.21	2.56	2.77	1.11	2.54

Table S5: Percentage contribution of each trial to each log odds ratio and coefficient using the new methods for the malaria dataset. AR: artemether; AS: artesunate; QU: quinine.

Study	Trial	Treatments	Average age (years)	Contribution to log odds ratio (%)			Contribution to regression coefficient for treatment by age interaction (%)		
				AR vs QU	AS vs QU	AS vs AR	AR vs QU	AS vs QU	AS vs AR
1	Adam 2002	AR vs QU	3.85	0.43	0.00	0.34	0.29	0.00	0.24
2	Aguwa 2010	AR vs QU	3.50	3.25	0.00	2.58	2.40	0.00	1.93
3	Hien 1996	AR vs QU	30.00	25.50	1.62	14.03	34.71	2.33	19.57
4	Huda 2003	AR vs QU	6.20	2.50	0.00	1.91	1.00	0.00	0.86
5	Karbwang 1992	AR vs QU	31.05	1.04	0.07	0.56	1.60	0.10	0.91
6	Karbwang 1995	AR vs QU	26.50	5.16	0.25	2.98	4.41	0.36	2.38
7	Minta 2005	AR vs QU	6.80	1.48	0.00	1.12	0.50	0.00	0.44
8	Murphy 1996	AR vs QU	2.33	5.53	0.00	4.46	5.09	0.00	4.02
9	Ojuawo 1998	AR vs QU	3.93	0.73	0.00	0.58	0.49	0.00	0.40
10	Olumese 1999	AR vs QU	3.15	5.47	0.00	4.36	4.33	0.00	3.46
11	Osonuga 2009	AR vs QU	7.00	0.29	0.00	0.22	0.09	0.00	0.08
12	Taylor 1998	AR vs QU	3.08	5.41	0.00	4.32	4.35	0.00	3.47
13	van Hensbroek 1996	AR vs QU	3.92	28.17	0.00	22.21	19.15	0.00	15.53
14	Walker 1993	AR vs QU	3.00	1.99	0.00	1.59	1.62	0.00	1.29
15	Anh 1989	AS vs QU	34.47	0.07	1.14	0.14	0.11	2.13	0.33
16	Anh 1995	AS vs QU	30.95	0.20	3.95	0.55	0.31	5.63	0.83
17	Cao 1997	AS vs QU	5.50	0.00	0.79	0.25	0.00	0.32	0.12
18	Dondorp 2005	AS vs QU	27.90	1.71	40.17	6.04	2.63	42.76	5.89
19	Dondorp 2010	AS vs QU	2.85	0.01	43.63	14.93	0.02	36.94	12.17
20	Eltahir 2010	AS vs QU	4.50	0.00	0.25	0.08	0.00	0.14	0.05
21	Hien 1992	AS vs QU	28.50	0.08	1.87	0.28	0.13	2.12	0.30
22	Newton 2003	AS vs QU	25.00	0.09	2.62	0.43	0.14	1.97	0.25
23	Phu 2010	AS vs AR	32.25	7.79	2.59	11.50	13.91	4.33	21.30
24	Vinh 1997	AS vs AR	27.33	3.08	1.02	4.55	2.71	0.85	4.15

Table S6: Study weight of each trial to each log odds ratio and coefficient using the existing methods proposed by Riley *et al* for the malaria dataset.[7]

AR: artemether; AS: artesunate; QU: quinine.

Study number	Trial	Treatments	Year	Contribution to SMD (%)															Contribution to regression coefficient for treatment by year interaction (%)														
				PL vs. NT	DE vs. NT	RI vs. NT	GE vs. NT	VA vs. NT	DE vs. PL	RI vs. PL	GE vs. PL	VA vs. PL	RI vs. DE	GE vs. DE	VA vs. DE	GE vs. RI	VA vs. RI	VA vs. GE	PL vs. NT	DE vs. NT	RI vs. NT	GE vs. NT	VA vs. NT	DE vs. PL	RI vs. PL	GE vs. PL	VA vs. PL	RI vs. DE	GE vs. DE	VA vs. DE	GE vs. RI	VA vs. RI	VA vs. GE
1	Torell 1965 (2Fagents)	NT vs PL	1962	3.11	2.16	1.70	2.17	0.82	0.59	1.94	1.88	1.18	0.95	1.03	0.87	0.17	0.40	0.25	4.11	2.40	1.96	2.84	1.54	0.95	2.67	2.26	1.80	1.08	0.78	0.90	0.32	0.20	0.52
1	Torell 1965 (2Fagents)	NT vs DE	1962	3.31	2.91	1.86	2.41	0.81	0.84	2.00	1.91	1.32	1.77	1.75	1.43	0.16	0.53	0.37	4.39	4.10	2.14	3.10	1.61	2.37	2.77	2.32	1.95	2.96	2.60	2.27	0.34	0.29	0.64
1	Torell 1965 (2Fagents)	NT vs RI	1962	2.74	2.04	5.23	1.92	1.59	0.20	4.63	1.66	0.21	3.16	1.06	0.12	4.08	2.16	0.53	3.03	1.71	6.76	2.28	2.45	0.83	6.39	1.41	0.05	4.28	0.36	0.35	5.31	3.43	0.91
2	Craig 1981	NT vs RI	1977	2.14	1.65	2.31	1.78	1.09	0.02	0.60	1.01	0.31	0.40	0.70	0.27	1.11	0.57	0.16	1.32	0.91	1.36	1.31	0.86	0.00	0.30	0.22	0.20	0.17	0.13	0.14	0.35	0.34	0.08
3	Moreira 1981	NT vs RI	1974	3.00	2.31	3.32	2.39	1.45	0.03	0.97	1.51	0.51	0.65	1.04	0.45	1.71	0.94	0.19	0.39	0.28	0.23	0.65	0.35	0.02	0.19	0.27	0.04	0.12	0.14	0.02	0.31	0.14	0.12
4	Ruiken 1987(Cluster)	NT vs RI	1981	2.62	2.02	2.71	2.32	1.46	0.01	0.54	1.09	0.26	0.36	0.76	0.23	1.13	0.50	0.23	3.71	2.55	4.09	3.31	2.28	0.01	1.22	1.09	0.70	0.74	0.61	0.49	1.57	1.31	0.08
5	Englander 1971	NT vs GE	1967	3.16	2.43	2.67	5.78	1.27	0.04	0.38	1.73	0.78	0.23	1.23	0.68	1.50	0.65	1.45	2.52	1.70	2.09	3.96	1.32	0.09	0.25	1.44	0.72	0.10	0.87	0.47	1.13	0.52	1.63
6	Ingraham 1970(2trays)	NT vs GE	1965	1.91	1.46	1.61	3.50	0.82	0.02	0.22	1.06	0.43	0.13	0.75	0.37	0.91	0.35	0.84	2.08	1.40	1.71	3.25	1.21	0.07	0.22	1.18	0.46	0.09	0.71	0.30	0.94	0.31	1.20
7	Mestrinho 1983	NT vs GE	1981	1.96	1.50	1.62	3.46	0.38	0.03	0.30	0.95	0.87	0.18	0.68	0.77	0.88	0.77	1.19	3.14	2.13	2.49	4.86	2.66	0.07	0.48	1.71	0.17	0.25	1.01	0.15	1.47	0.42	0.86
8	Englander 1967(2Fagents)	NT vs GE	1964	3.13	2.40	2.65	5.76	1.38	0.04	0.35	1.75	0.66	0.21	1.24	0.58	1.50	0.53	1.35	3.85	2.60	3.16	6.03	2.31	0.13	0.43	2.17	0.79	0.19	1.31	0.50	1.75	0.49	2.15
9	Abadia 1978 (2techniques)	NT vs GE	1977	2.29	1.76	1.91	4.10	0.60	0.03	0.33	1.16	0.88	0.20	0.82	0.77	1.05	0.77	1.29	1.93	1.32	1.51	2.98	1.83	0.03	0.33	1.04	0.30	0.18	0.61	0.22	0.92	0.46	0.31
10	Bryan 1970	NT vs GE	1966	2.42	1.86	2.05	4.45	1.01	0.03	0.28	1.33	0.57	0.17	0.95	0.50	1.15	0.47	1.09	2.29	1.55	1.89	3.60	1.28	0.08	0.24	1.30	0.57	0.10	0.79	0.37	1.03	0.40	1.39
11	Cobb 1980	NT vs GE	1977	2.10	1.61	1.74	3.75	0.55	0.03	0.30	1.06	0.80	0.18	0.75	0.71	0.96	0.70	1.18	1.77	1.20	1.38	2.72	1.67	0.03	0.30	0.95	0.28	0.16	0.56	0.21	0.84	0.42	0.29
12	Horowitz 1971	NT vs GE	1965	2.93	2.25	2.48	5.38	1.25	0.04	0.34	1.62	0.65	0.20	1.15	0.57	1.40	0.53	1.29	3.19	2.15	2.63	5.00	1.85	0.11	0.34	1.81	0.71	0.15	1.09	0.46	1.44	0.47	1.84
13	Bijella 1981	NT vs GE	1979	2.42	1.86	2.01	4.31	0.55	0.03	0.36	1.20	1.00	0.22	0.85	0.88	1.10	0.88	1.42	2.94	2.00	2.32	4.54	2.60	0.06	0.47	1.59	0.27	0.25	0.94	0.21	1.39	0.50	0.69
14	Modeer 1984	NT vs VA	1979	2.05	1.63	1.92	1.77	5.72	0.11	0.07	0.90	4.17	0.01	0.68	3.68	0.70	4.27	4.13	2.45	1.75	2.12	2.21	1.26	0.12	0.10	0.69	3.33	0.13	0.46	2.38	0.39	3.01	3.09
15	Bravo 1997 (cluster)	NT vs VA	1990	1.90	1.45	1.54	2.20	2.98	0.05	0.34	0.26	4.06	0.19	0.16	3.59	0.02	4.04	3.53	6.33	4.29	5.66	6.59	10.68	0.16	0.03	0.66	5.85	0.10	0.29	4.16	0.46	5.36	6.54
16	Holm 1984	NT vs VA	1977	1.34	1.07	1.28	1.09	4.67	0.09	0.09	0.65	3.62	0.01	0.50	3.19	0.53	3.69	3.53	1.14	0.84	0.97	0.93	2.16	0.11	0.08	0.45	3.18	0.11	0.31	2.28	0.24	2.89	3.07
17	Koch 1975	NT vs VA	1973	1.69	1.37	1.68	1.23	8.13	0.16	0.23	0.98	6.71	0.06	0.76	5.92	0.85	6.82	6.45	0.35	0.34	0.23	0.04	5.93	0.21	0.14	0.56	6.44	0.20	0.43	4.59	0.28	5.85	6.42
18	Ashley 1977	PL vs DE	1973	0.15	1.00	0.60	0.19	0.06	2.14	1.25	0.01	0.04	1.99	1.06	0.67	0.77	0.49	0.03	0.10	0.72	0.19	0.05	0.01	1.44	0.16	0.07	0.07	0.69	0.81	0.60	0.16	0.16	0.03
18	Ashley 1977	PL vs RI	1973	0.55	0.79	1.16	0.42	0.14	0.88	2.83	0.30	0.49	2.34	0.64	0.70	1.53	0.68	0.31	0.17	0.42	0.36	0.13	0.23	0.66	0.33	0.09	0.09	0.17	0.40	0.19	0.28	0.09	0.15
19	Blinkhorn 1983	PL vs DE	1972	0.14	0.90	0.63	0.18	0.02	1.91	1.29	0.01	0.07	1.89	0.94	0.63	0.79	0.47	0.06	0.06	0.52	0.30	0.03	0.04	1.05	0.39	0.05	0.01	0.34	0.59	0.41	0.30	0.22	0.02
19	Blinkhorn 1983	PL vs RI	1972	0.53	0.74	1.22	0.40	0.17	0.80	2.89	0.29	0.50	2.34	0.60	0.68	1.58	0.70	0.32	0.07	0.28	0.58	0.06	0.25	0.51	0.81	0.02	0.20	0.21	0.28	0.05	0.57	0.26	0.22

Stu dy nu mb er	Trial	Treatme nts	Year	Contribution to SMD (%)															Contribution to regression coefficient for treatment by year interaction (%)														
				PL vs. NT	DE vs. NT	RI vs. NT	GE vs. NT	VA vs. NT	DE vs. PL	RI vs. PL	GE vs. PL	VA vs. PL	RI vs. DE	GE vs. DE	VA vs. DE	GE vs. RI	VA vs. RI	VA vs. GE	PL vs. NT	DE vs. NT	RI vs. NT	GE vs. NT	VA vs. NT	DE vs. PL	RI vs. PL	GE vs. PL	VA vs. PL	RI vs. DE	GE vs. DE	VA vs. DE	GE vs. RI	VA vs. RI	VA vs. GE
20	Ringelberg 1979 (2Fconc/2agents)	PL vs DE	1973	0.18	1.06	0.66	0.21	0.06	2.24	1.40	0.02	0.06	2.14	1.11	0.72	0.85	0.53	0.04	0.11	0.75	0.21	0.05	0.01	1.50	0.18	0.08	0.07	0.72	0.85	0.62	0.18	0.16	0.03
20	Ringelberg 1979 (2Fconc/2agents)	PL vs RI	1973	0.57	0.85	1.21	0.44	0.14	1.00	2.94	0.31	0.50	2.47	0.71	0.74	1.59	0.72	0.31	0.18	0.46	0.38	0.13	0.23	0.74	0.34	0.09	0.09	0.20	0.45	0.22	0.29	0.10	0.15
21	Koch 1967	PL vs DE	1962	0.04	0.31	0.81	0.08	0.22	0.68	1.43	0.03	0.24	1.31	0.31	0.42	0.91	0.36	0.23	0.19	0.92	1.09	0.12	0.24	1.73	1.99	0.12	0.41	2.14	1.00	0.95	1.28	0.70	0.36
21	Koch 1967	PL vs RI	1962	0.35	0.31	1.63	0.26	0.35	0.11	3.31	0.19	0.56	2.24	0.19	0.53	1.91	0.82	0.42	0.75	0.82	2.24	0.44	0.37	0.66	4.57	0.52	1.01	3.10	0.65	0.96	2.78	1.56	0.75
22	Marthaler 1970 (Age 2)	PL vs DE	1966	0.49	0.55	0.44	0.39	0.15	0.41	0.02	1.04	0.17	0.24	0.93	0.28	0.75	0.17	0.29	0.37	0.38	0.33	0.33	0.09	0.27	0.01	0.95	0.21	0.15	0.69	0.25	0.63	0.19	0.36
22	Marthaler 1970 (Age 2)	PL vs GE	1966	1.02	0.85	0.86	0.71	0.28	0.14	0.12	2.07	0.38	0.16	1.52	0.38	1.44	0.34	0.55	0.79	0.60	0.65	0.60	0.18	0.12	0.08	1.88	0.46	0.11	1.14	0.37	1.21	0.38	0.66
23	Marthaler 1970 (1)	PL vs DE	1966	0.82	0.92	0.73	0.65	0.24	0.69	0.03	1.74	0.29	0.40	1.56	0.46	1.25	0.28	0.49	0.63	0.64	0.55	0.55	0.16	0.45	0.01	1.60	0.36	0.25	1.15	0.42	1.06	0.32	0.60
23	Marthaler 1970 (1)	PL vs GE	1966	1.92	1.57	1.61	1.33	0.52	0.21	0.24	3.89	0.72	0.27	2.82	0.70	2.69	0.64	1.03	1.48	1.11	1.22	1.12	0.35	0.20	0.15	3.54	0.86	0.20	2.13	0.68	2.26	0.71	1.24
24	Mainwaring 1978	PL vs DE	1974	1.20	1.97	1.10	1.15	0.98	2.54	0.01	2.74	0.18	1.38	3.16	0.60	2.01	0.18	1.34	0.12	0.82	0.19	0.27	0.54	2.00	0.13	0.17	0.46	1.17	1.17	1.08	0.03	0.36	0.38
24	Mainwaring 1978	PL vs GE	1974	2.54	2.41	2.12	2.14	1.54	1.07	0.35	5.52	0.13	0.81	4.39	0.44	3.82	0.01	2.26	0.22	0.28	0.36	0.46	0.76	0.95	0.27	0.28	0.61	0.69	0.67	0.79	0.00	0.42	0.48
25	Kleber 1996	PL vs DE	1994	0.07	0.97	0.03	0.02	0.36	2.49	0.16	0.12	0.39	1.25	1.14	0.40	0.01	0.33	0.30	0.23	1.85	0.06	0.15	0.33	4.43	0.23	0.13	0.15	2.30	2.30	1.79	0.07	0.27	0.24
26	Dolles 1980	PL vs DE	1974	0.04	0.22	0.01	0.01	0.05	0.62	0.06	0.05	0.08	0.30	0.27	0.12	0.00	0.05	0.05	0.00	0.21	0.00	0.01	0.04	0.46	0.00	0.01	0.04	0.25	0.25	0.21	0.01	0.04	0.04
27	Muhler 1955	PL vs DE	1954	0.15	0.11	0.07	0.07	0.21	0.01	0.11	0.12	0.11	0.06	0.08	0.10	0.02	0.15	0.15	0.30	1.40	0.08	0.17	0.21	3.56	0.29	0.22	0.03	1.78	1.78	1.33	0.05	0.13	0.10
28	Peterson 1979 (2abras)	PL vs DE	1971	0.14	0.55	0.03	0.03	0.10	1.61	0.17	0.15	0.18	0.76	0.68	0.32	0.00	0.12	0.10	0.04	0.32	0.02	0.00	0.08	0.64	0.03	0.05	0.12	0.37	0.37	0.32	0.01	0.09	0.09
29	Glass 1983 (2abras)	PL vs DE	1976	0.13	0.77	0.01	0.02	0.20	2.12	0.19	0.16	0.28	1.03	0.93	0.39	0.00	0.21	0.18	0.04	0.88	0.01	0.05	0.17	2.00	0.05	0.00	0.14	1.07	1.07	0.86	0.03	0.16	0.15
30	Forsman 1974 (1-city V (2Fconc))	PL vs DE	1970	0.13	0.48	0.03	0.03	0.08	1.40	0.16	0.14	0.15	0.66	0.59	0.28	0.01	0.09	0.08	0.05	0.19	0.02	0.01	0.06	0.35	0.05	0.06	0.10	0.22	0.22	0.20	0.01	0.07	0.07
31	Segal 1967	PL vs DE	1964	0.15	0.28	0.05	0.05	0.03	0.95	0.15	0.14	0.06	0.42	0.37	0.23	0.01	0.00	0.01	0.16	0.43	0.05	0.08	0.05	1.20	0.15	0.13	0.08	0.57	0.57	0.40	0.02	0.01	0.01
32	Kinkel 1972	PL vs DE	1969	0.14	0.48	0.03	0.04	0.06	1.43	0.17	0.15	0.15	0.67	0.60	0.30	0.01	0.09	0.07	0.08	0.10	0.02	0.03	0.04	0.11	0.07	0.08	0.11	0.10	0.10	0.12	0.00	0.06	0.07
33	Reed 1973 (3Fconc)	PL vs DE	1970	0.15	0.56	0.03	0.03	0.09	1.63	0.18	0.16	0.18	0.77	0.69	0.33	0.01	0.11	0.09	0.06	0.23	0.02	0.02	0.07	0.40	0.05	0.07	0.12	0.25	0.26	0.24	0.01	0.08	0.08
34	Cahen 1982 (2Fagents/2abras)	PL vs DE	1977	0.15	0.88	0.01	0.01	0.24	2.42	0.21	0.18	0.32	1.18	1.06	0.44	0.00	0.24	0.21	0.06	1.08	0.01	0.06	0.21	2.47	0.07	0.01	0.16	1.32	1.32	1.06	0.04	0.19	0.18
35	Muhler 1970	PL vs DE	1967	0.13	0.37	0.03	0.04	0.02	1.14	0.15	0.14	0.11	0.52	0.46	0.25	0.01	0.05	0.04	0.10	0.10	0.03	0.04	0.01	0.38	0.09	0.09	0.09	0.15	0.15	0.08	0.00	0.03	0.04

Study number	Trial	Treatments	Year	Contribution to SMD (%)															Contribution to regression coefficient for treatment by year interaction (%)														
				PL vs. NT	DE vs. NT	RI vs. NT	GE vs. NT	VA vs. NT	DE vs. PL	RI vs. PL	GE vs. PL	VA vs. PL	RI vs. DE	GE vs. DE	VA vs. DE	GE vs. RI	VA vs. RI	VA vs. GE	PL vs. NT	DE vs. NT	RI vs. NT	GE vs. NT	VA vs. NT	DE vs. PL	RI vs. PL	GE vs. PL	VA vs. PL	RI vs. DE	GE vs. DE	VA vs. DE	GE vs. RI	VA vs. RI	VA vs. GE
36	Thomas 1966 (2abras)	PL vs DE	1961	0.14	0.15	0.05	0.05	0.08	0.61	0.13	0.13	0.01	0.25	0.21	0.18	0.01	0.04	0.05	0.19	0.69	0.05	0.10	0.09	1.82	0.18	0.15	0.06	0.89	0.89	0.65	0.03	0.04	0.03
37	Reed 1975	PL vs DE	1968	0.12	0.38	0.03	0.03	0.04	1.16	0.14	0.13	0.11	0.54	0.48	0.24	0.01	0.06	0.05	0.08	0.00	0.03	0.03	0.02	0.13	0.07	0.07	0.09	0.03	0.03	0.01	0.00	0.04	0.05
38	Zacherl 1973	PL vs DE	1970	0.13	0.48	0.03	0.03	0.08	1.41	0.16	0.14	0.16	0.66	0.59	0.28	0.01	0.09	0.08	0.05	0.20	0.02	0.01	0.06	0.35	0.05	0.06	0.10	0.22	0.22	0.20	0.01	0.07	0.07
39	James 1967	PL vs DE	1962	0.15	0.21	0.06	0.06	0.07	0.79	0.15	0.14	0.03	0.33	0.28	0.21	0.02	0.03	0.04	0.20	0.67	0.06	0.10	0.09	1.78	0.19	0.15	0.07	0.87	0.87	0.63	0.03	0.03	0.02
40	Lind 1974	PL vs DE	1970	0.15	0.55	0.03	0.03	0.09	1.63	0.18	0.16	0.18	0.77	0.69	0.33	0.01	0.11	0.09	0.06	0.23	0.02	0.02	0.07	0.40	0.05	0.07	0.12	0.25	0.26	0.24	0.01	0.08	0.08
41	Mergele 1968	PL vs DE	1964	0.13	0.25	0.04	0.04	0.03	0.86	0.13	0.13	0.06	0.38	0.33	0.21	0.01	0.00	0.01	0.14	0.39	0.04	0.07	0.04	1.08	0.13	0.12	0.07	0.52	0.52	0.36	0.01	0.01	0.01
42	Held 1968 (site C)	PL vs DE	1961	0.04	0.04	0.01	0.01	0.02	0.16	0.03	0.03	0.00	0.07	0.06	0.05	0.00	0.01	0.01	0.05	0.18	0.01	0.03	0.02	0.48	0.05	0.04	0.02	0.23	0.23	0.17	0.01	0.01	0.01
43	Weisenstein 1972	PL vs DE	1969	0.13	0.43	0.03	0.03	0.06	1.29	0.15	0.14	0.14	0.60	0.54	0.27	0.01	0.08	0.06	0.07	0.09	0.02	0.02	0.04	0.10	0.06	0.07	0.10	0.09	0.09	0.11	0.00	0.06	0.06
44	Slack 1967	PL vs DE	1963	0.15	0.24	0.05	0.05	0.05	0.87	0.15	0.14	0.04	0.38	0.32	0.22	0.01	0.02	0.02	0.18	0.55	0.05	0.09	0.07	1.48	0.17	0.14	0.08	0.71	0.71	0.51	0.02	0.02	0.00
45	Forsman 1974 (2-city L (2Fconc))	PL vs DE	1970	0.12	0.45	0.02	0.03	0.07	1.33	0.15	0.13	0.15	0.63	0.56	0.27	0.01	0.09	0.07	0.05	0.18	0.02	0.01	0.05	0.33	0.04	0.06	0.10	0.21	0.21	0.19	0.01	0.07	0.07
46	Horowitz 1966* (=comparison A only)	PL vs DE	1961	0.15	0.16	0.06	0.06	0.09	0.67	0.14	0.14	0.01	0.27	0.23	0.19	0.02	0.05	0.05	0.21	0.76	0.06	0.11	0.10	1.99	0.20	0.16	0.07	0.97	0.97	0.71	0.03	0.05	0.03
47	Slack 1967A	PL vs DE	1962	0.15	0.20	0.05	0.06	0.07	0.78	0.15	0.14	0.03	0.33	0.28	0.21	0.02	0.03	0.04	0.20	0.67	0.06	0.10	0.09	1.77	0.19	0.15	0.07	0.86	0.86	0.62	0.03	0.03	0.02
48	Zacherl 1981(2Fagents/2abras)	PL vs DE	1977	0.14	0.83	0.01	0.01	0.23	2.26	0.20	0.17	0.30	1.10	0.99	0.41	0.00	0.23	0.20	0.06	1.00	0.01	0.06	0.20	2.31	0.07	0.01	0.15	1.23	1.23	0.98	0.04	0.18	0.17
49	Zacherl 1970* (1)	PL vs DE	1963	0.14	0.23	0.05	0.05	0.05	0.81	0.14	0.13	0.04	0.35	0.31	0.21	0.01	0.02	0.02	0.17	0.52	0.05	0.08	0.06	1.39	0.16	0.13	0.07	0.67	0.67	0.48	0.02	0.02	0.00
50	Koch 1967d	PL vs DE	1962	0.12	0.16	0.04	0.04	0.06	0.61	0.11	0.11	0.02	0.26	0.22	0.16	0.01	0.03	0.03	0.15	0.52	0.04	0.08	0.07	1.38	0.15	0.12	0.06	0.67	0.67	0.49	0.02	0.03	0.01
51	Rule 1984	PL vs DE	1977	0.14	0.82	0.01	0.01	0.23	2.26	0.20	0.17	0.30	1.10	0.99	0.41	0.00	0.23	0.20	0.06	1.00	0.01	0.06	0.20	2.31	0.07	0.01	0.15	1.23	1.23	0.98	0.04	0.18	0.17
52	Murray 1980 (2abrasives)	PL vs DE	1974	0.14	0.71	0.02	0.02	0.17	1.99	0.19	0.16	0.25	0.96	0.86	0.37	0.00	0.18	0.15	0.01	0.68	0.00	0.03	0.14	1.50	0.02	0.02	0.14	0.82	0.82	0.67	0.03	0.14	0.13
53	Gish 1966* (2examiners)	PL vs DE	1963	0.13	0.20	0.04	0.05	0.05	0.74	0.12	0.12	0.04	0.32	0.28	0.19	0.01	0.01	0.02	0.15	0.47	0.04	0.08	0.06	1.26	0.14	0.12	0.07	0.61	0.61	0.43	0.02	0.02	0.00
54	Zacherl 1972A (4Fagents)	PL vs DE	1969	0.14	0.47	0.03	0.04	0.06	1.42	0.16	0.15	0.15	0.66	0.59	0.29	0.01	0.08	0.07	0.08	0.10	0.02	0.03	0.04	0.11	0.07	0.07	0.11	0.10	0.10	0.12	0.00	0.06	0.07
55	Brudevold 1966* (2Fagents/2abras)	PL vs DE	1961	0.16	0.17	0.06	0.06	0.10	0.71	0.15	0.15	0.01	0.29	0.24	0.20	0.02	0.05	0.06	0.22	0.80	0.06	0.11	0.11	2.10	0.21	0.17	0.07	1.03	1.03	0.75	0.03	0.05	0.03

Study number	Trial	Treatments	Year	Contribution to SMD (%)															Contribution to regression coefficient for treatment by year interaction (%)														
				PL vs. NT	DE vs. NT	RI vs. NT	GE vs. NT	VA vs. NT	DE vs. PL	RI vs. PL	GE vs. PL	VA vs. PL	RI vs. DE	GE vs. DE	VA vs. DE	GE vs. RI	VA vs. RI	VA vs. GE	PL vs. NT	DE vs. NT	RI vs. NT	GE vs. NT	VA vs. NT	DE vs. PL	RI vs. PL	GE vs. PL	VA vs. PL	RI vs. DE	GE vs. DE	VA vs. DE	GE vs. RI	VA vs. RI	VA vs. GE
56	Hargreaves 1973(Age 1)	PL vs DE	1968	0.12	0.37	0.03	0.03	0.04	1.11	0.14	0.12	0.11	0.52	0.46	0.24	0.01	0.06	0.05	0.08	0.00	0.02	0.03	0.02	0.13	0.07	0.07	0.08	0.03	0.03	0.01	0.00	0.04	0.05
57	Hanachowicz 1984	PL vs DE	1979	0.13	0.92	0.00	0.01	0.27	2.48	0.21	0.17	0.34	1.21	1.10	0.44	0.00	0.26	0.23	0.10	1.24	0.02	0.08	0.24	2.88	0.10	0.03	0.16	1.52	1.53	1.21	0.05	0.21	0.19
58	Abrams 1980 (2abras)	PL vs DE	1976	0.14	0.80	0.01	0.02	0.21	2.20	0.20	0.17	0.29	1.07	0.96	0.40	0.00	0.21	0.19	0.04	0.91	0.01	0.05	0.18	2.07	0.05	0.00	0.15	1.11	1.11	0.89	0.04	0.17	0.16
59	Glass 1978	PL vs DE	1974	0.12	0.59	0.01	0.02	0.14	1.65	0.16	0.14	0.21	0.79	0.71	0.31	0.00	0.15	0.13	0.01	0.56	0.00	0.02	0.12	1.25	0.01	0.02	0.11	0.68	0.68	0.55	0.02	0.11	0.11
60	Andlaw 1975	PL vs DE	1970	0.14	0.52	0.03	0.03	0.08	1.54	0.17	0.15	0.17	0.73	0.65	0.31	0.01	0.10	0.09	0.06	0.21	0.02	0.02	0.06	0.38	0.05	0.07	0.11	0.24	0.24	0.22	0.01	0.08	0.08
61	Howat 1978	PL vs DE	1974	0.13	0.64	0.02	0.02	0.15	1.79	0.17	0.15	0.23	0.86	0.78	0.34	0.00	0.16	0.14	0.01	0.61	0.00	0.02	0.13	1.36	0.01	0.02	0.12	0.74	0.74	0.60	0.02	0.12	0.12
62	Marthaler 1965(1)	PL vs DE	1958	0.12	0.03	0.05	0.05	0.12	0.31	0.10	0.11	0.04	0.10	0.08	0.13	0.02	0.08	0.08	0.21	0.87	0.06	0.11	0.13	2.23	0.20	0.15	0.04	1.11	1.11	0.82	0.03	0.07	0.05
63	Hodge 1980 (2Fagents/ 2abrasives)	PL vs DE	1976	0.13	0.74	0.01	0.01	0.20	2.05	0.19	0.16	0.27	0.99	0.90	0.37	0.00	0.20	0.17	0.04	0.84	0.01	0.05	0.17	1.93	0.05	0.00	0.14	1.03	1.03	0.83	0.03	0.15	0.15
64	Hargreaves 1973(Age 2)	PL vs DE	1968	0.12	0.36	0.03	0.03	0.04	1.09	0.13	0.12	0.11	0.51	0.45	0.23	0.01	0.06	0.04	0.08	0.00	0.02	0.03	0.02	0.12	0.07	0.07	0.08	0.03	0.03	0.01	0.00	0.04	0.04
65	Held 1968 (site B)	PL vs DE	1961	0.04	0.04	0.01	0.02	0.02	0.17	0.04	0.04	0.00	0.07	0.06	0.05	0.00	0.01	0.01	0.05	0.20	0.02	0.03	0.03	0.52	0.05	0.04	0.02	0.25	0.25	0.19	0.01	0.01	0.01
66	Jackson 1967	PL vs DE	1962	0.16	0.21	0.06	0.06	0.07	0.80	0.15	0.15	0.03	0.34	0.29	0.21	0.02	0.03	0.04	0.20	0.68	0.06	0.10	0.09	1.80	0.19	0.16	0.07	0.88	0.88	0.63	0.03	0.04	0.02
67	Fogels 1979 (2abras)	PL vs DE	1972	0.15	0.64	0.02	0.03	0.13	1.84	0.19	0.17	0.22	0.87	0.78	0.36	0.00	0.15	0.12	0.03	0.46	0.01	0.01	0.10	0.97	0.02	0.05	0.13	0.55	0.55	0.46	0.02	0.11	0.11
68	Marthaler 1974	PL vs DE	1966	0.08	0.19	0.02	0.02	0.00	0.62	0.08	0.08	0.05	0.28	0.25	0.14	0.01	0.02	0.01	0.07	0.12	0.02	0.03	0.01	0.37	0.06	0.06	0.05	0.16	0.16	0.11	0.00	0.01	0.02
69	Zacherl 1972	PL vs DE	1969	0.13	0.44	0.03	0.03	0.06	1.32	0.15	0.14	0.14	0.62	0.55	0.27	0.01	0.08	0.06	0.07	0.10	0.02	0.02	0.04	0.10	0.06	0.07	0.10	0.09	0.10	0.11	0.00	0.06	0.06
70	Peterson 1967* (2Fagents/ 2abras)	PL vs DE	1964	0.15	0.29	0.05	0.05	0.03	0.99	0.15	0.15	0.06	0.44	0.38	0.24	0.01	0.00	0.01	0.17	0.45	0.05	0.08	0.05	1.25	0.16	0.13	0.08	0.59	0.59	0.42	0.02	0.01	0.01
71	Naylor 1967	PL vs DE	1961	0.16	0.17	0.06	0.06	0.10	0.71	0.15	0.15	0.01	0.29	0.24	0.20	0.02	0.05	0.06	0.22	0.80	0.06	0.11	0.11	2.10	0.21	0.17	0.07	1.03	1.03	0.75	0.03	0.05	0.03
72	Naylor 1979	PL vs DE	1973	0.13	0.63	0.02	0.02	0.14	1.78	0.18	0.15	0.22	0.85	0.77	0.34	0.00	0.15	0.13	0.01	0.53	0.01	0.02	0.11	1.15	0.00	0.03	0.13	0.63	0.64	0.53	0.02	0.11	0.11
73	Koch 1967c	PL vs DE	1963	0.06	0.10	0.02	0.02	0.02	0.35	0.06	0.06	0.02	0.15	0.13	0.09	0.01	0.01	0.01	0.07	0.22	0.02	0.04	0.03	0.60	0.07	0.06	0.03	0.29	0.29	0.21	0.01	0.01	0.00
74	Torell 1965a (age 1)	PL vs DE	1962	0.12	0.16	0.04	0.05	0.06	0.63	0.12	0.12	0.02	0.27	0.23	0.17	0.01	0.03	0.03	0.16	0.54	0.05	0.08	0.07	1.43	0.15	0.12	0.06	0.70	0.70	0.50	0.02	0.03	0.01
75	Mainwaring 1983 (2Fagents)	PL vs DE	1978	0.13	0.82	0.00	0.01	0.23	2.23	0.19	0.16	0.30	1.09	0.98	0.40	0.00	0.23	0.20	0.07	1.06	0.01	0.07	0.20	2.44	0.08	0.02	0.15	1.30	1.30	1.03	0.04	0.18	0.17
76	Di Maggio 1980	PL vs DE	1977	0.02	0.14	0.00	0.00	0.04	0.38	0.03	0.03	0.05	0.18	0.16	0.07	0.00	0.04	0.03	0.01	0.17	0.00	0.01	0.03	0.38	0.01	0.00	0.03	0.20	0.20	0.16	0.01	0.03	0.03
77	James 1977	PL vs DE	1970	0.14	0.53	0.03	0.03	0.08	1.55	0.17	0.15	0.17	0.73	0.65	0.31	0.01	0.10	0.09	0.06	0.22	0.02	0.02	0.06	0.38	0.05	0.07	0.11	0.24	0.24	0.23	0.01	0.08	0.08

Study number	Trial	Treatments	Year	Contribution to SMD (%)														
				PL vs. NT	DE vs. NT	RI vs. NT	GE vs. NT	VA vs. NT	DE vs. PL	RI vs. PL	GE vs. PL	VA vs. PL	RI vs. DE	GE vs. DE	VA vs. DE	GE vs. RI	VA vs. RI	VA vs. GE
78	Torell 1965b (age 2)	PL vs DE	1962	0.13	0.18	0.05	0.05	0.06	0.68	0.13	0.12	0.02	0.29	0.25	0.18	0.01	0.03	0.03
79	Fanning 1968	PL vs DE	1964	0.15	0.29	0.05	0.05	0.03	0.99	0.15	0.15	0.06	0.44	0.38	0.24	0.01	0.00	0.01
80	Slack 1971(2Fagents/2abras)	PL vs DE	1965	0.15	0.33	0.05	0.05	0.01	1.09	0.16	0.15	0.08	0.49	0.43	0.25	0.01	0.02	0.01
81	Held 1968 (site A)	PL vs DE	1962	0.05	0.06	0.02	0.02	0.02	0.24	0.04	0.04	0.01	0.10	0.09	0.07	0.00	0.01	0.01
82	Hargreaves 1973(Age 3)	PL vs DE	1968	0.12	0.36	0.03	0.03	0.04	1.11	0.14	0.12	0.11	0.51	0.46	0.23	0.01	0.06	0.05
83	Zacherl 1970* (2)	PL vs DE	1963	0.14	0.23	0.05	0.05	0.05	0.81	0.14	0.13	0.04	0.35	0.30	0.21	0.01	0.01	0.02
84	Marthaler 1965(2)	PL vs DE	1958	0.07	0.02	0.03	0.03	0.06	0.17	0.06	0.06	0.02	0.05	0.04	0.07	0.01	0.04	0.04
85	Buhe 1984 (2Fconc)	PL vs DE	1976	0.14	0.81	0.01	0.02	0.21	2.23	0.20	0.17	0.29	1.08	0.97	0.41	0.00	0.22	0.19
86	DePaola 1980	PL vs RI	1977	0.33	0.30	1.40	1.18	0.78	0.11	1.88	1.63	0.54	1.18	1.20	0.45	2.36	0.23	1.19
86	DePaola 1980	PL vs GE	1977	1.55	1.16	1.80	1.81	1.36	0.09	0.65	3.88	0.32	0.48	2.67	0.32	3.24	0.06	1.96
87	Heidmann 1992	PL vs RI	1983	0.62	0.41	0.25	0.41	0.06	0.18	1.39	0.40	0.45	0.82	0.19	0.35	0.57	0.12	0.23
88	Horowitz 1971(grade1)	PL vs RI	1967	0.37	0.21	1.05	0.25	0.21	0.18	2.35	0.23	0.43	1.45	0.07	0.33	1.29	0.54	0.29
89	Laswell 1975 (2Fconc/2freq)	PL vs RI	1971	0.40	0.24	0.85	0.27	0.17	0.17	2.06	0.25	0.42	1.26	0.09	0.32	1.09	0.43	0.27
90	Driscoll 1982 (2Fconc/2freq)	PL vs RI	1977	0.49	0.31	0.58	0.33	0.12	0.17	1.76	0.31	0.43	1.06	0.13	0.33	0.86	0.30	0.25
91	Packer 1975 (2Fconc/2freq)	PL vs RI	1971	0.39	0.23	0.83	0.27	0.17	0.17	2.02	0.25	0.41	1.24	0.09	0.31	1.07	0.43	0.26
92	Poulsen 1984	PL vs RI	1979	0.49	0.31	0.44	0.33	0.09	0.16	1.52	0.31	0.40	0.91	0.14	0.31	0.71	0.22	0.22
93	Horowitz 1971a(grade 5)	PL vs RI	1967	0.34	0.19	0.97	0.23	0.19	0.17	2.17	0.21	0.40	1.34	0.07	0.31	1.19	0.50	0.27
94	Radike 1973	PL vs RI	1970	0.49	0.29	1.12	0.33	0.23	0.22	2.67	0.31	0.53	1.64	0.11	0.40	1.43	0.58	0.34
95	Petersson 1998	PL vs RI	1994	0.42	0.29	0.32	0.27	0.05	0.08	0.11	0.27	0.21	0.03	0.15	0.16	0.13	0.17	0.07
96	Duany 1981(3Fconc)	PL vs RI	1977	0.54	0.34	0.64	0.36	0.13	0.19	1.93	0.34	0.47	1.16	0.15	0.36	0.94	0.32	0.27

Study number	Trial	Treatments	Year	Contribution to regression coefficient for treatment by year interaction (%)														
				PL vs. NT	DE vs. NT	RI vs. NT	GE vs. NT	VA vs. NT	DE vs. PL	RI vs. PL	GE vs. PL	VA vs. PL	RI vs. DE	GE vs. DE	VA vs. DE	GE vs. RI	VA vs. RI	VA vs. GE
78	Torell 1965b (age 2)	PL vs DE	1962	0.17	0.58	0.05	0.09	0.08	1.54	0.16	0.13	0.06	0.75	0.75	0.54	0.02	0.03	0.01
79	Fanning 1968	PL vs DE	1964	0.16	0.45	0.05	0.08	0.05	1.25	0.15	0.13	0.08	0.59	0.59	0.42	0.02	0.01	0.01
80	Slack 1971(2Fagents/2abras)	PL vs DE	1965	0.15	0.34	0.04	0.07	0.03	0.98	0.14	0.12	0.09	0.45	0.45	0.31	0.01	0.01	0.02
81	Held 1968 (site A)	PL vs DE	1962	0.06	0.21	0.02	0.03	0.03	0.55	0.06	0.05	0.02	0.27	0.27	0.19	0.01	0.01	0.01
82	Hargreaves 1973(Age 3)	PL vs DE	1968	0.08	0.00	0.02	0.03	0.02	0.13	0.07	0.07	0.08	0.03	0.03	0.01	0.00	0.04	0.05
83	Zacherl 1970* (2)	PL vs DE	1963	0.17	0.51	0.05	0.08	0.06	1.39	0.16	0.13	0.07	0.67	0.67	0.48	0.02	0.02	0.00
84	Marthaler 1965(2)	PL vs DE	1958	0.11	0.47	0.03	0.06	0.07	1.20	0.11	0.08	0.02	0.59	0.60	0.44	0.02	0.04	0.03
85	Buhe 1984 (2Fconc)	PL vs DE	1976	0.04	0.92	0.01	0.05	0.18	2.10	0.05	0.00	0.15	1.12	1.12	0.90	0.04	0.17	0.16
86	DePaola 1980	PL vs RI	1977	0.24	0.12	0.60	0.88	0.48	0.10	1.28	0.77	0.30	0.71	0.49	0.25	1.39	0.97	0.15
86	DePaola 1980	PL vs GE	1977	0.32	0.22	0.49	1.24	0.65	0.01	0.32	2.04	0.94	0.20	1.16	0.65	1.59	1.04	0.25
87	Heidmann 1992	PL vs RI	1983	1.11	0.65	1.62	0.70	0.15	0.26	4.09	0.69	0.75	2.31	0.26	0.43	2.34	1.53	0.37
88	Horowitz 1971(grade1)	PL vs RI	1967	0.33	0.21	1.09	0.18	0.24	0.04	2.17	0.24	0.51	1.28	0.12	0.35	1.32	0.71	0.39
89	Laswell 1975 (2Fconc/2freq)	PL vs RI	1971	0.04	0.04	0.52	0.00	0.21	0.02	0.88	0.05	0.25	0.54	0.04	0.18	0.56	0.25	0.23
90	Driscoll 1982 (2Fconc/2freq)	PL vs RI	1977	0.45	0.26	0.40	0.30	0.18	0.12	1.27	0.27	0.18	0.69	0.09	0.08	0.69	0.52	0.03
91	Packer 1975 (2Fconc/2freq)	PL vs RI	1971	0.04	0.04	0.51	0.00	0.21	0.02	0.86	0.05	0.24	0.53	0.04	0.18	0.55	0.24	0.22
92	Poulsen 1984	PL vs RI	1979	0.60	0.34	0.71	0.39	0.16	0.15	1.95	0.36	0.33	1.08	0.13	0.17	1.09	0.76	0.12
93	Horowitz 1971a(grade 5)	PL vs RI	1967	0.30	0.19	1.01	0.16	0.22	0.03	2.01	0.22	0.48	1.18	0.11	0.32	1.22	0.65	0.36
94	Radike 1973	PL vs RI	1970	0.14	0.10	0.84	0.06	0.27	0.01	1.51	0.13	0.40	0.90	0.08	0.28	0.95	0.45	0.34
95	Petersson 1998	PL vs RI	1994	1.27	0.75	2.20	0.79	0.02	0.27	5.23	0.81	1.02	2.98	0.32	0.61	3.03	1.90	0.58
96	Duany 1981(3Fconc)	PL vs RI	1977	0.50	0.28	0.44	0.33	0.20	0.13	1.39	0.29	0.20	0.76	0.10	0.09	0.75	0.57	0.03

Study number	Trial	Treatments	Year	Contribution to SMD (%)															Contribution to regression coefficient for treatment by year interaction (%)														
				PL vs. NT	DE vs. NT	RI vs. NT	GE vs. NT	VA vs. NT	DE vs. PL	RI vs. PL	GE vs. PL	VA vs. PL	RI vs. DE	GE vs. DE	VA vs. DE	GE vs. RI	VA vs. RI	VA vs. GE	PL vs. NT	DE vs. NT	RI vs. NT	GE vs. NT	VA vs. NT	DE vs. PL	RI vs. PL	GE vs. PL	VA vs. PL	RI vs. DE	GE vs. DE	VA vs. DE	GE vs. RI	VA vs. RI	VA vs. GE
97	Ringelberg 1982 (2Fconc/2freq)	PL vs RI	1979	0.57	0.36	0.52	0.38	0.11	0.19	1.76	0.36	0.46	1.06	0.16	0.36	0.83	0.26	0.26	0.69	0.40	0.82	0.45	0.19	0.17	2.26	0.42	0.38	1.26	0.15	0.20	1.27	0.88	0.14
98	Spets-Happonen 1991	PL vs RI	1985	0.31	0.20	0.05	0.20	0.02	0.08	0.56	0.20	0.21	0.32	0.10	0.16	0.20	0.02	0.10	0.62	0.37	0.96	0.39	0.06	0.14	2.38	0.39	0.45	1.35	0.15	0.26	1.37	0.88	0.23
99	Heifetz 1982 (2Fconc/2freq)	PL vs RI	1976	0.51	0.32	0.68	0.34	0.14	0.19	1.94	0.32	0.46	1.18	0.13	0.35	0.97	0.34	0.27	0.39	0.22	0.25	0.26	0.20	0.11	0.94	0.22	0.11	0.50	0.07	0.04	0.49	0.41	0.02
100	Koch 1967b	PL vs RI	1962	0.34	0.18	1.30	0.23	0.26	0.19	2.74	0.20	0.46	1.70	0.05	0.35	1.55	0.67	0.32	0.68	0.42	1.80	0.39	0.27	0.11	3.78	0.47	0.84	2.20	0.21	0.55	2.26	1.27	0.60
101	Molina 1987	PL vs RI	1983	0.48	0.32	0.19	0.32	0.05	0.14	1.08	0.31	0.35	0.64	0.15	0.27	0.44	0.09	0.18	0.86	0.50	1.27	0.54	0.12	0.20	3.19	0.54	0.59	1.80	0.20	0.34	1.82	1.19	0.29
102	McConchie 1977(2Fconc)	PL vs RI	1970	0.48	0.29	1.11	0.33	0.22	0.22	2.63	0.30	0.52	1.62	0.11	0.40	1.41	0.57	0.34	0.14	0.10	0.82	0.06	0.27	0.01	1.49	0.12	0.39	0.89	0.07	0.28	0.93	0.45	0.33
103	Koch 1967a	PL vs RI	1962	0.34	0.18	1.30	0.23	0.26	0.19	2.73	0.20	0.46	1.69	0.05	0.35	1.54	0.67	0.32	0.68	0.42	1.79	0.39	0.27	0.11	3.76	0.47	0.84	2.19	0.21	0.55	2.26	1.27	0.60
104	Moreira 1972 (3Freq)	PL vs RI	1968	0.30	0.18	0.80	0.21	0.16	0.14	1.84	0.19	0.35	1.13	0.06	0.26	1.00	0.41	0.23	0.21	0.13	0.77	0.11	0.19	0.02	1.50	0.16	0.36	0.88	0.08	0.25	0.92	0.48	0.29
105	Finn 1975(2Fconc)	PL vs RI	1972	0.46	0.27	0.89	0.31	0.18	0.19	2.22	0.29	0.46	1.36	0.11	0.35	1.16	0.46	0.29	0.04	0.01	0.42	0.05	0.23	0.04	0.61	0.01	0.20	0.39	0.02	0.16	0.41	0.14	0.21
106	van Wyk 1986 (2Fconc)	PL vs RI	1981	0.54	0.35	0.35	0.36	0.08	0.17	1.43	0.34	0.42	0.85	0.16	0.32	0.63	0.17	0.22	0.81	0.47	1.11	0.52	0.15	0.19	2.87	0.50	0.51	1.61	0.18	0.28	1.63	1.09	0.23
107	Heifetz 1973(2Fagents)	PL vs RI	1969	0.43	0.25	1.05	0.29	0.21	0.20	2.45	0.26	0.47	1.50	0.09	0.36	1.32	0.54	0.31	0.20	0.14	0.90	0.10	0.25	0.01	1.70	0.16	0.42	1.01	0.09	0.29	1.05	0.53	0.35
108	DePaola 1977(2Fagents)	PL vs RI	1974	0.47	0.29	0.76	0.32	0.16	0.18	2.02	0.29	0.44	1.23	0.12	0.34	1.04	0.39	0.27	0.20	0.11	0.09	0.15	0.21	0.07	0.14	0.10	0.05	0.04	0.02	0.06	0.03	0.12	0.11
109	Bastos 1989(2Fagents)	PL vs RI	1977	0.48	0.30	0.56	0.32	0.12	0.17	1.70	0.30	0.41	1.03	0.13	0.32	0.83	0.29	0.24	0.44	0.25	0.39	0.29	0.18	0.12	1.22	0.26	0.18	0.67	0.08	0.08	0.66	0.50	0.03
110	Rugg-Gunn 1973	PL vs RI	1969	0.44	0.25	1.07	0.30	0.22	0.20	2.50	0.27	0.48	1.54	0.09	0.37	1.35	0.55	0.32	0.21	0.14	0.92	0.10	0.25	0.01	1.74	0.17	0.43	1.03	0.09	0.30	1.07	0.54	0.35
111	Gallagher 1974	PL vs RI	1970	0.48	0.28	1.09	0.32	0.22	0.21	2.59	0.30	0.51	1.59	0.10	0.39	1.38	0.56	0.33	0.14	0.10	0.81	0.06	0.26	0.01	1.46	0.12	0.38	0.88	0.07	0.27	0.92	0.44	0.33
112	Gisselsson 1999 (2Fconc/2Fagents)	PL vs GE	1993	0.31	0.21	0.16	0.92	1.56	0.06	0.21	1.34	1.30	0.11	0.91	1.18	0.85	1.43	1.75	3.43	2.27	2.39	3.64	0.45	0.21	1.04	9.52	3.29	0.51	5.33	2.23	5.66	2.46	2.32
113	Shern 1976(2Fagents/2intervals)	PL vs GE	1973	1.92	1.41	1.56	1.51	0.96	0.20	0.34	4.07	0.30	0.12	2.75	0.21	2.76	0.17	1.48	0.37	0.27	0.42	0.10	0.46	0.03	0.14	0.40	0.18	0.07	0.25	0.11	0.36	0.09	0.43
114	Szwjeda 1972	PL vs GE	1968	2.09	1.53	1.72	1.46	0.66	0.21	0.32	4.24	0.69	0.10	2.86	0.55	2.90	0.57	1.21	1.33	0.91	1.11	0.86	0.40	0.02	0.12	3.00	0.68	0.06	1.70	0.47	1.92	0.56	1.11
115	Olivier 1992	PL vs GE	1985	0.96	0.70	0.72	1.22	1.46	0.12	0.28	2.52	0.79	0.12	1.70	0.73	1.67	0.93	1.79	2.23	1.47	1.49	2.56	0.49	0.16	0.78	6.44	2.35	0.38	3.60	1.59	3.78	1.74	1.44

Study number	Trial	Treatments	Year	Contribution to SMD (%)															Contribution to regression coefficient for treatment by year interaction (%)														
				PL vs. NT	DE vs. NT	RI vs. NT	GE vs. NT	VA vs. NT	DE vs. PL	RI vs. PL	GE vs. PL	VA vs. PL	RI vs. DE	GE vs. DE	VA vs. DE	GE vs. RI	VA vs. RI	VA vs. GE	PL vs. NT	DE vs. NT	RI vs. NT	GE vs. NT	VA vs. NT	DE vs. PL	RI vs. PL	GE vs. PL	VA vs. PL	RI vs. DE	GE vs. DE	VA vs. DE	GE vs. RI	VA vs. RI	VA vs. GE
116	Cons 1970	PL vs GE	1964	2.73	2.00	2.27	1.76	0.58	0.26	0.39	5.39	1.17	0.11	3.64	0.97	3.70	1.05	1.27	2.45	1.66	1.95	1.86	0.45	0.07	0.37	5.87	1.55	0.18	3.32	1.06	3.68	1.23	1.93
117	Trubman 1973	PL vs GE	1969	2.00	1.46	1.64	1.42	0.70	0.20	0.32	4.08	0.60	0.10	2.76	0.47	2.78	0.48	1.22	1.12	0.77	0.95	0.66	0.40	0.01	0.07	2.44	0.51	0.04	1.39	0.35	1.59	0.43	0.95
118	Heifetz 1970	PL vs GE	1966	2.22	1.63	1.83	1.48	0.58	0.22	0.33	4.43	0.85	0.10	3.00	0.69	3.03	0.74	1.15	1.72	1.17	1.39	1.23	0.39	0.04	0.22	4.02	1.01	0.11	2.28	0.69	2.54	0.81	1.38
119	Hagan 1985(2Fconc)	PL vs GE	1981	1.13	0.82	0.88	1.16	1.14	0.13	0.26	2.66	0.38	0.10	1.80	0.37	1.78	0.50	1.49	1.21	0.79	0.76	1.54	0.43	0.10	0.50	3.69	1.44	0.24	2.06	0.97	2.13	1.06	0.73
120	Horowitz 1974	PL vs GE	1967	1.93	1.41	1.59	1.32	0.56	0.19	0.29	3.88	0.69	0.09	2.62	0.56	2.66	0.59	1.06	1.37	0.93	1.12	0.94	0.36	0.03	0.15	3.14	0.76	0.08	1.78	0.52	2.00	0.61	1.11
121	Tewari 1990	PL vs VA	1982	1.31	0.90	0.79	0.95	2.71	0.28	0.69	0.76	3.43	0.30	0.40	2.96	0.13	3.24	2.74	1.94	1.20	0.88	1.06	1.05	0.31	1.33	1.42	0.51	0.62	0.64	0.24	0.04	0.25	0.32
122	Borutta 1991 (2Fconc/2freq)	PL vs VA	1988	0.65	0.47	0.41	0.32	3.03	0.09	0.32	0.53	2.49	0.16	0.32	2.24	0.19	2.70	2.46	2.97	1.95	1.50	1.11	4.48	0.21	1.79	2.83	7.09	0.95	1.50	4.89	0.67	5.55	5.73
123	Clark 1985 (2Fconc)	PL vs VA	1981	1.40	0.96	0.84	1.04	3.78	0.30	0.74	0.78	4.50	0.32	0.40	3.91	0.11	4.33	3.70	1.66	1.00	0.72	1.01	2.13	0.32	1.19	1.09	0.84	0.53	0.45	0.71	0.09	1.42	1.54
124	Axelsson 1987	DE vs RI	1977	0.20	0.57	0.32	0.18	0.04	1.00	0.87	0.09	0.09	1.12	0.56	0.38	0.47	0.27	0.04	0.22	0.63	0.23	0.14	0.01	1.05	0.66	0.14	0.18	0.98	0.65	0.52	0.36	0.20	0.10
125	Petersson 1985	DE vs VA	1978	1.09	1.39	0.71	0.93	4.82	1.33	0.50	0.49	5.31	1.05	0.99	5.11	0.05	5.26	4.55	0.54	1.05	0.17	0.57	3.70	1.50	0.49	0.04	3.39	1.12	0.83	1.81	0.31	3.39	3.59
126	Kirkegaard 1986	RI vs VA	1978	0.97	0.68	1.34	0.83	5.93	0.17	0.77	0.43	6.30	0.60	0.22	5.54	0.80	6.81	5.46	0.22	0.07	0.75	0.43	4.49	0.18	0.87	0.23	4.48	0.61	0.23	3.21	0.44	3.65	4.56
127	Koch 1979	RI vs VA	1976	0.96	0.67	1.31	0.84	6.58	0.18	0.73	0.41	6.91	0.58	0.19	6.09	0.75	7.42	6.03	0.09	0.15	0.14	0.37	5.39	0.19	0.35	0.60	5.66	0.31	0.45	4.04	0.17	5.02	5.58
128	Brunn 1985	RI vs VA	1981	0.68	0.48	0.97	0.54	2.97	0.11	0.60	0.34	3.28	0.45	0.19	2.88	0.62	3.63	2.79	0.66	0.41	1.51	0.38	1.59	0.10	1.44	0.46	1.10	0.92	0.21	0.81	1.29	0.23	1.43
129	Seppa 1987	RI vs VA	1991	0.07	0.02	0.04	0.20	4.66	0.07	0.17	0.12	4.50	0.07	0.12	3.98	0.19	4.57	4.09	1.84	1.31	3.56	0.28	5.97	0.10	3.01	2.28	7.70	1.75	1.36	5.44	3.58	8.72	6.71
130	Seppa 1995	GE vs VA	1991	0.01	0.01	0.01	1.04	4.07	0.04	0.04	1.07	3.91	0.00	0.73	3.46	0.76	4.01	3.04	0.54	0.37	0.25	4.48	6.58	0.00	1.15	4.93	7.27	0.69	2.82	5.09	4.09	6.06	10.62

Table S7: Percentage contribution of each trial to each SMD and coefficient using the new methods for the fluoride dataset.

DE: dentifrice; GE: gel; NT: no treatment; PL: placebo; RI: rinse; SMD: standardised mean difference; VA: varnish.

	Parameter estimate (95% confidence interval)	
	SMD (centred at mean 1972)	Regression coefficient for in the treatment by year interaction
PL vs. NT	-0.20 (-0.32, -0.08)	-0.006 (-0.020, 0.008)
DE vs. NT	-0.50 (-0.62, -0.37)	-0.002 (-0.018, 0.013)
RI vs. NT	-0.49 (-0.62, -0.37)	-0.002 (-0.017, 0.013)
GE vs. NT	-0.45 (-0.55, -0.34)	0.002 (-0.012, 0.016)
VA vs. NT	-0.69 (-0.95, -0.43)	0.018 (-0.005, 0.040)
DE vs. PL	-0.30 (-0.35, -0.25)	0.004 (-0.003, 0.010)
RI vs. PL	-0.29 (-0.36, -0.23)	0.004 (-0.005, 0.013)
GE vs. PL	-0.25 (-0.34, -0.15)	0.008 (-0.003, 0.018)
VA vs. PL	-0.49 (-0.74, -0.24)	0.023 (0.002, 0.045)
RI vs. DE	0.01 (-0.07, 0.08)	0.000 (-0.011, 0.011)
GE vs. DE	0.05 (-0.05, 0.16)	0.004 (-0.009, 0.017)
VA vs. DE	-0.19 (-0.45, 0.06)	0.020 (-0.003, 0.042)
GE vs. RI	0.05 (-0.06, 0.16)	0.004 (-0.010, 0.017)
VA vs. RI	-0.20 (-0.45, 0.05)	0.019 (-0.003, 0.041)
VA vs. GE	-0.24 (-0.51, 0.02)	0.016 (-0.005, 0.037)

Table S8: Results from the random-effects model including independent treatment by covariate interactions estimated using Stata (frequentist approach) for the fluoride dataset.

DE: dentifrice; GE: gel; NT: no treatment; PL: placebo; RI: rinse; SMD: standardised mean difference; VA: varnish.

The between-trial variance is 0.02.

Study number	Trial	Treatments	Contribution to SMD (%)															Contribution to regression coefficient for treatment by year interaction (%)														
			PL vs. NT	DE vs. NT	RI vs. NT	GE vs. NT	VA vs. NT	DE vs. PL	RI vs. PL	GE vs. PL	VA vs. PL	RI vs. DE	GE vs. DE	VA vs. DE	GE vs. RI	VA vs. RI	VA vs. GE	PL vs. NT	DE vs. NT	RI vs. NT	GE vs. NT	VA vs. NT	DE vs. PL	RI vs. PL	GE vs. PL	VA vs. PL	RI vs. DE	GE vs. DE	VA vs. DE	GE vs. RI	VA vs. RI	VA vs. GE
1	Torell 1965 (2Fagents)	NT vs PL vs DE vs RI	22.81	20.64	24.08	8.16	1.42	0.37	8.53	7.31	1.18	6.57	6.28	1.21	9.29	1.52	0.06	26.91	24.83	28.31	11.27	4.00	2.16	11.53	6.23	2.02	10.57	6.15	2.48	9.67	2.96	0.14
2	Craig 1981	NT vs RI	3.54	3.15	4.39	1.78	0.48	0.00	0.37	0.68	0.03	0.27	0.56	0.03	1.16	0.11	0.02	1.00	0.85	1.17	0.82	0.25	0.00	0.06	0.02	0.02	0.04	0.02	0.02	0.09	0.06	0.00
3	Moreira 1981	NT vs RI	5.43	4.83	7.08	2.51	0.65	0.00	0.77	1.21	0.07	0.57	1.00	0.07	2.17	0.25	0.02	0.09	0.08	0.04	0.18	0.04	0.00	0.02	0.02	0.00	0.01	0.02	0.00	0.05	0.00	0.01
4	Ruiken 1987(Cluster)	NT vs RI	4.18	3.73	4.79	2.38	0.67	0.00	0.24	0.63	0.02	0.18	0.52	0.01	0.95	0.06	0.03	5.74	4.82	7.60	3.82	1.31	0.00	0.80	0.40	0.15	0.55	0.29	0.13	1.26	0.54	0.00
5	Englander 1971	NT vs GE	4.23	3.75	3.24	10.28	0.35	0.00	0.08	1.18	0.13	0.05	1.02	0.13	1.24	0.09	0.55	1.91	1.53	1.42	4.01	0.33	0.01	0.03	0.58	0.09	0.01	0.48	0.07	0.53	0.05	0.49
6	Ingraham 1970(2trays)	NT vs GE	2.34	2.07	1.80	5.76	0.22	0.00	0.04	0.68	0.06	0.03	0.59	0.06	0.71	0.04	0.29	2.03	1.63	1.49	4.22	0.44	0.01	0.03	0.59	0.06	0.01	0.49	0.05	0.57	0.03	0.41
7	Mestrinho 1983	NT vs GE	2.43	2.16	1.80	5.32	0.06	0.00	0.07	0.46	0.23	0.04	0.40	0.23	0.55	0.17	0.50	4.23	3.47	2.91	8.36	1.88	0.00	0.13	1.03	0.01	0.06	0.81	0.01	1.15	0.05	0.19
8	Englander 1967(2Fagents)	NT vs GE	4.17	3.70	3.21	10.34	0.41	0.00	0.08	1.24	0.10	0.05	1.07	0.09	1.28	0.06	0.49	4.68	3.77	3.42	9.70	1.07	0.01	0.08	1.35	0.12	0.03	1.11	0.09	1.30	0.05	0.89
9	Abadia 1978 (2techniques)	NT vs GE	2.91	2.58	2.18	6.57	0.11	0.00	0.07	0.62	0.21	0.05	0.54	0.20	0.72	0.15	0.53	1.50	1.24	1.01	2.91	0.81	0.00	0.05	0.34	0.02	0.03	0.27	0.02	0.40	0.05	0.03
10	Bryan 1970	NT vs GE	3.08	2.73	2.36	7.54	0.27	0.00	0.06	0.88	0.09	0.04	0.76	0.08	0.92	0.05	0.39	1.98	1.59	1.46	4.14	0.39	0.01	0.03	0.59	0.07	0.01	0.48	0.06	0.55	0.04	0.45
11	Cobb 1980	NT vs GE	2.62	2.32	1.96	5.91	0.10	0.00	0.07	0.56	0.19	0.04	0.49	0.18	0.65	0.14	0.48	1.35	1.12	0.91	2.62	0.73	0.00	0.05	0.31	0.02	0.03	0.24	0.02	0.36	0.05	0.02
12	Horowitz 1971	NT vs GE	3.85	3.42	2.96	9.49	0.36	0.00	0.07	1.12	0.10	0.04	0.97	0.09	1.16	0.06	0.47	3.34	2.69	2.46	6.95	0.72	0.01	0.05	0.97	0.10	0.02	0.80	0.07	0.93	0.05	0.68
13	Bijella 1981	NT vs GE	3.11	2.76	2.32	6.91	0.10	0.00	0.08	0.63	0.26	0.05	0.55	0.25	0.74	0.19	0.60	3.20	2.64	2.19	6.29	1.53	0.00	0.10	0.76	0.01	0.05	0.60	0.02	0.87	0.06	0.11
14	Modeer 1984	NT vs VA	2.59	2.45	2.43	1.40	9.40	0.01	0.00	0.44	5.62	0.00	0.43	5.46	0.37	5.55	6.33	2.57	2.31	2.10	1.74	0.45	0.01	0.01	0.17	3.11	0.02	0.17	2.92	0.08	2.85	2.50
15	Bravo 1997 (cluster)	NT vs VA	2.35	2.05	1.70	2.24	2.23	0.00	0.08	0.04	5.03	0.04	0.03	4.93	0.00	4.71	4.32	15.96	13.04	13.89	14.45	28.69	0.02	0.00	0.13	8.94	0.01	0.06	8.31	0.09	8.46	10.35
16	Holm 1984	NT vs	1.60	1.54	1.57	0.77	9.13	0.0	0.0	0.33	6.19	0.0	0.3	6.02	0.3	6.07	6.75	0.84	0.80	0.66	0.47	1.84	0.01	0.00	0.11	4.14	0.02	0.12	3.87	0.05	3.81	3.60

Study number	Trial	Treatments	Contribution to SMD (%)															Contribution to regression coefficient for treatment by year interaction (%)														
			PL vs. NT	DE vs. NT	RI vs. NT	GE vs. NT	VA vs. NT	DE vs. PL	RI vs. PL	GE vs. PL	VA vs. PL	RI vs. DE	GE vs. DE	VA vs. DE	GE vs. RI	VA vs. RI	VA vs. GE	PL vs. NT	DE vs. NT	RI vs. NT	GE vs. NT	VA vs. NT	DE vs. PL	RI vs. PL	GE vs. PL	VA vs. PL	RI vs. DE	GE vs. DE	VA vs. DE	GE vs. RI	VA vs. RI	VA vs. GE
		VA						1	1			0	3		1																	
17	Koch 1975	NT vs VA	2.10	2.06	2.21	0.80	23.10	0.03	0.05	0.62	17.83	0.01	0.64	17.37	0.66	17.37	18.84	0.09	0.13	0.05	0.00	11.47	0.04	0.01	0.15	14.11	0.04	0.19	13.18	0.06	13.07	13.07
18	Ashley 1977	PL vs DE vs RI	0.11	0.54	0.47	0.04	0.01	2.58	3.63	0.03	0.05	4.69	0.56	0.14	0.96	0.08	0.02	0.01	0.21	0.03	0.00	0.01	0.90	0.03	0.00	0.00	0.29	0.29	0.10	0.02	0.00	0.01
19	Blinkhorn 1983	PL vs DE vs RI	0.10	0.46	0.52	0.04	0.01	2.14	3.85	0.03	0.05	4.63	0.47	0.13	1.04	0.09	0.02	0.00	0.11	0.09	0.00	0.01	0.50	0.21	0.00	0.01	0.18	0.16	0.06	0.10	0.01	0.01
20	Ringelberg 1979 (2Fconc/2agents)	PL vs DE vs RI	0.11	0.58	0.49	0.04	0.01	2.71	3.79	0.03	0.05	5.07	0.60	0.15	1.01	0.09	0.02	0.01	0.22	0.03	0.00	0.01	0.95	0.03	0.00	0.00	0.30	0.30	0.10	0.02	0.00	0.01
21	Koch 1967	PL vs DE vs RI	0.05	0.07	1.12	0.02	0.02	0.31	5.98	0.02	0.07	4.50	0.05	0.08	1.80	0.14	0.04	0.18	0.53	1.63	0.05	0.03	1.57	8.08	0.07	0.19	7.24	0.55	0.42	2.91	0.53	0.10
22	Marthaler 1970 (Age 2)	PL vs DE vs GE	0.92	0.97	0.70	0.33	0.03	0.26	0.02	3.42	0.07	0.11	3.18	0.09	2.32	0.05	0.17	0.39	0.41	0.29	0.21	0.01	0.09	0.01	1.97	0.09	0.04	1.65	0.11	1.21	0.07	0.18
23	Marthaler 1970 (1)	PL vs DE vs GE	1.83	1.87	1.39	0.65	0.07	0.45	0.05	6.79	0.14	0.19	6.20	0.17	4.60	0.10	0.34	0.78	0.80	0.58	0.40	0.03	0.15	0.01	3.91	0.18	0.07	3.21	0.21	2.39	0.13	0.35
24	Mainwaring 1978	PL vs DE vs GE	2.04	3.04	1.52	1.04	0.39	3.33	0.07	8.63	0.01	1.29	9.82	0.08	5.85	0.01	1.06	0.01	0.26	0.03	0.04	0.09	1.59	0.02	0.01	0.06	0.70	0.55	0.33	0.00	0.04	0.04
25	Kleber 1996	PL vs DE	0.00	0.84	0.00	0.00	0.04	6.36	0.02	0.01	0.05	2.11	1.18	0.07	0.00	0.03	0.03	0.02	2.53	0.00	0.01	0.03	15.40	0.03	0.01	0.01	5.30	4.19	1.58	0.00	0.02	0.01
26	Dolles 1980	PL vs DE	0.00	0.08	0.00	0.00	0.00	0.71	0.01	0.00	0.00	0.22	0.12	0.01	0.00	0.00	0.00	0.00	0.06	0.00	0.00	0.00	0.32	0.00	0.00	0.00	0.12	0.09	0.04	0.00	0.00	0.00
27	Muhler 1955	PL vs DE	0.01	0.01	0.00	0.00	0.01	0.00	0.01	0.01	0.00	0.01	0.01	0.00	0.00	0.01	0.01	0.03	1.11	0.00	0.01	0.01	7.59	0.04	0.01	0.00	2.43	1.93	0.67	0.00	0.00	0.00
28	Peterson 1979 (2abras)	PL vs DE	0.01	0.19	0.00	0.00	0.00	1.82	0.02	0.01	0.01	0.54	0.29	0.03	0.00	0.00	0.00	0.00	0.05	0.00	0.00	0.00	0.22	0.00	0.00	0.00	0.10	0.08	0.04	0.00	0.00	0.00
29	Glass 1983 (2abras)	PL vs DE	0.01	0.37	0.00	0.00	0.01	3.18	0.02	0.01	0.02	0.99	0.54	0.04	0.00	0.01	0.01	0.00	0.40	0.00	0.00	0.01	2.19	0.00	0.00	0.00	0.80	0.63	0.26	0.00	0.01	0.00
30	Forsman 1974 (1-city V (2Fconc))	PL vs DE	0.01	0.15	0.00	0.00	0.00	1.46	0.01	0.01	0.01	0.43	0.23	0.02	0.00	0.00	0.00	0.00	0.02	0.00	0.00	0.00	0.07	0.00	0.00	0.00	0.04	0.03	0.02	0.00	0.00	0.00
31	Segal 1967	PL vs DE	0.01	0.04	0.00	0.00	0.00	0.61	0.01	0.01	0.00	0.16	0.08	0.02	0.00	0.00	0.00	0.01	0.10	0.00	0.00	0.00	0.83	0.01	0.00	0.00	0.24	0.19	0.06	0.00	0.00	0.00

Study number	Trial	Treatments	Contribution to SMD (%)															Contribution to regression coefficient for treatment by year interaction (%)														
			PL vs. NT	DE vs. NT	RI vs. NT	GE vs. NT	VA vs. NT	DE vs. PL	RI vs. PL	GE vs. PL	VA vs. PL	RI vs. DE	GE vs. DE	VA vs. DE	GE vs. RI	VA vs. RI	VA vs. GE	PL vs. NT	DE vs. NT	RI vs. NT	GE vs. NT	VA vs. NT	DE vs. PL	RI vs. PL	GE vs. PL	VA vs. PL	RI vs. DE	GE vs. DE	VA vs. DE	GE vs. RI	VA vs. RI	VA vs. GE
32	Kinkel 1972	PL vs DE	0.01	0.14	0.00	0.00	0.00	1.43	0.02	0.01	0.01	0.41	0.22	0.02	0.00	0.00	0.00	0.00	0.01	0.00	0.00	0.00	0.01	0.00	0.00	0.00	0.01	0.01	0.00	0.00	0.00	0.00
33	Reed 1973 (3Fconc)	PL vs DE	0.01	0.18	0.00	0.00	0.00	1.77	0.02	0.01	0.01	0.52	0.28	0.03	0.00	0.00	0.00	0.00	0.03	0.00	0.00	0.00	0.08	0.00	0.00	0.00	0.04	0.03	0.02	0.00	0.00	0.00
34	Cahen 1982 (2Fagents/2abras)	PL vs DE	0.01	0.45	0.00	0.00	0.01	3.88	0.02	0.01	0.02	1.21	0.67	0.05	0.00	0.01	0.01	0.00	0.56	0.00	0.00	0.01	3.15	0.00	0.00	0.00	1.14	0.90	0.36	0.00	0.01	0.01
35	Muhler 1970	PL vs DE	0.01	0.09	0.00	0.00	0.00	0.96	0.01	0.01	0.00	0.27	0.14	0.02	0.00	0.00	0.00	0.00	0.01	0.00	0.00	0.00	0.09	0.00	0.00	0.00	0.02	0.02	0.00	0.00	0.00	0.00
36	Thomas 1966 (2abras)	PL vs DE	0.01	0.01	0.00	0.00	0.00	0.26	0.01	0.01	0.00	0.06	0.03	0.01	0.00	0.00	0.00	0.01	0.28	0.00	0.00	0.00	2.02	0.01	0.01	0.00	0.62	0.49	0.16	0.00	0.00	0.00
37	Reed 1975	PL vs DE	0.01	0.10	0.00	0.00	0.00	1.04	0.01	0.01	0.00	0.29	0.16	0.02	0.00	0.00	0.00	0.00	0.00	0.00	0.00	0.00	0.01	0.00	0.00	0.00	0.00	0.00	0.00	0.00	0.00	0.00
38	Zacherl 1973	PL vs DE	0.01	0.15	0.00	0.00	0.00	1.47	0.01	0.01	0.01	0.43	0.23	0.02	0.00	0.00	0.00	0.00	0.02	0.00	0.00	0.00	0.07	0.00	0.00	0.00	0.04	0.03	0.02	0.00	0.00	0.00
39	James 1967	PL vs DE	0.01	0.02	0.00	0.00	0.00	0.40	0.01	0.01	0.00	0.09	0.05	0.01	0.00	0.00	0.00	0.01	0.24	0.00	0.00	0.00	1.77	0.01	0.01	0.00	0.53	0.42	0.14	0.00	0.00	0.00
40	Lind 1974	PL vs DE	0.01	0.18	0.00	0.00	0.00	1.77	0.02	0.01	0.01	0.52	0.28	0.03	0.00	0.00	0.00	0.00	0.03	0.00	0.00	0.00	0.08	0.00	0.00	0.00	0.04	0.03	0.02	0.00	0.00	0.00
41	Mergele 1968	PL vs DE	0.01	0.04	0.00	0.00	0.00	0.54	0.01	0.01	0.00	0.14	0.07	0.01	0.00	0.00	0.00	0.01	0.09	0.00	0.00	0.00	0.73	0.01	0.00	0.00	0.21	0.17	0.05	0.00	0.00	0.00
42	Held 1968 (site C)	PL vs DE	0.00	0.00	0.00	0.00	0.00	0.06	0.00	0.00	0.00	0.01	0.01	0.00	0.00	0.00	0.00	0.00	0.06	0.00	0.00	0.00	0.46	0.00	0.00	0.00	0.14	0.11	0.04	0.00	0.00	0.00
43	Weisenstein 1972	PL vs DE	0.01	0.12	0.00	0.00	0.00	1.26	0.01	0.01	0.00	0.36	0.19	0.02	0.00	0.00	0.00	0.00	0.00	0.00	0.00	0.00	0.01	0.00	0.00	0.00	0.01	0.00	0.00	0.00	0.00	0.00
44	Slack 1967	PL vs DE	0.01	0.03	0.00	0.00	0.00	0.50	0.01	0.01	0.00	0.12	0.06	0.01	0.00	0.00	0.00	0.01	0.16	0.00	0.00	0.00	1.24	0.01	0.00	0.00	0.37	0.29	0.09	0.00	0.00	0.00
45	Forsman 1974 (2-city L (2Fconc))	PL vs DE	0.01	0.14	0.00	0.00	0.00	1.38	0.01	0.01	0.01	0.40	0.22	0.02	0.00	0.00	0.00	0.00	0.02	0.00	0.00	0.00	0.06	0.00	0.00	0.00	0.03	0.03	0.01	0.00	0.00	0.00
46	Horowitz 1966* (=comparison A only)	PL vs DE	0.01	0.01	0.00	0.00	0.00	0.29	0.01	0.01	0.00	0.06	0.03	0.01	0.00	0.00	0.00	0.01	0.31	0.00	0.00	0.00	2.25	0.02	0.01	0.00	0.69	0.55	0.18	0.00	0.00	0.00
47	Slack 1967A	PL vs DE	0.01	0.02	0.00	0.00	0.00	0.40	0.01	0.01	0.00	0.09	0.05	0.01	0.00	0.00	0.00	0.01	0.23	0.00	0.00	0.00	1.75	0.01	0.01	0.00	0.53	0.42	0.14	0.00	0.00	0.00
48	Zacherl 1981(2Fagents/2abras)	PL vs DE	0.01	0.41	0.00	0.00	0.01	3.56	0.02	0.01	0.02	1.11	0.61	0.05	0.00	0.01	0.01	0.00	0.52	0.00	0.00	0.01	2.89	0.00	0.00	0.00	1.05	0.83	0.33	0.00	0.01	0.00

Study number	Trial	Treatments	Contribution to SMD (%)															Contribution to regression coefficient for treatment by year interaction (%)														
			PL vs. NT	DE vs. NT	RI vs. NT	GE vs. NT	VA vs. NT	DE vs. PL	RI vs. PL	GE vs. PL	VA vs. PL	RI vs. DE	GE vs. DE	VA vs. DE	GE vs. RI	VA vs. RI	VA vs. GE	PL vs. NT	DE vs. NT	RI vs. NT	GE vs. NT	VA vs. NT	DE vs. PL	RI vs. PL	GE vs. PL	VA vs. PL	RI vs. DE	GE vs. DE	VA vs. DE	GE vs. RI	VA vs. RI	VA vs. GE
49	Zacherl 1970* (1)	PL vs DE	0.01	0.03	0.00	0.00	0.00	0.46	0.01	0.01	0.00	0.11	0.06	0.01	0.00	0.00	0.00	0.01	0.15	0.00	0.00	0.00	1.15	0.01	0.00	0.00	0.34	0.27	0.09	0.00	0.00	0.00
50	Koch 1967d	PL vs DE	0.01	0.02	0.00	0.00	0.00	0.29	0.01	0.01	0.00	0.07	0.03	0.01	0.00	0.00	0.00	0.01	0.17	0.00	0.00	0.00	1.30	0.01	0.00	0.00	0.39	0.31	0.10	0.00	0.00	0.00
51	Rule 1984	PL vs DE	0.01	0.41	0.00	0.00	0.01	3.56	0.02	0.01	0.02	1.11	0.61	0.05	0.00	0.01	0.01	0.00	0.51	0.00	0.00	0.01	2.89	0.00	0.00	0.00	1.05	0.83	0.33	0.00	0.01	0.00
52	Murray 1980 (2abrasives)	PL vs DE	0.01	0.30	0.00	0.00	0.01	2.72	0.02	0.01	0.01	0.83	0.45	0.04	0.00	0.01	0.01	0.00	0.23	0.00	0.00	0.00	1.21	0.00	0.00	0.00	0.46	0.36	0.15	0.00	0.00	0.00
53	Gish 1966* (2examiners)	PL vs DE	0.01	0.03	0.00	0.00	0.00	0.41	0.01	0.01	0.00	0.10	0.05	0.01	0.00	0.00	0.00	0.01	0.13	0.00	0.00	0.00	1.02	0.01	0.00	0.00	0.30	0.24	0.08	0.00	0.00	0.00
54	Zacherl 1972A (4Fagents)	PL vs DE	0.01	0.14	0.00	0.00	0.00	1.41	0.02	0.01	0.01	0.41	0.22	0.02	0.00	0.00	0.00	0.00	0.01	0.00	0.00	0.00	0.01	0.00	0.00	0.00	0.01	0.01	0.00	0.00	0.00	0.00
55	Brudevold 1966* (2Fagents/2 abras)	PL vs DE	0.01	0.01	0.00	0.00	0.00	0.31	0.01	0.01	0.00	0.07	0.03	0.01	0.00	0.00	0.00	0.02	0.33	0.00	0.00	0.00	2.41	0.02	0.01	0.00	0.74	0.59	0.19	0.00	0.00	0.00
56	Hargreaves 1973(Age 1)	PL vs DE	0.01	0.09	0.00	0.00	0.00	0.99	0.01	0.01	0.00	0.28	0.15	0.02	0.00	0.00	0.00	0.00	0.00	0.00	0.00	0.00	0.01	0.00	0.00	0.00	0.00	0.00	0.00	0.00	0.00	0.00
57	Hanachowicz 1984	PL vs DE	0.01	0.51	0.00	0.00	0.01	4.26	0.02	0.01	0.02	1.35	0.74	0.06	0.00	0.01	0.01	0.00	0.78	0.00	0.00	0.01	4.48	0.00	0.00	0.01	1.60	1.27	0.50	0.00	0.01	0.01
58	Abrams 1980 (2abras)	PL vs DE	0.01	0.38	0.00	0.00	0.01	3.33	0.02	0.01	0.02	1.03	0.57	0.05	0.00	0.01	0.01	0.00	0.42	0.00	0.00	0.01	2.29	0.00	0.00	0.00	0.84	0.66	0.27	0.00	0.01	0.00
59	Glass 1978	PL vs DE	0.01	0.24	0.00	0.00	0.00	2.16	0.02	0.01	0.01	0.66	0.36	0.03	0.00	0.00	0.00	0.00	0.18	0.00	0.00	0.00	0.96	0.00	0.00	0.00	0.36	0.29	0.12	0.00	0.00	0.00
60	Andlaw 1975	PL vs DE	0.01	0.17	0.00	0.00	0.00	1.65	0.02	0.01	0.01	0.48	0.26	0.03	0.00	0.00	0.00	0.00	0.02	0.00	0.00	0.00	0.08	0.00	0.00	0.00	0.04	0.03	0.02	0.00	0.00	0.00
61	Howat 1978	PL vs DE	0.01	0.27	0.00	0.00	0.00	2.39	0.02	0.01	0.01	0.73	0.40	0.03	0.00	0.01	0.00	0.00	0.20	0.00	0.00	0.00	1.06	0.00	0.00	0.00	0.40	0.32	0.13	0.00	0.00	0.00
62	Marthaler 1965(1)	PL vs DE	0.01	0.00	0.00	0.00	0.00	0.07	0.01	0.00	0.00	0.01	0.00	0.00	0.00	0.00	0.00	0.02	0.48	0.00	0.00	0.00	3.36	0.02	0.01	0.00	1.06	0.84	0.29	0.00	0.00	0.00
63	Hodge 1980 (2Fagents/2 abrasives)	PL vs DE	0.01	0.35	0.00	0.00	0.01	3.04	0.02	0.01	0.02	0.94	0.52	0.04	0.00	0.01	0.01	0.00	0.38	0.00	0.00	0.01	2.10	0.00	0.00	0.00	0.77	0.61	0.24	0.00	0.01	0.00
64	Hargreaves 1973(Age 2)	PL vs DE	0.01	0.09	0.00	0.00	0.00	0.97	0.01	0.01	0.00	0.27	0.15	0.02	0.00	0.00	0.00	0.00	0.00	0.00	0.00	0.00	0.01	0.00	0.00	0.00	0.00	0.00	0.00	0.00	0.00	0.00
65	Held 1968 (site B)	PL vs DE	0.00	0.00	0.00	0.00	0.00	0.06	0.00	0.00	0.00	0.01	0.01	0.00	0.00	0.00	0.00	0.00	0.07	0.00	0.00	0.00	0.50	0.00	0.00	0.00	0.15	0.12	0.04	0.00	0.00	0.00

Study number	Trial	Treatments	Contribution to SMD (%)															Contribution to regression coefficient for treatment by year interaction (%)														
			PL vs. NT	DE vs. NT	RI vs. NT	GE vs. NT	VA vs. NT	DE vs. PL	RI vs. PL	GE vs. PL	VA vs. PL	RI vs. DE	GE vs. DE	VA vs. DE	GE vs. RI	VA vs. RI	VA vs. GE	PL vs. NT	DE vs. NT	RI vs. NT	GE vs. NT	VA vs. NT	DE vs. PL	RI vs. PL	GE vs. PL	VA vs. PL	RI vs. DE	GE vs. DE	VA vs. DE	GE vs. RI	VA vs. RI	VA vs. GE
66	Jackson 1967	PL vs DE	0.01	0.02	0.00	0.00	0.00	0.40	0.01	0.01	0.00	0.09	0.05	0.01	0.00	0.00	0.00	0.01	0.24	0.00	0.00	0.00	1.79	0.01	0.01	0.00	0.54	0.43	0.14	0.00	0.00	0.00
67	Fogels 1979 (2abras)	PL vs DE	0.01	0.24	0.00	0.00	0.00	2.26	0.02	0.01	0.01	0.68	0.37	0.04	0.00	0.00	0.00	0.00	0.10	0.00	0.00	0.00	0.49	0.00	0.00	0.00	0.20	0.16	0.07	0.00	0.00	0.00
68	Marthaler 1974	PL vs DE	0.00	0.04	0.00	0.00	0.00	0.44	0.01	0.00	0.00	0.12	0.06	0.01	0.00	0.00	0.00	0.00	0.01	0.00	0.00	0.00	0.13	0.00	0.00	0.00	0.03	0.03	0.01	0.00	0.00	0.00
69	Zacherl 1972	PL vs DE	0.01	0.13	0.00	0.00	0.00	1.29	0.01	0.01	0.00	0.37	0.20	0.02	0.00	0.00	0.00	0.00	0.00	0.00	0.00	0.00	0.01	0.00	0.00	0.00	0.01	0.00	0.00	0.00	0.00	0.00
70	Peterson 1967* (2Fagents/2abras)	PL vs DE	0.01	0.05	0.00	0.00	0.00	0.64	0.01	0.01	0.00	0.16	0.08	0.02	0.00	0.00	0.00	0.01	0.11	0.00	0.00	0.00	0.87	0.01	0.00	0.00	0.25	0.20	0.06	0.00	0.00	0.00
71	Naylor 1967	PL vs DE	0.01	0.01	0.00	0.00	0.00	0.31	0.01	0.01	0.00	0.07	0.03	0.01	0.00	0.00	0.00	0.02	0.33	0.00	0.00	0.00	2.41	0.02	0.01	0.00	0.74	0.59	0.19	0.00	0.00	0.00
72	Naylor 1979	PL vs DE	0.01	0.25	0.00	0.00	0.00	2.27	0.02	0.01	0.01	0.69	0.37	0.03	0.00	0.00	0.00	0.00	0.15	0.00	0.00	0.00	0.74	0.00	0.00	0.00	0.29	0.23	0.10	0.00	0.00	0.00
73	Koch 1967c	PL vs DE	0.00	0.01	0.00	0.00	0.00	0.18	0.00	0.00	0.00	0.04	0.02	0.00	0.00	0.00	0.00	0.00	0.06	0.00	0.00	0.00	0.44	0.00	0.00	0.00	0.13	0.10	0.03	0.00	0.00	0.00
74	Torell 1965a (age 1)	PL vs DE	0.01	0.02	0.00	0.00	0.00	0.31	0.01	0.01	0.00	0.07	0.04	0.01	0.00	0.00	0.00	0.01	0.18	0.00	0.00	0.00	1.35	0.01	0.00	0.00	0.41	0.32	0.11	0.00	0.00	0.00
75	Mainwaring 1983 (2Fagents)	PL vs DE	0.01	0.43	0.00	0.00	0.01	3.62	0.02	0.01	0.02	1.14	0.63	0.05	0.00	0.01	0.01	0.00	0.59	0.00	0.00	0.01	3.38	0.00	0.00	0.00	1.22	0.96	0.38	0.00	0.01	0.01
76	Di Maggio 1980	PL vs DE	0.00	0.06	0.00	0.00	0.00	0.48	0.00	0.00	0.00	0.15	0.08	0.01	0.00	0.00	0.00	0.00	0.07	0.00	0.00	0.00	0.39	0.00	0.00	0.00	0.14	0.11	0.04	0.00	0.00	0.00
77	James 1977	PL vs DE	0.01	0.17	0.00	0.00	0.00	1.66	0.02	0.01	0.01	0.49	0.26	0.03	0.00	0.00	0.00	0.00	0.02	0.00	0.00	0.00	0.08	0.00	0.00	0.00	0.04	0.03	0.02	0.00	0.00	0.00
78	Torell 1965b (age 2)	PL vs DE	0.01	0.02	0.00	0.00	0.00	0.33	0.01	0.01	0.00	0.08	0.04	0.01	0.00	0.00	0.00	0.01	0.20	0.00	0.00	0.00	1.47	0.01	0.00	0.00	0.44	0.35	0.11	0.00	0.00	0.00
79	Fanning 1968	PL vs DE	0.01	0.05	0.00	0.00	0.00	0.64	0.01	0.01	0.00	0.16	0.08	0.02	0.00	0.00	0.00	0.01	0.11	0.00	0.00	0.00	0.87	0.01	0.00	0.00	0.25	0.20	0.06	0.00	0.00	0.00
80	Slack 1971(2Fagents/2abras)	PL vs DE	0.01	0.06	0.00	0.00	0.00	0.78	0.01	0.01	0.00	0.21	0.11	0.02	0.00	0.00	0.00	0.01	0.06	0.00	0.00	0.00	0.53	0.01	0.00	0.00	0.15	0.12	0.03	0.00	0.00	0.00
81	Held 1968 (site A)	PL vs DE	0.00	0.01	0.00	0.00	0.00	0.10	0.00	0.00	0.00	0.02	0.01	0.00	0.00	0.00	0.00	0.00	0.06	0.00	0.00	0.00	0.46	0.00	0.00	0.00	0.14	0.11	0.04	0.00	0.00	0.00
82	Hargreaves 1973(Age 3)	PL vs DE	0.01	0.09	0.00	0.00	0.00	0.98	0.01	0.01	0.00	0.28	0.15	0.02	0.00	0.00	0.00	0.00	0.00	0.00	0.00	0.00	0.01	0.00	0.00	0.00	0.00	0.00	0.00	0.00	0.00	0.00

Study number	Trial	Treatments	Contribution to SMD (%)															Contribution to regression coefficient for treatment by year interaction (%)														
			PL vs. NT	DE vs. NT	RI vs. NT	GE vs. NT	VA vs. NT	DE vs. PL	RI vs. PL	GE vs. PL	VA vs. PL	RI vs. DE	GE vs. DE	VA vs. DE	GE vs. RI	VA vs. RI	VA vs. GE	PL vs. NT	DE vs. NT	RI vs. NT	GE vs. NT	VA vs. NT	DE vs. PL	RI vs. PL	GE vs. PL	VA vs. PL	RI vs. DE	GE vs. DE	VA vs. DE	GE vs. RI	VA vs. RI	VA vs. GE
83	Zacherl 1970* (2)	PL vs DE	0.01	0.03	0.00	0.00	0.00	0.46	0.01	0.01	0.00	0.11	0.06	0.01	0.00	0.00	0.00	0.01	0.15	0.00	0.00	0.00	1.15	0.01	0.00	0.00	0.34	0.27	0.09	0.00	0.00	0.00
84	Marthaler 1965(2)	PL vs DE	0.00	0.00	0.00	0.00	0.00	0.03	0.00	0.00	0.00	0.00	0.00	0.00	0.00	0.00	0.00	0.01	0.24	0.00	0.00	0.00	1.66	0.01	0.00	0.00	0.52	0.41	0.14	0.00	0.00	0.00
85	Buhe 1984 (2Fconc)	PL vs DE	0.01	0.39	0.00	0.00	0.01	3.37	0.02	0.01	0.02	1.05	0.57	0.05	0.00	0.01	0.01	0.00	0.42	0.00	0.00	0.01	2.32	0.00	0.00	0.00	0.85	0.67	0.27	0.00	0.01	0.00
86	DePaola 1980	PL vs RI vs GE	1.01	0.83	1.47	0.92	0.36	0.03	1.89	5.19	0.07	1.20	4.20	0.06	5.42	0.02	0.97	0.09	0.06	0.14	0.36	0.08	0.01	0.64	0.99	0.17	0.36	0.71	0.15	1.06	0.29	0.01
87	Heidmann 1992	PL vs RI	0.15	0.10	0.03	0.05	0.00	0.02	1.11	0.05	0.05	0.64	0.02	0.03	0.18	0.00	0.01	0.35	0.22	0.87	0.12	0.01	0.03	6.43	0.11	0.10	3.82	0.04	0.06	2.07	0.51	0.02
88	Horowitz 1971(grade 1)	PL vs RI	0.07	0.03	0.57	0.02	0.01	0.03	3.76	0.02	0.05	2.33	0.00	0.04	1.02	0.07	0.03	0.04	0.03	0.48	0.01	0.01	0.00	2.25	0.02	0.06	1.45	0.01	0.05	0.81	0.13	0.03
89	Laswell 1975 (2Fconc/2freq)	PL vs RI	0.08	0.04	0.37	0.03	0.01	0.02	2.87	0.03	0.05	1.76	0.01	0.03	0.73	0.05	0.02	0.00	0.00	0.11	0.00	0.01	0.00	0.36	0.00	0.01	0.25	0.00	0.01	0.14	0.02	0.01
90	Driscoll 1982 (2Fconc/2freq)	PL vs RI	0.11	0.06	0.17	0.04	0.00	0.02	1.94	0.04	0.05	1.16	0.01	0.03	0.43	0.02	0.02	0.07	0.04	0.06	0.02	0.01	0.01	0.70	0.02	0.01	0.39	0.00	0.00	0.20	0.07	0.00
91	Packer 1975 (2Fconc/2freq)	PL vs RI	0.08	0.04	0.37	0.03	0.01	0.02	2.80	0.03	0.05	1.72	0.01	0.03	0.72	0.05	0.02	0.00	0.00	0.10	0.00	0.01	0.00	0.35	0.00	0.01	0.24	0.00	0.01	0.14	0.02	0.01
92	Poulsen 1984	PL vs RI	0.11	0.07	0.10	0.04	0.00	0.02	1.49	0.04	0.04	0.88	0.01	0.03	0.30	0.01	0.02	0.12	0.07	0.19	0.04	0.01	0.01	1.68	0.04	0.02	0.97	0.01	0.01	0.52	0.15	0.00
93	Horowitz 1971a(grade 5)	PL vs RI	0.06	0.03	0.52	0.02	0.01	0.03	3.43	0.02	0.05	2.13	0.00	0.03	0.93	0.07	0.02	0.04	0.03	0.43	0.01	0.01	0.00	2.05	0.02	0.06	1.32	0.01	0.05	0.74	0.12	0.03
94	Radike 1973	PL vs RI	0.10	0.05	0.55	0.03	0.01	0.03	4.04	0.03	0.06	2.49	0.01	0.05	1.05	0.07	0.03	0.01	0.01	0.23	0.00	0.01	0.00	0.90	0.00	0.03	0.60	0.00	0.03	0.34	0.05	0.02
95	Petersson 1998	PL vs RI	0.11	0.08	0.06	0.03	0.00	0.01	0.02	0.04	0.02	0.00	0.02	0.01	0.01	0.01	0.00	0.73	0.45	2.43	0.24	0.00	0.06	16.10	0.24	0.29	9.75	0.08	0.19	5.32	1.21	0.09
96	Duany 1981(3Fconc)	PL vs RI	0.12	0.07	0.19	0.04	0.00	0.02	2.17	0.04	0.05	1.30	0.01	0.04	0.48	0.02	0.02	0.07	0.04	0.07	0.03	0.01	0.01	0.78	0.02	0.01	0.43	0.01	0.00	0.23	0.08	0.00
97	Ringelberg 1982 (2Fconc/2freq)	PL vs RI	0.13	0.08	0.12	0.04	0.00	0.02	1.79	0.05	0.05	1.06	0.02	0.04	0.36	0.01	0.02	0.14	0.08	0.23	0.05	0.01	0.02	2.02	0.04	0.03	1.17	0.01	0.01	0.63	0.18	0.00
98	Spets-Happonen 1991	PL vs RI	0.07	0.05	0.00	0.02	0.00	0.01	0.34	0.02	0.02	0.19	0.01	0.01	0.04	0.00	0.01	0.21	0.13	0.56	0.07	0.00	0.02	4.00	0.07	0.07	2.39	0.02	0.04	1.30	0.31	0.02

Study number	Trial	Treatments	Contribution to SMD (%)															Contribution to regression coefficient for treatment by year interaction (%)														
			PL vs. NT	DE vs. NT	RI vs. NT	GE vs. NT	VA vs. NT	DE vs. PL	RI vs. PL	GE vs. PL	VA vs. PL	RI vs. DE	GE vs. DE	VA vs. DE	GE vs. RI	VA vs. RI	VA vs. GE	PL vs. NT	DE vs. NT	RI vs. NT	GE vs. NT	VA vs. NT	DE vs. PL	RI vs. PL	GE vs. PL	VA vs. PL	RI vs. DE	GE vs. DE	VA vs. DE	GE vs. RI	VA vs. RI	VA vs. GE
99	Heifetz 1982 (2Fconc/2freq)	PL vs RI	0.11	0.06	0.22	0.04	0.00	0.02	2.27	0.04	0.05	1.37	0.01	0.04	0.51	0.03	0.02	0.05	0.03	0.02	0.02	0.01	0.01	0.37	0.01	0.00	0.20	0.00	0.00	0.10	0.04	0.00
100	Koch 1967b	PL vs RI	0.06	0.02	0.89	0.02	0.02	0.03	5.09	0.02	0.06	3.19	0.00	0.04	1.47	0.12	0.03	0.18	0.12	1.31	0.05	0.02	0.01	6.87	0.07	0.17	4.34	0.03	0.13	2.41	0.44	0.08
101	Molina 1987	PL vs RI	0.11	0.07	0.02	0.04	0.00	0.02	0.82	0.04	0.03	0.47	0.02	0.03	0.13	0.00	0.01	0.26	0.16	0.64	0.09	0.00	0.03	4.74	0.08	0.07	2.81	0.03	0.05	1.52	0.38	0.02
102	McConchie 1977(2Fconc)	PL vs RI	0.10	0.05	0.54	0.03	0.01	0.03	3.97	0.03	0.06	2.45	0.01	0.05	1.04	0.07	0.03	0.01	0.01	0.22	0.00	0.01	0.00	0.88	0.00	0.03	0.59	0.00	0.03	0.34	0.04	0.02
103	Koch 1967a	PL vs RI	0.06	0.02	0.89	0.02	0.02	0.03	5.07	0.02	0.06	3.18	0.00	0.04	1.46	0.12	0.03	0.18	0.12	1.30	0.05	0.02	0.01	6.84	0.07	0.17	4.32	0.03	0.13	2.40	0.44	0.08
104	Moreira 1972 (3Freq)	PL vs RI	0.05	0.03	0.41	0.02	0.01	0.02	2.75	0.02	0.04	1.70	0.00	0.03	0.74	0.05	0.02	0.02	0.01	0.28	0.00	0.01	0.00	1.27	0.01	0.04	0.83	0.01	0.03	0.46	0.07	0.02
105	Finn 1975(2Fconc)	PL vs RI	0.09	0.05	0.38	0.03	0.01	0.03	3.04	0.03	0.05	1.86	0.01	0.04	0.76	0.05	0.02	0.00	0.00	0.06	0.00	0.01	0.00	0.16	0.00	0.01	0.12	0.00	0.01	0.07	0.00	0.01
106	van Wyk 1986 (2Fconc)	PL vs RI	0.12	0.08	0.06	0.04	0.00	0.02	1.28	0.04	0.04	0.74	0.02	0.03	0.23	0.01	0.01	0.21	0.13	0.44	0.07	0.01	0.02	3.45	0.06	0.05	2.03	0.02	0.03	1.10	0.29	0.01
107	Heifetz 1973(2Fagents)	PL vs RI	0.08	0.04	0.53	0.03	0.01	0.03	3.74	0.03	0.06	2.31	0.01	0.04	0.99	0.07	0.03	0.02	0.01	0.29	0.00	0.01	0.00	1.25	0.01	0.04	0.82	0.01	0.03	0.47	0.07	0.02
108	DePaola 1977(2Fagents)	PL vs RI	0.10	0.05	0.28	0.03	0.01	0.02	2.55	0.03	0.05	1.55	0.01	0.04	0.61	0.04	0.02	0.01	0.01	0.00	0.01	0.01	0.00	0.01	0.00	0.00	0.00	0.00	0.00	0.00	0.00	0.00
109	Bastos 1989(2Fagents)	PL vs RI	0.10	0.06	0.16	0.03	0.00	0.02	1.85	0.04	0.04	1.11	0.01	0.03	0.41	0.02	0.02	0.06	0.04	0.06	0.02	0.01	0.01	0.66	0.02	0.01	0.37	0.00	0.00	0.20	0.06	0.00
110	Rugg-Gunn 1973	PL vs RI	0.08	0.04	0.55	0.03	0.01	0.03	3.84	0.03	0.06	2.37	0.01	0.04	1.02	0.07	0.03	0.02	0.01	0.30	0.00	0.01	0.00	1.29	0.01	0.04	0.85	0.01	0.04	0.48	0.07	0.02
111	Gallagher 1974	PL vs RI	0.09	0.05	0.53	0.03	0.01	0.03	3.89	0.03	0.06	2.40	0.01	0.04	1.01	0.07	0.03	0.01	0.01	0.22	0.00	0.01	0.00	0.86	0.00	0.03	0.58	0.00	0.03	0.33	0.04	0.02
112	Gisselsson 1999 (2Fconc/2Fagents)	PL vs GE	0.06	0.05	0.02	0.32	0.66	0.00	0.03	0.92	0.50	0.01	0.73	0.51	0.53	0.57	1.06	4.27	3.33	2.28	4.09	0.06	0.03	0.51	28.16	2.63	0.23	19.87	2.22	14.96	1.67	1.14
113	Shern 1976(2Fagents/2intervals)	PL vs GE	1.63	1.31	1.15	0.73	0.21	0.03	0.07	6.56	0.02	0.01	5.16	0.01	4.29	0.00	0.63	0.04	0.04	0.05	0.00	0.04	0.00	0.01	0.04	0.01	0.00	0.04	0.01	0.05	0.00	0.04
114	Szwjeda 1972	PL vs GE	2.05	1.66	1.50	0.73	0.10	0.03	0.07	7.62	0.12	0.01	6.00	0.09	5.04	0.08	0.45	0.59	0.49	0.44	0.23	0.03	0.00	0.01	2.64	0.10	0.00	1.92	0.09	1.62	0.07	0.26
115	Olivier 1992	PL vs GE	0.45	0.35	0.27	0.50	0.50	0.0	0.0	2.68	0.17	0.0	2.1	0.18	1.6	0.22	0.97	1.62	1.25	0.80	1.79	0.06	0.01	0.25	11.48	1.20	0.11	8.07	1.01	5.95	0.75	0.39

Study number	Trial	Treatments	Contribution to SMD (%)															Contribution to regression coefficient for treatment by year interaction (%)														
			PL vs. NT	DE vs. NT	RI vs. NT	GE vs. NT	VA vs. NT	DE vs. PL	RI vs. PL	GE vs. PL	VA vs. PL	RI vs. DE	GE vs. DE	VA vs. DE	GE vs. RI	VA vs. RI	VA vs. GE	PL vs. NT	DE vs. NT	RI vs. NT	GE vs. NT	VA vs. NT	DE vs. PL	RI vs. PL	GE vs. PL	VA vs. PL	RI vs. DE	GE vs. DE	VA vs. DE	GE vs. RI	VA vs. RI	VA vs. GE
								1	5			1	1		8																	
116	Cons 1970	PL vs GE	3.11	2.52	2.31	0.96	0.07	0.05	0.09	10.98	0.32	0.01	8.64	0.27	7.30	0.24	0.44	1.81	1.48	1.25	0.94	0.04	0.00	0.06	9.08	0.48	0.03	6.53	0.41	5.33	0.34	0.71
117	Trubman 1973	PL vs GE	1.90	1.53	1.38	0.70	0.12	0.03	0.07	7.14	0.09	0.01	5.62	0.07	4.71	0.05	0.46	0.42	0.35	0.33	0.14	0.03	0.00	0.00	1.77	0.05	0.00	1.29	0.05	1.11	0.04	0.20
118	Heifetz 1970	PL vs GE	2.31	1.87	1.70	0.76	0.08	0.04	0.07	8.35	0.18	0.01	6.57	0.15	5.54	0.13	0.40	1.00	0.82	0.71	0.47	0.03	0.00	0.02	4.78	0.22	0.01	3.45	0.19	2.86	0.16	0.41
119	Hagan 1985(2Fconc)	PL vs GE	0.65	0.52	0.43	0.49	0.34	0.01	0.05	3.24	0.05	0.01	2.55	0.05	2.06	0.07	0.73	0.53	0.40	0.23	0.71	0.05	0.01	0.11	4.16	0.50	0.05	2.90	0.42	2.08	0.31	0.11
120	Horowitz 1974	PL vs GE	1.91	1.55	1.40	0.66	0.08	0.03	0.06	7.01	0.13	0.01	5.51	0.11	4.64	0.09	0.37	0.69	0.56	0.50	0.30	0.03	0.00	0.01	3.19	0.14	0.01	2.31	0.12	1.93	0.10	0.29
121	Tewari 1990	PL vs VA	0.74	0.53	0.29	0.28	1.58	0.05	0.28	0.22	2.85	0.09	0.11	2.67	0.01	2.41	2.10	1.15	0.78	0.26	0.29	0.22	0.05	0.69	0.52	0.04	0.29	0.24	0.02	0.00	0.02	0.02
122	Borutta 1991 (2Fconc/2freq)	PL vs VA	0.24	0.19	0.11	0.05	2.39	0.01	0.07	0.14	1.81	0.03	0.09	1.83	0.03	1.99	2.03	3.16	2.43	0.90	0.36	4.82	0.03	1.46	2.44	12.15	0.78	1.56	10.64	0.21	8.44	7.49
123	Clark 1985 (2Fconc)	PL vs VA	0.83	0.59	0.33	0.34	3.09	0.06	0.32	0.23	4.93	0.10	0.10	4.67	0.01	4.31	3.84	0.84	0.55	0.18	0.26	0.93	0.06	0.55	0.30	0.17	0.21	0.11	0.21	0.00	0.49	0.48
124	Axelsson 1987	DE vs RI	0.03	0.36	0.09	0.02	0.00	1.28	0.79	0.01	0.00	2.12	0.35	0.07	0.21	0.03	0.00	0.03	0.37	0.03	0.01	0.00	1.10	0.32	0.01	0.01	1.24	0.41	0.17	0.09	0.02	0.00
125	Petersson 1985	DE vs VA	0.65	1.59	0.29	0.35	6.65	1.68	0.20	0.11	9.07	1.40	0.82	10.49	0.00	8.40	7.68	0.11	0.77	0.01	0.11	3.70	1.68	0.12	0.00	3.24	1.19	0.50	1.74	0.05	3.59	3.35
126	Kirkegaard 1986	RI vs VA	0.43	0.32	0.90	0.23	8.59	0.02	0.40	0.07	10.88	0.40	0.03	10.57	0.37	11.99	9.42	0.02	0.00	0.22	0.05	4.64	0.02	0.35	0.02	4.77	0.33	0.04	4.48	0.08	3.56	4.61
127	Koch 1979	RI vs VA	0.47	0.34	0.96	0.27	12.11	0.03	0.40	0.07	15.01	0.41	0.03	14.59	0.37	16.31	13.14	0.00	0.02	0.01	0.05	7.64	0.03	0.07	0.13	8.69	0.10	0.16	8.12	0.01	7.66	7.88
128	Brunn 1985	RI vs VA	0.23	0.18	0.52	0.11	2.27	0.01	0.27	0.05	3.11	0.24	0.03	3.01	0.25	3.60	2.60	0.16	0.11	0.93	0.04	0.61	0.01	1.03	0.06	0.31	0.77	0.03	0.31	0.79	0.02	0.48
129	Seppa 1987	RI vs VA	0.00	0.00	0.01	0.01	6.58	0.00	0.03	0.01	6.85	0.01	0.02	6.71	0.04	6.62	6.54	1.43	1.30	5.97	0.02	10.04	0.01	5.14	1.88	17.01	3.22	1.50	15.58	7.05	24.36	12.13
130	Seppa 1995	GE vs VA	0.00	0.00	0.00	0.45	4.31	0.00	0.00	0.56	4.46	0.00	0.44	4.38	0.40	4.44	3.09	0.11	0.09	0.02	6.13	10.44	0.00	0.61	7.56	13.07	0.41	5.54	11.81	7.73	10.26	25.75

Table S9: Percentage contribution of each trial to each SMD and coefficient using the existing methods proposed by Riley et al for the fluoride dataset.[7]

DE: dentifrice; GE: gel; NT: no treatment; PL: placebo; RI: rinse; SMD: standardised mean difference; VA: varnish.

.

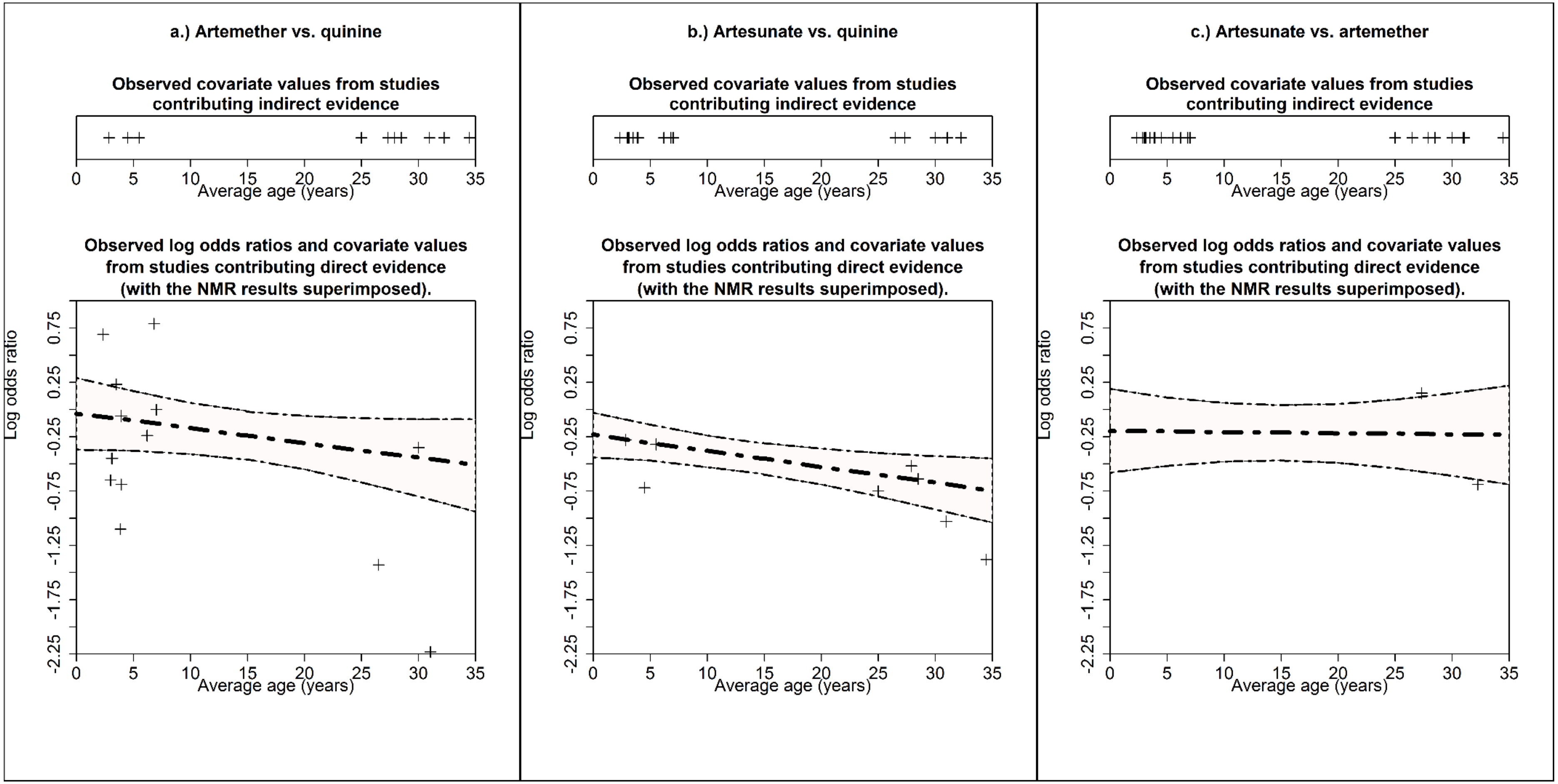

Figure S1: NMR plot for the malaria dataset.

The bold dot-dash line is the log odds ratio and the two dashed lines are the upper and lower 95% credibility intervals estimated by the model. Points represent the trials that contribute to the model estimates; points for trials that contribute direct evidence are displayed in the bottom section and points from trials that contribute indirect evidence are displayed in the top section.

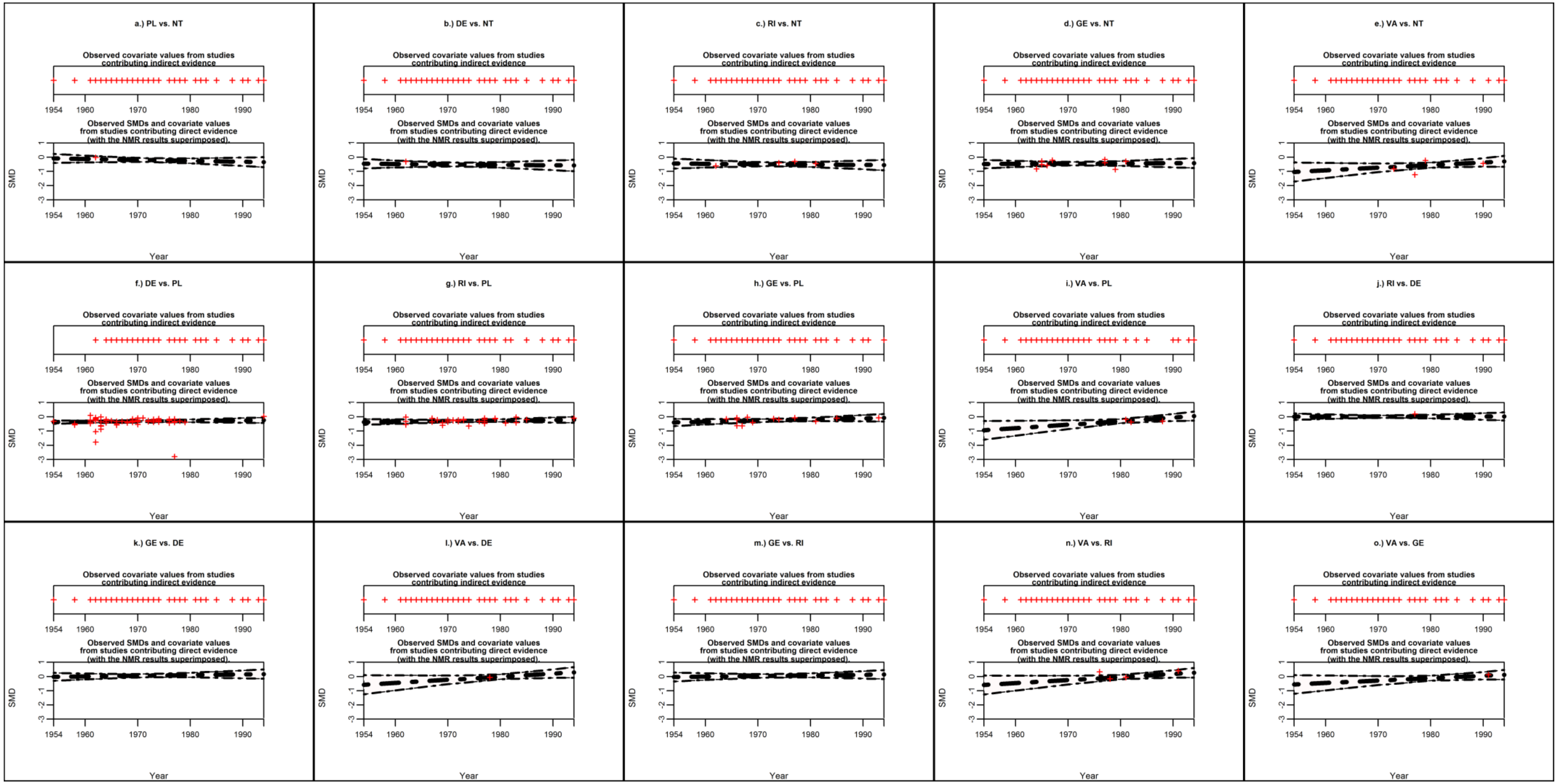

Figure S2: NMR plot for the fluoride dataset.

The bold dot-dash line is the SMD and the two dashed lines are the upper and lower 95% credibility intervals estimated by the model. Points represent the trials that contribute to the model estimates; points for trials that contribute direct evidence are displayed in the bottom section and points from trials that contribute indirect evidence are displayed in the top section.
